# Fatigue in early multiple sclerosis: MRI metrics of neuroinflammation, relapse and neurodegeneration

**DOI:** 10.1093/braincomms/fcae278

**Published:** 2024-08-14

**Authors:** Rozanna Meijboom, Peter Foley, Niall J J MacDougall, Yair Mina, Elizabeth N York, Agniete Kampaite, Daisy Mollison, Patrick K A Kearns, Nicole White, Michael J Thrippleton, Katy Murray, Maria del C Valdés Hernández, Daniel S Reich, Peter Connick, Steven Jacobson, Govind Nair, Siddharthan Chandran, Adam D Waldman

**Affiliations:** Centre for Clinical Brain Sciences, University of Edinburgh, Edinburgh EH16 4SB, UK; Edinburgh Imaging, University of Edinburgh, Edinburgh EH16 4SB, UK; Anne Rowling Regenerative Neurology Clinic, University of Edinburgh, Edinburgh EH16 4SB, UK; Centre for Clinical Brain Sciences, University of Edinburgh, Edinburgh EH16 4SB, UK; Anne Rowling Regenerative Neurology Clinic, University of Edinburgh, Edinburgh EH16 4SB, UK; Anne Rowling Regenerative Neurology Clinic, University of Edinburgh, Edinburgh EH16 4SB, UK; Department of Neurology, Institute of Neurological Sciences, Queen Elizabeth University Hospital, Glasgow G51 4TF, UK; Viral Immunology Section, National Institute of Neurological Disorders and Stroke, National Institutes of Health, Bethesda, MD 20824, USA; Sackler Faculty of Medicine, Tel-Aviv University, Tel-Aviv 69978, Israel; Centre for Clinical Brain Sciences, University of Edinburgh, Edinburgh EH16 4SB, UK; Edinburgh Imaging, University of Edinburgh, Edinburgh EH16 4SB, UK; Anne Rowling Regenerative Neurology Clinic, University of Edinburgh, Edinburgh EH16 4SB, UK; Centre for Clinical Brain Sciences, University of Edinburgh, Edinburgh EH16 4SB, UK; Edinburgh Imaging, University of Edinburgh, Edinburgh EH16 4SB, UK; Centre for Clinical Brain Sciences, University of Edinburgh, Edinburgh EH16 4SB, UK; Anne Rowling Regenerative Neurology Clinic, University of Edinburgh, Edinburgh EH16 4SB, UK; Centre for Clinical Brain Sciences, University of Edinburgh, Edinburgh EH16 4SB, UK; Edinburgh Imaging, University of Edinburgh, Edinburgh EH16 4SB, UK; Centre for Clinical Brain Sciences, University of Edinburgh, Edinburgh EH16 4SB, UK; Edinburgh Imaging, University of Edinburgh, Edinburgh EH16 4SB, UK; Anne Rowling Regenerative Neurology Clinic, University of Edinburgh, Edinburgh EH16 4SB, UK; Department of Neurology, Forth Valley Royal Hospital, Larbert FK5 4WR, UK; Centre for Clinical Brain Sciences, University of Edinburgh, Edinburgh EH16 4SB, UK; Edinburgh Imaging, University of Edinburgh, Edinburgh EH16 4SB, UK; Translational Neuroradiology Section, National Institute of Neurological Disorders and Stroke, National Institutes of Health, Bethesda, MD 20824, USA; Centre for Clinical Brain Sciences, University of Edinburgh, Edinburgh EH16 4SB, UK; Anne Rowling Regenerative Neurology Clinic, University of Edinburgh, Edinburgh EH16 4SB, UK; Viral Immunology Section, National Institute of Neurological Disorders and Stroke, National Institutes of Health, Bethesda, MD 20824, USA; Translational Neuroradiology Section, National Institute of Neurological Disorders and Stroke, National Institutes of Health, Bethesda, MD 20824, USA; Quantitative MRI Core Facility, National Institute of Neurological Disorders and Stroke, National Institutes of Health, Bethesda, MD 20824, USA; Centre for Clinical Brain Sciences, University of Edinburgh, Edinburgh EH16 4SB, UK; Anne Rowling Regenerative Neurology Clinic, University of Edinburgh, Edinburgh EH16 4SB, UK; Centre for Clinical Brain Sciences, University of Edinburgh, Edinburgh EH16 4SB, UK; Edinburgh Imaging, University of Edinburgh, Edinburgh EH16 4SB, UK; Anne Rowling Regenerative Neurology Clinic, University of Edinburgh, Edinburgh EH16 4SB, UK

**Keywords:** multiple sclerosis, fatigue, magnetic resonance imaging, neurodegeneration, neuroinflammation

## Abstract

Multiple sclerosis (MS) is a neuroinflammatory and neurodegenerative disease affecting the brain and spinal cord. Fatigue is a common disabling symptom from MS onset, however the mechanisms by which underlying disease processes cause fatigue remain unclear. Improved pathophysiological understanding offers potential for improved treatments for MS-related fatigue. MRI provides insights into *in vivo* neuroinflammatory activity and neurodegeneration, although existing evidence for imaging correlates of MS fatigue is mixed. We explore associations between fatigue and MRI measures in the brain and spinal cord to identify neuroinflammatory and regional neurodegenerative substrates of fatigue in early relapsing–remitting MS (RRMS). Recently diagnosed (<6 months), treatment-naive people with RRMS (*n* = 440) were recruited to a longitudinal multi-centre nationally representative cohort study. Participants underwent 3-Tesla brain MRI at baseline and one year. We calculated global and regional white and grey matter volumes, white matter lesion (WML) load and upper cervical spinal cord cross-sectional area levels C2–3, and assessed new/enlarging WMLs visually. Participants were classed as fatigued or non-fatigued at baseline according to the Fatigue Severity Scale (>/≤36). Disability and depression were assessed with the expanded-disability status scale and Patient Health Questionnaire, respectively. MRI measures were compared between fatigue groups, both cross-sectionally and longitudinally, using regression analyses. Higher disability and depression scores were observed for participants with fatigue, with a higher number of fatigued participants receiving disease-modifying treatments at follow-up. Structural MRI data for brain were available for *n* = 313 (45% fatigued) and for spinal cord for *n* = 324 (46% fatigued). Cervical spinal cord cross-sectional area 2–3, white and grey matter volumes decreased, and WML volume increased, over time for both groups (*q* < 0.05). However, no significant between-group differences in these measures were found either cross-sectionally or longitudinally (*q* > 0.05). The presence of new/enlarging WMLs (49% in fatigued; 51% in non-fatigued) at follow-up also did not differ between groups (*q* > 0.05). Our results suggest that fatigue is not driven by neuroinflammation or neurodegeneration measurable by current structural MRI in early RRMS. This novel negative finding in a large multi-centre cohort of people with recently diagnosed RRMS helps to resolve uncertainty in existing literature. Notably, we showed that fatigue is prevalent in patients without brain radiological relapse, who may be considered to have inactive disease. This suggests that symptom detection and treatment should remain a clinical priority regardless of neuroinflammatory disease activity. More sensitive objective biomarkers are needed to elucidate fatigue mechanisms in RRMS, and ultimately facilitate development of effective targeted treatments for this important ‘hidden disability’.

## Teaser

Meijboom *et al*. show that fatigue in early multiple sclerosis is not driven by neuroinflammation or neurodegeneration measurable by structural MRI, and is prevalent in absence of radiological relapse. More sensitive biomarkers are needed to elucidate fatigue mechanisms in multiple sclerosis and facilitate effective treatment development.

## Graphical Abstract

**Graphical Abstract:**
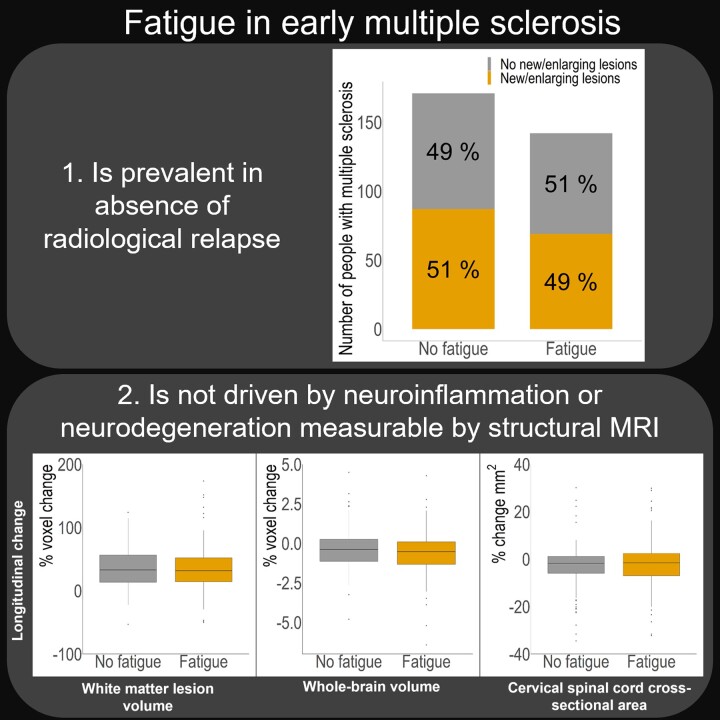

## Introduction

### Multiple sclerosis

Multiple sclerosis (MS) is a common neuroinflammatory and neurodegenerative autoimmune disorder affecting both the brain and spinal cord.^1^ A total of 2.8 million^2^ people are estimated to have MS worldwide, and a cure is not yet available. The majority of people with MS (pwMS) initially follow a relapsing–remitting disease course [relapsing–remitting MS (RRMS)], albeit with highly heterogeneous disease severity and trajectory across individuals. PwMS can present with a broad spectrum of symptoms, including motor and visual dysfunction. Less externally visible symptoms—or hidden disability—such as pain, depression, cognitive impairment and fatigue are also strongly associated with MS and have a major impact on quality of life.^3^

### Multiple sclerosis and fatigue

Reported fatigue is a common and disabling early symptom for people newly diagnosed with RRMS, affecting 60–80% of cases, and precede diagnosis as part of an MS prodrome.^4,5^ Reported (or perceived) fatigue (typically measured by questionnaire) is distinct from ‘fatigability’ (sometimes called performance-related fatigue) where objective performance-related changes, for example in motor function and cognition are measured in response to activity.^6^ MS-related fatigue may also be primary (i.e. MS-driven) or secondary, related to co-morbidities such as sleep disorders, depression and/or disability.^7^ Mechanisms underlying fatigue may differ between early RRMS and long-standing progressive MS.

Fatigue is difficult to define and measure objectively; suggested definitions include ‘reversible motor and cognitive impairment, with reduced motivation, and desire to rest’^8^ or ‘a subjective lack of physical and/or mental energy that is perceived by the individual or caregiver to interfere with usual or desired activity’,^9^ and numerous measures are available for fatigue assessment.^9^ Pathophysiological mechanisms underlying fatigue may be central (related to impairment in neuronal energetics, function or conduction), peripheral (for example, muscular) or systemic (such as immune system dysregulation),^10^ but remain poorly understood. Current pharmacological treatment options have limited efficacy.^11^ A better understanding of mechanisms underlying fatigue in early stages of MS may aid development of more targeted treatments, as neuroinflammatory or neurodegeneration-driven processes may benefit from different therapeutic strategies.

### Structural MRI and fatigue

MRI is widely used in the clinical setting, and allows neuroinflammation and neurodegeneration to be monitored *in vivo*. Specifically, white matter lesion (WML) volume measurements from fluid-attenuated inversion recovery (FLAIR) provide an indicator of cumulative inflammatory disease burden, and the presence of new/enlarging WMLs signals interval inflammatory relapse. Brain atrophy, quantified from tissue volume loss over time from high resolution T_1_-weighted (T1W) images, is recognized as a downstream marker of neurodegeneration.^12^

Current literature on associations between fatigue and structural MRI in early MS is limited and findings are variable, highlighting important gaps in understanding. Some brain studies in early RRMS observed associations between fatigue and cerebellar,^13^ thalamic,^14^ basal ganglia^15^ and pontine^15^ volumes, and regional cortical surface area.^16^ Additionally, brain studies in RRMS at varying disease stages have shown associations between fatigue and tissue volume/thickness in the basal ganglia,^17^ cerebellum,^13^ thalamus^18^ and some cortical gyri,^17^ and between fatigue and WML load in some brain regions.^19^ A recent systematic review, however, showed that nearly half of studies assessing fatigue and structural brain imaging across all stages of RRMS found no significant associations.^20^ Methodological differences between studies and cohort characteristics may contribute to such discrepant results, and it is unclear whether specific anatomical brain regions play a role in fatigue in early RRMS. Comprehensive analysis in an appropriate well-characterized cohort is therefore required to understand the contributions of inflammation and regional neurodegeneration to fatigue.

The role of spinal cord atrophy underlying fatigue in the early stages of RRMS has not been explored; of the few published studies including various RRMS disease stages, the majority found no relationship.^21,22^ The degree to which neuroinflammation and neurodegeneration in the spinal cord contribute to fatigue in early RRMS remains unclear.

### Study aim

Current evidence on central mechanisms of fatigue in RRMS is inconclusive, and the literature on spinal cord contributions and early disease is sparse. In the context of the need to develop targeted treatments for this major disabling symptom, this study aims to identify structural MRI measures of neuroinflammation and neurodegeneration as potential substrates of fatigue in early RRMS. We hypothesized that reported fatigue is associated with structural brain and cervical spinal cord MRI markers of neuroinflammation and/or neurodegeneration in recently diagnosed RRMS.

## Methods

### Participants

Participants with a recent RRMS disease onset^1^ were recruited to FutureMS, a longitudinal inception cohort study, across five centres in Scotland: Aberdeen, Dundee, Edinburgh, Inverness and Glasgow.^23,24^ Disease onset was defined as date of clinical diagnosis of RRMS, due to difficulty being certain that historically reported symptoms are due to MS.^1^ Inclusion criteria were a diagnosis of RRMS < 6 months prior to study inclusion, capacity to provide informed consent and ≥18 years old. Exclusion criteria included MRI contraindications, previous or current clinical trial participation or receiving disease-modifying therapies (DMTs) prior to baseline assessment (DMTs post-baseline assessment were ‘not’ an exclusion criteria). Study visits took place at baseline and 1-year follow-up, and participants underwent brain MRI and clinical assessments on the same day at both time points. FutureMS recruited 440 participants of which 354 participants had available structural MRI for analysis at both time points (see Table 1 for an overview of data exclusions).

**Table 1:** Overview of FutureMS data exclusions

Total number of FutureMS participants	440
*Exclusion reasons*	*No. excluded*
Data not available at both time points • Provided contact details at baseline no longer accurate for follow-up • Moved away from Scotland since baseline visit • Inability to attend the follow-up visit due to the COVID-19 pandemic	49
Recruitment to the study in error	9
Different MRI protocols at baseline and follow-up	9
Follow-up took place more than 14 months after baseline	5
Did not pass initial quality check	3
3 T MRI not performed	11
**Total number of available MRI data**	**354**

Ethical approval was given from the South East Scotland Research Ethics Committee 02 (reference 15/SS/0233). The study was conducted in accordance with the Declaration of Helsinki and ICH guidelines on Good Clinical Practice. All participants gave written informed consent before study entry, and data were anonymized with unique study identifiers.

### Clinical assessment

Fatigue and depression assessment was performed by medical professionals, using the Fatigue Severity Scale^25^ (FSS) and the Patient Health Questionnaire^26^ (PHQ-9), respectively. These scales were selected for the FutureMS study for obtaining a metric of general fatigue and depression in pwMS. The FSS comprises nine items, each scored from 1–7, with a sum score ranging from 9 to 63 (higher scores reflecting higher fatigue severity). Participants with an FSS score of 36 or lower at baseline were classed as ‘not significantly fatigued’; those with a score of over 36 at baseline were classed as ‘significantly fatigued’.^27^ This dichotomization was performed for analysis purposes due to the non-linear nature of the FSS scale. The PHQ-9 is a series of nine questions scored between 0 and 3, with a maximum total score of 27 (indicating severe depression). In case of missing values, multiple imputation by chained equations using predictive mean matching^28^ was applied. Reasons for missing values were typically logistical (e.g. patient ran out of time to complete questionnaire) and thus considered missing at random.

### MRI data acquisition

MRI was acquired on 3-Tesla MRI systems: a Siemens Verio/SkyraFit system (Edinburgh scanner 1), three Siemens Prisma systems (Glasgow, Dundee, Edinburgh scanner 2) and a Philips Achieva system (Aberdeen). For the current study, the T1W, T_2_-weighted (T2W) and 2D FLAIR images were selected for use (see Supplementary Table 1 and Meijboom *et al*.^24^ for imaging parameters).

### MRI data processing

Full details on brain data processing are described in Meijboom *et al*.^24^ and in brief below.

#### Neuroinflammation—qualitative assessment

2D FLAIR images were reviewed by a neuroradiologist for the presence of new/enlarging WMLs at follow-up versus baseline, with a binary outcome of yes/no. A random sample of 10% of participants was selected for intra- and inter-rater reproducibility. These were assessed by the same reviewer after at least four weeks, and separately by a second neuroradiologist. Consensus results were established.

#### Neuroinflammation—quantitative assessment

FLAIR images were registered to the T1W image with a rigid body transformation (degrees of freedom = 6) using FSL FLIRT (FSL6.0.1).^29,30^ Brain extraction was performed using FSL BET2 (FSL v6.0.1).^31^ The resulting baseline intracranial masks were edited using ITK-SNAP (v3.8.0),^32^ and volumes were extracted using fslstats (FSL v6.0.1). Hyperintense voxels on 2D FLAIR were identified by thresholding intensity values to t = (1.69 ∗ standard deviation) > mean, using an adjusted method from Zhan *et al*. ^33^ Resulting WML masks were manually edited using ITK-SNAP,^32^ and WML volumes were extracted using fslstats. WML volumes were divided by intracranial volume (ICV) to correct for variability in head size, and reported as ratio-ICV (r-ICV).

#### Neurodegeneration—brain

**Figure 1:**
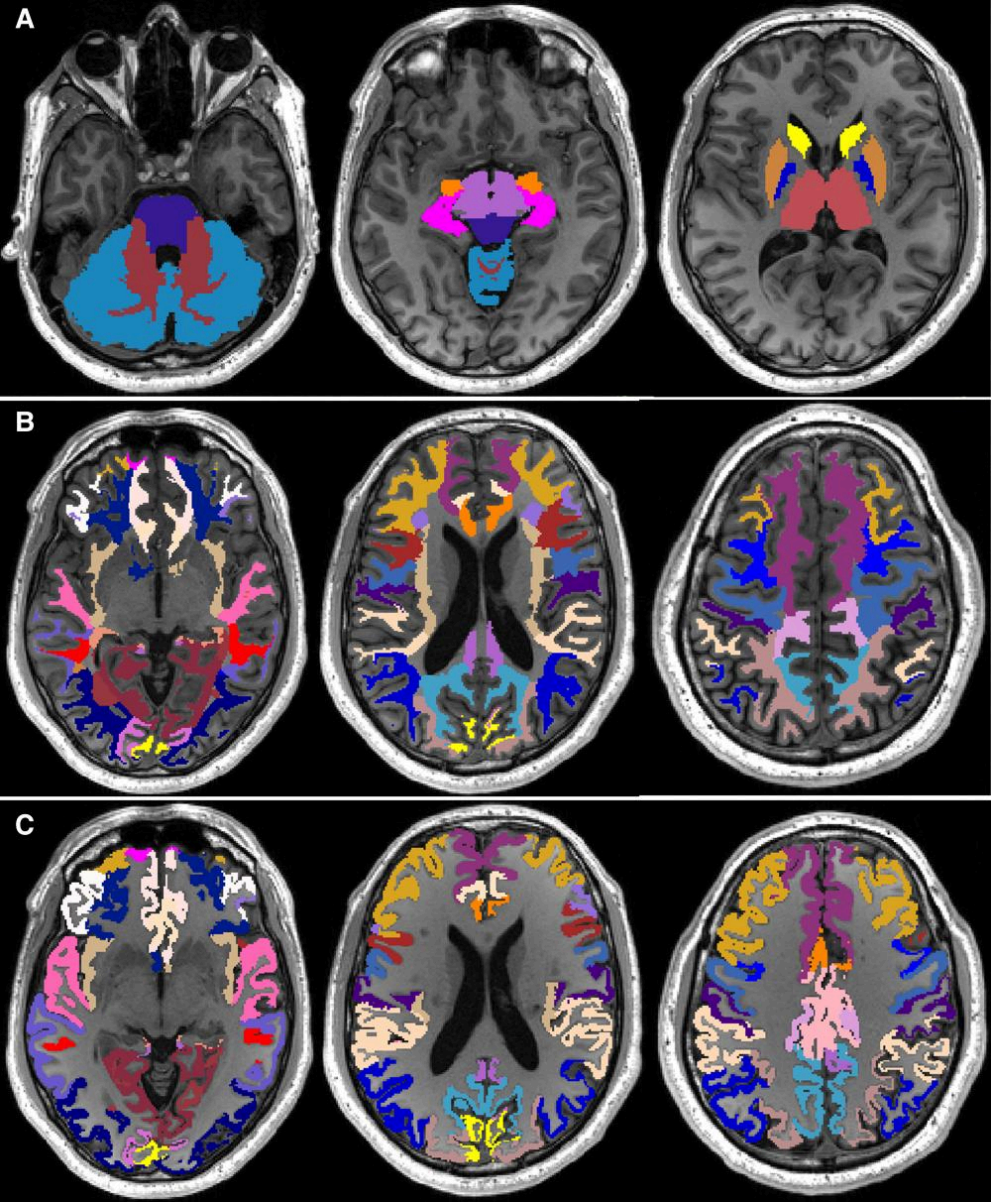
**Visual illustration of brain tissue regions used in statistical analysis.** (**A**) Subcortical, brainstem and cerebellar masks, (**B**) regional white matter masks and (**C**) regional grey matter masks. All masks are shown on an example subject’s axial T_1_-weighted image, with the sagittal T_1_-weighted image (right) indicating axial slice position. This figure was created using MRIcron (https://www.nitrc.org/projects/mricron).

Tissue segmentation was performed on T1W images using the Freesurfer (v6.0) longitudinal processing stream^34^ with default settings, and edited intracranial image as brain mask. Tissue segmentations were based on the Desikan-Killiany atlas,^35^ and pial surfaces were improved using additional contrast from T2W images. Lesion filling was not performed as previous literature has shown MS lesion filling did not change FreeSurfer output.^36^ Instead, output was manually edited where necessary using FreeView2.0 to ensure segmentation accuracy. WML masks were subtracted from final tissue segmentations (excluding whole-brain mask) using fslmaths (FSL6.0.1), to correct tissue volumes for WMLs. Bilateral regional cerebral WM and GM, cerebellar WM and GM, subcortical, as well as cerebral WM and GM and whole-brain volumes (see Table 2; Fig. 1) were then extracted using fslstats (FSL v6.0.1) and divided by ICV to correct for variability in head size (r-ICV).

**Table 2:** Brain tissue regions used in statistical analyses

**Whole-brain and cerebrum**	Whole-brain Cerebral GM Cerebral WM	**Brainstem and cerebellum**	Brainstem Cerebellar GM Cerebellar WM	**Subcortical regions**	Amygdala Caudate Hippocampus Nucleus accumbens Pallidum Putamen Thalamus Ventral diencephalon		
**Regional WM and GM**	*Frontal*	Parietal		*Temporal*		*Occipital*	
	Superior frontal	Superior parietal		Superior temporal		Lateral Occipital	Insula
	Caudal middle frontal	Inferior parietal		Middle temporal		Lingual	
	Rostral middle frontal	Supramarginal		Inferior temporal		Cuneus	
	Pars opercularis	Postcentral		Superior temporal sulcus banks		Pericalcarine	
	Pars triangularis	Precuneus		Fusiform			
	Pars orbitalis	Posterior cingulate		Transverse temporal			
	Lateral orbitofrontal	Isthmus cingulate		Entorhinal			
	Medial orbitofrontal			Temporal pole			
	Precentral			Parahippocampal			
	Paracentral						
	Frontal pole						
	Caudal anterior cingulate						
	Rostral anterior cingulate						

All individual regions were included bilaterally and in separate statistical models.

WM, white matter; GM, grey matter.

#### Neurodegeneration—spinal cord

**Figure 2:**
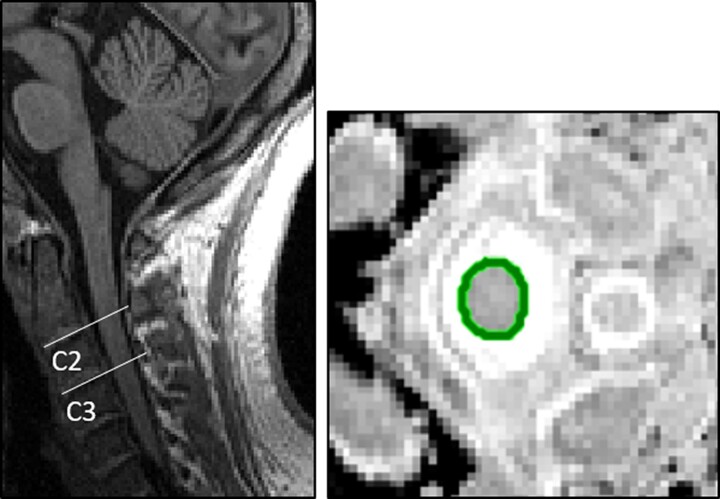
**Visual illustration of the spinal cord areas used in statistical analysis.** The spinal cord with cervical layers 2 (C2) and 3 (C3) highlighted is shown on the left, and cervical spinal cord cross-sectional area (SCCSA) highlighted in green is shown on the right.

Spinal cord cross-sectional area (SCCSA) was segmented from brain T1W images using an in-house developed software (previously described in Liu *et al*.^37^ and Mina *et al*.^38^). Spinal cord edges directly behind the vertebrae levels C1–C7 were selected. The algorithm then automatically reformatted axial images perpendicular to these edges and detected the cross-sectional area at each point along the cord edge using the Canny method.^39^ This was then plotted as the SCCSA profile plot, and average SCCSA (mm^2^) was calculated for each vertebral level. After visual inspection, scans were excluded if SCCSA could not be detected in more than 40% of axial reformatted planes along the cord. Upper cervical SCCSA for vertebrae levels 2 and 3 (Fig. 2) were then averaged to establish mean regional SCCSA C2–3.

### Statistical analyses

Statistical analyses were performed using R v4.0.2. Significance of demographic differences between fatigue groups was assessed using an independent two-sided *t*-test for continuous variables and chi-squared test for categorical variables (*P* < 0.05). Additionally, longitudinal change of FSS scores between time points was compared using a paired one-sided *t*-test (*P* < 0.05).

For the models described below, PHQ-9 was included as control variable to minimize the effect of the known link between depression and fatigue (multicollinearity was not observed). MRI scanner effect was also controlled for. False discovery rate correction (*q* < 0.05) was performed across all analyses within each model, for each model separately (e.g. all analyses in model ‘a’ below), to adjust for multiple comparisons.

#### Tissue/WML volumes and SCCSA C2–3

Fatigue group differences between tissue/WML volumes and SCCSA C2–3 at a) baseline, b) follow-up, and c) longitudinal change over 1-year were assessed as per below.

For brain tissue volumes, the following models were performed for each individual brain region separately:

- Linear regression model to assess fatigue group as predictor of brain volume ‘at baseline’, with PHQ-9_baseline_, age, sex, MRI scanner and WML_baseline_ as control variables;
- Linear regression model to assess fatigue group as predictor of brain volume ‘at follow-up’, with PHQ-9_baseline_, age, sex, MRI scanner, WML_follow-up_ and DMT_follow-up_ as control variables; and
- Mixed-model regression to assess fatigue group, time point and their interaction as predictors of brain volume, with PHQ-9, age, sex, MRI scanner, WML and DMT_follow-up_ as control variables, to assess effect of fatigue on change over time.

For whole-brain volume, WML volume and SCCSA C2–3, the above models were repeated, but excluding WML volume as covariate. Additionally, after mixed-model regression analysis, two *post hoc* linear regression models were performed to assess whole-brain volume, WML volume and SCCSA C2–3 change within fatigue groups separately; control variables were identical.

#### New/enlarging WMLs

Binomial logistic regression was performed to assess associations between baseline fatigue groups and new/enlarging lesions (yes/no) at follow-up, corrected for control variables PHQ-9_baseline_, age, sex, MRI scanner and DMT_follow-up_. An exploratory binomial logistic regression analysis was performed to assess associations between new/enlarging lesions and fatigue groups based on follow-up FSS scores.

## Results

### Data availability

Data for *n* = 354 participants were available. For brain and spinal cord quantitative measures, *n* = 39 and *n* = 27, respectively, failed data processing quality checks; *n* = 2 and *n* = 3, respectively, had missing DMT data; and were excluded. FSS and PHQ-9 scores were imputed for small numbers of participants (FSS 8 at baseline, 5 at follow-up; PHQ-9 6 at baseline, 2 at follow-up).

### Demographics and clinical variables

Mean follow-up time was 387 days (SD 40.6 days). FSS score did not change significantly over time (t(312) = 0.16, *P* = 0.87). Sex (*X*^2^(1, 313) = 0.01; *P* = 0.92) and age (t(311) = −1.64, *P* = 0.10) were not significantly different between fatigue groups. Fatigued participants had a higher EDSS and PHQ-9 score than non-fatigued participants, at both baseline (t_EDSS_(311) = −6.81, *P* < 0.001; t_PHQ_(311) = −10.17, *P* < 0.001) and follow-up (t_EDSS_(311) = −6.98, *P* < 0.001; t_PHQ_(311) = −6.94, *P* < 0.001). Additionally, a significantly higher number (80.9%) of fatigued participants were receiving DMT at follow-up (*X*^2^(1, 313) = 7.51; *P* < 0.01), and had a clinical relapse (26.1%) between baseline and follow-up (*X*^2^(1, 313) = 6.40; *P* < 0.05), than in the non-fatigued group (DMT: 67.3%; clinical relapse: 14.0%). Despite this significant difference, the majority (73.9%) of participants with fatigue did not have a clinical relapse between baseline and follow-up. In total, 40% of participants with fatigue did not have a clinical or radiological relapse between baseline and follow-up (see Supplementary Fig. 1). These results were identical for the spinal cord sample; see Supplementary Section 1.

Six participants received steroid treatment within six weeks prior to the baseline MRI and two within six weeks prior to follow-up MRI. Only two participants were on stimulant drugs for fatigue. Specifically, one participant started amantadine treatment between baseline and follow-up, and one participant received amantadine at both time points. Modafinil, armodafinil, methylphenidate or dextroamphetamine was not prescribed to any study participants. See Table 3 for participant demographics and clinical variables and Supplementary Table 2 for details on DMTs prescribed at follow-up.

**Table 3:** Participant demographics and clinical variables

	Participants where brain data were available (*n* = 313)		Participants where SCCSA C2–3 data were available (*n* = 324)	
	Fatigued	Non-fatigued	Fatigued	Non-fatigued
*n* (F)	142 (107)	171 (128)	149 (114)	175 (130)
Mean age in years at w0 (SD)	39.1 (10.5)	37.3 (9.9)	39.2 (10.5)	37.8 (10.2)
Time since diagnosis (days) (mean, SD)	65.2 (40.2)	62.8 (38.4)	64.4 (39.2)	62.8 (38.0)
Number of cases with clinical relapses between w0 and w1 (%)	37 (26.1%)	24 (14.0%)	39 (26.2%)	26 (14.9%)
Median EDSS at w0 (IQR)	2.5 (1.5)	2.0 (1.0)	2.5 (1.5)	2.0 (1.0)
Median EDSS at w1 (IQR)	3.0 (1.5)	2.0 (1.0)	3.0 (2.0)	2.0 (1.5)
Mean FSS at w0 (SD)	48.3 (7.6)	23.2 (8.5)	49.0 (7.8)	22.9 (8.5)
Mean FSS at w1 (SD)	42.1 (15.0)	28.2 (14.9)	42.5 (15.0)	28.6 (14.8)
Mean PHQ-9 at w0 (SD)	10.7 (6.2)	4.8 (3.9)	11.2 (6.5)	4.9 (4.0)
Mean PHQ-9 at w1 (SD)	8.1 (6.2)	4.0 (4.3)	8.4 (6.3)	4.3 (4.4)
Number of cases with DMT at w1 (%)	115 (80.9%)	115 (67.3%)	121 (81.2%)	124 (70.9%)
Mean WB volume (r-ICV) at w0 (SD)	0.739 (0.040)	0.743 (0.036)		
Mean WB volume (r-ICV) at w1 (SD)	0.734 (0.041)	0.740 (0.035)		
Mean WB volume change (r-ICV) (w1–w0)	−0.60%	−0.43%		
Mean WML volume (r-ICV) at w0 (SD)	0.009 (0.008)	0.008 (0.006)		
Mean WML volume (r-ICV) at w1 (SD)	0.010 (0.007)	0.010 (0.007)		
Mean WML volume change (r-ICV) (w1–w0)	19.13%	24.09%		
Number of cases with new/enlarging WML at w1 (%)	69 (48.6%)	87 (50.9%)		
Mean SCCSA at w0 (SD)			64.21 (8.13)	65.34 (7.18)
Mean SCCSA at w1 (SD)			62.58 (7.92)	63.03 (7.40)
Mean SCCSA change (w1–w0)			−2.54%	−3.53%

*n*, sample size; F, female; w0, baseline; w1, 1-year follow-up; SD, standard deviation; IQR, interquartile range; EDSS, expanded-disability status scale; DMT, disease-modifying treatment; FSS, Fatigue Severity Scale; PHQ-9, Patient Health Questionnaire; SCCSA, spinal cord cross-sectional area; C2–3, cervical regions 2 and 3; WB, whole-brain; WML, white matter lesion; r-ICV, ratio intracranial volume.

### Neurodegeneration and fatigue

**Figure 3:**
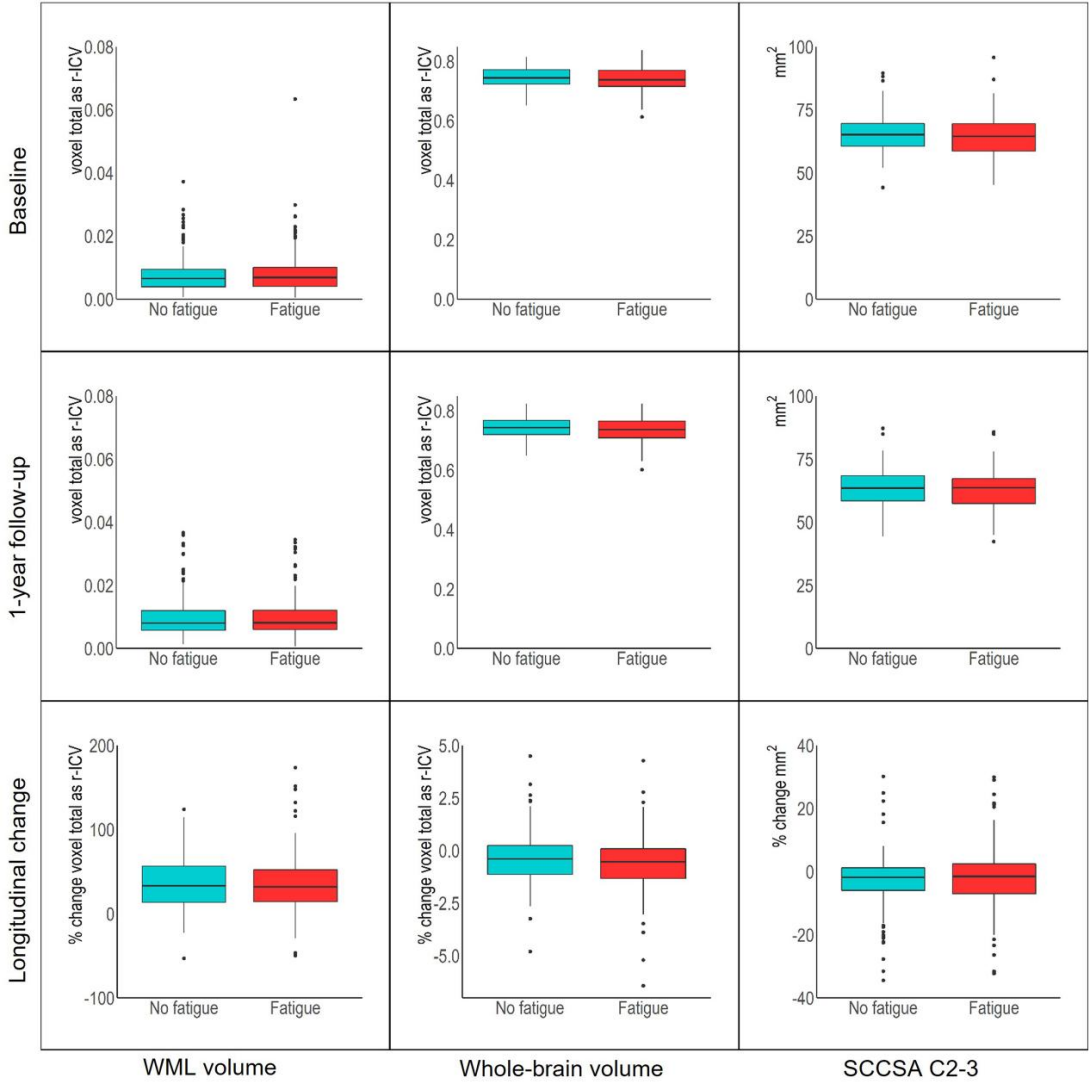
**Brain and spinal cord structural MRI findings in fatigue groups.** Mean whole-brain volume, white matter lesion (WML) volume and cervical spinal cord cross-sectional area 2–3 [mm^2^ (SCCSA C2–3)] at baseline, 1-year follow-up and % change over time (w1–w0) for fatigue [Fatigue Severity Scale (FSS) score ≥ 36] and no-fatigue groups (FSS < 36). Whole-brain and WML volumes are expressed as total voxel count as ratio of intracranial volume (r-ICV).

**Figure 4:**
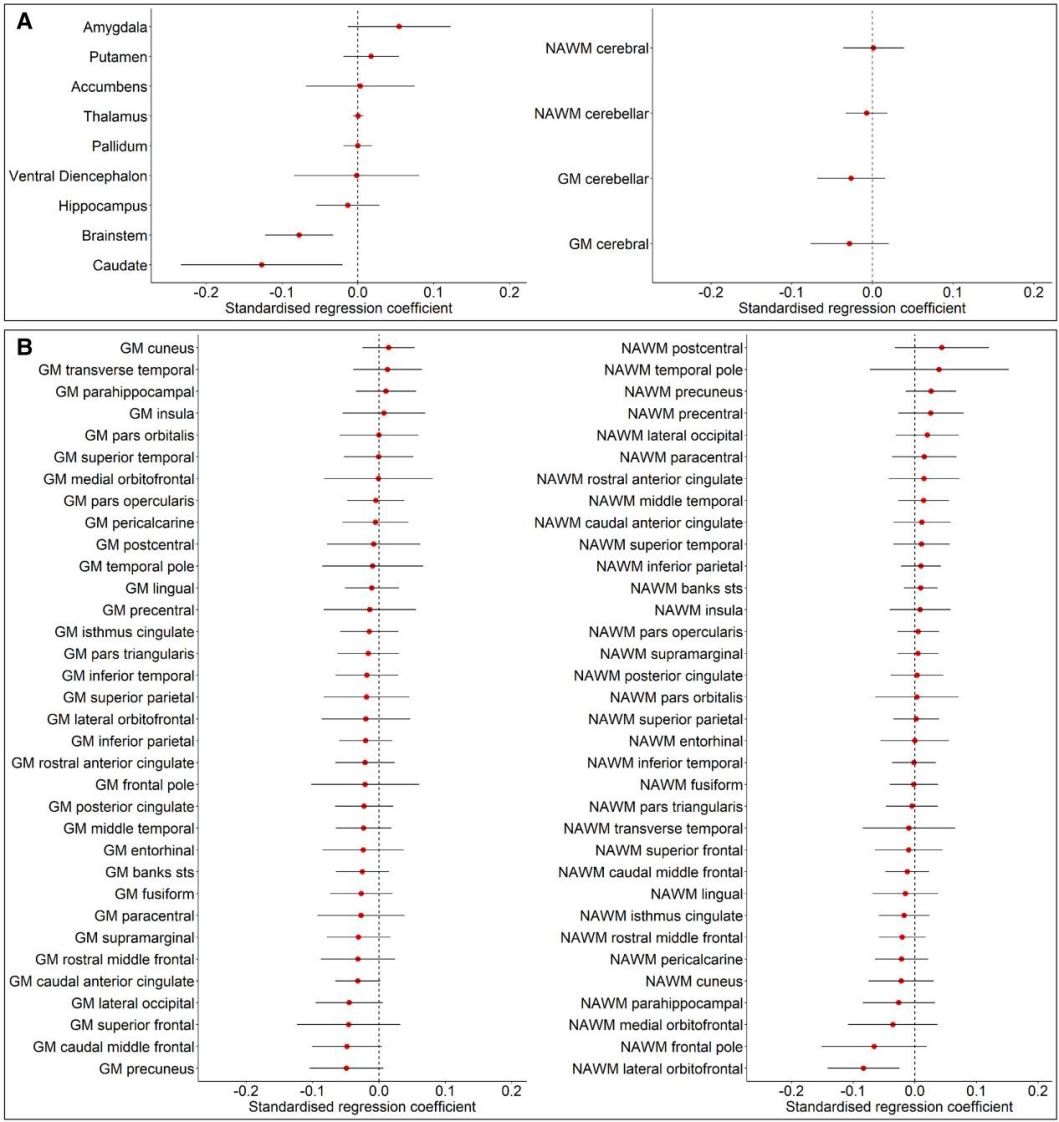
**Regional brain structural MRI findings in fatigue groups.** Non-significant standardized (*Z*-scores) regression coefficients of the interaction effect of fatigue group and time (circles) on (**A**) subcortical regions, and cerebellar and cerebral normal-appearing white (NAWM) and grey matter (GM), and (**B**) for regional brain NAWM and GM. Standardized regression coefficients < 0 indicate a negative effect of time and fatigue on brain tissue volume, i.e. atrophy; standardized regression coefficients > 0 indicate a positive effect of time and fatigue on brain atrophy volume, i.e. volume increase. Lines indicate 95% confidence intervals for each regression coefficient. Regression coefficients were not significant after corrections for multiple comparisons (false discovery rate corrected; *q* > 0.05).

Baseline, follow-up and longitudinal change in brain tissue volumes and regional SCCSA C2–3 were not significantly associated (*q* > 0.05) with fatigue status in people with RRMS [(pwRRMS); Supplementary Tables 3–6; Figs 3 and 4].

*Post hoc* tests showed that whole-brain volume and SCCSA C2–3 significantly changed over time in each fatigue group separately (*q* < 0.05; Supplementary Table 7). In the non-fatigued group, the control variables of scanner and age were significant for whole-brain volume and SCCSA C2–3 (*P* < 0.05). In the fatigued group, sex, DMT and age were significant for whole-brain volume, and scanner was significant for SCCSA C2–3 (*P* < 0.05).

### Neuroinflammation and fatigue

**Figure 5:**
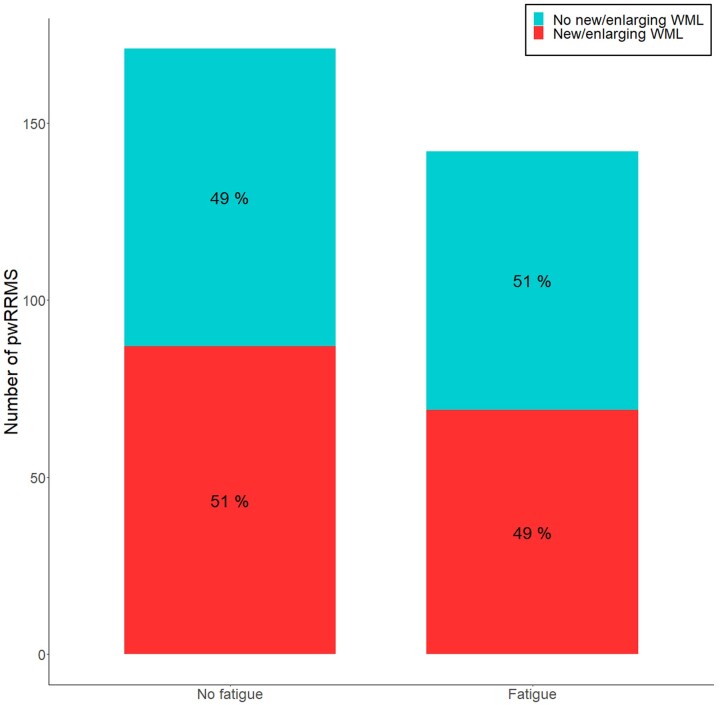
**Neuroinflammatory relapse in fatigue groups.** Percentage of people with relapsing–remitting multiple sclerosis (pwRRMS) without (left) or with (right) fatigue as per baseline Fatigue Severity Scale score (<36, ≥36, respectively); with (red) or without (blue) new/enlarging white matter lesions (WML) over 1 year.

The presence of new/enlarging WMLs at follow-up was not significantly associated with baseline fatigue status in pwRRMS [87/171 (51%) non-fatigued versus 69/142 (49%) fatigued, OR = 0.72, *P* = 0.25; Fig. 5 and Supplementary Table 6]. This was also observed for follow-up fatigue status [83/171 (51%) non-fatigued versus 73/142 (49%) fatigued, OR = 0.93, *P* = 0.80].

Baseline, follow-up and longitudinal change in WML volume were not significantly associated (*q* > 0.05) with fatigue status (Supplementary Tables 3–6, Fig. 3). *Post hoc* tests showed that WML volume significantly changed over time in each fatigue group separately (*q* < 0.05; Supplementary Table 7). In the non-fatigued group, control variable DMT was significant (*P* < 0.05); and in the fatigued group, none of the control variables were significant (*P* > 0.05).

## Discussion

This study aimed to identify neuroinflammatory and regional neurodegenerative substrates of fatigue in early RRMS, by exploring cross-sectional and longitudinal associations with brain and upper cervical spinal cord MRI. Counter to our initial hypothesis, we found no differences in CNS MRI measures between fatigued and not significantly fatigued groups in our large cohort of people with recently diagnosed RRMS. This suggests that fatigue-associated pathophysiology is not reflected in structural MRI measures of neuroinflammation (WMLs) or neurodegeneration (atrophy) in early RRMS. This novel negative finding in a large multi-centre cohort of people with recently diagnosed RRMS helps to resolve mixed findings in the existing literature, and emphasizes the need to apply more sensitive and specific imaging methods to investigate fatigue in RRMS. Our finding that fatigue is also prevalent without inflammatory relapse is also of practical significance for clinical management of RRMS, in that it identifies potential ‘hidden disability’ in those with apparently inactive disease who may not therefore be receiving optimal treatment.

### Neuroinflammation and fatigue

WML volume and the presence of new/enlarging WMLs were not associated with fatigue in our recently diagnosed cohort. Nourbakhsh *et al*.^14^ also found no association in an early MS cohort, using the Modified Fatigue Impact Scale (MFIS). Findings at later disease stages are mixed: Gilio *et al*.^40^ observed an association between fatigue and T2 lesion load in RRMS, whilst Novo *et al*.^41^ found no association; both used the MFIS. Positive associations between T2 lesion load and fatigue measured with different assessment scales have also, however, been reported.^42^ WML volume at a given time point may reflect historical cumulative neuroinflammatory damage, and is known to show limited correlation with clinical disability.^43^ The absence of any demonstrable relationship in early disease, and variable results in later disease, indicate at most a weak relationship between the degree of established neuroinflammatory damage visible as WMLs and fatigue. This is further supported by previous literature showing a lack of association between DMTs targeting inflammation and a decrease in fatigue.^44^

The absence of any association between fatigue and new or enlarging brain WMLs at visual inspection suggests that interval neuroinflammatory activity manifesting as radiological relapse may not be a determinant of fatigue. We were unable, however, to measure interval neuroinflammatory activity in the spinal cord, which means that the effect of radiological spinal cord relapse on fatigue remains unclear and requires further investigation. Furthermore, pwRRMS with fatigue experienced a significantly higher number of clinical relapses, although prevalence of clinical relapse was relatively low in both fatigue (26%) and non-fatigue (14%) groups. Notably, 40% of fatigued pwRRMS did not experience either a radiological or a clinical relapse (Supplementary Fig. 1). Overall, this indicates that fatigue commonly occurs without radiological and clinical relapses; this may be seen as analogous to the increasingly recognized phenomenon of disability progression in the absence of relapse activity (PIRA),^45^ although the mechanisms underlying fatigue and disability progression may differ. Fatigue may therefore be similarly under-recognized in those who are thought to have inactive disease.

### Neurodegeneration and fatigue

We found no significant association between fatigue and either baseline or 1-year follow-up volume, and atrophy measures in any brain region. A total whole-brain volume decrease of 0.50% for this overall cohort has been reported previously^46^; the current analysis showed a decrease of 0.60% for fatigue and 0.43% for the non-fatigue group, which were not statistically significantly different. These changes exceed the annual 0.40% threshold for normal atrophy suggested by MAGNIMS.^12^ Therefore, although disease-related atrophy is present, it does not appear to be a significant driver of fatigue in early RRMS. It is, however, possible that such a relationship may be apparent over longer intervals. Of note, caudate and brainstem atrophy rates were significantly different between fatigue and non-fatigued groups prior to correction for multiple comparisons. Previous research has linked both regions, especially the caudate, to fatigue in MS.^15,20^ Results from the current study highlight them as potential regions of interest for further longitudinal study, and particularly for studies exploring more sensitive MRI markers of neurodegeneration.

The absence of associations between atrophy and fatigue observed here contrasts with the limited previous literature on structural brain MRI and fatigue in early RRMS. Previous studies observed associations between fatigue and cerebellar,^13^ thalamic,^14^ basal ganglia^15^ and pontine^15^ volumes, and regional cortical surface area.^16^ The difference between findings may be due to different methodology, for example use of different analysis software, a range of sample sizes and varying definitions of early disease. There are very limited published data in patient groups directly comparable to our study. A recent study by Fleischer *et al*.^15^ in a large treatment-naïve, early-stage RRMS cohort employed a broadly similar design, but observed cross-sectional associations of caudate, putamen, pallidum, thalamus and pons volumes with fatigue, as well as with fatigue at 4-year follow-up. The different results between our studies may in part be due to the use of different fatigue scales. Of note, Fleischer *et al*.^15^ used two different fatigue scales [MS inventory of cognition (MUSIC) and fatigue scale for motor and cognitive functions (FSMC)], and observed different associations with brain regions for these scales. This emphasizes the limitations of, and variability between, different fatigue scales for measuring fatigue accurately and consistently.

Similarly, we did not find an association between fatigue and upper cervical spinal cord area change in early RRMS, although there was a significant decrease of upper spinal cord area in both fatigue (2.54%) and non-fatigued (3.53%) groups. This suggests that although there may be spinal cord atrophy in both fatigue groups, it is unlikely to be a significant driver of fatigue. Existing literature is again very limited, however this negative finding accords with the majority of previous studies that also reported no such association.^21,22^ One study did report a specific correlation between upper cervical cord area and self-reported motor fatigue.^47^ Neurodegenerative changes throughout the spinal cord are recognized in MS,^48^ and links between fatigue and motor impairment are thought to be related to spinal cord damage.^49^ More comprehensive analysis of atrophy along the full length of the spinal cord may be needed to capture neurodegeneration underlying fatigue.

Atrophy measures may be derived from widely available structural MRI and are considered a clinically applicable surrogate marker of neurodegeneration.^12^ It is important to note, however, that tissue volume change is an indirect downstream measure, which has limited sensitivity and specificity for neuronal and axonal loss, and may be confounded by other processes such as changes in hydration state and pseudoatrophy from resolution of transient inflammation.

### Mechanisms of fatigue in RRMS

The negative findings of this study based on quantitative analysis of structural brain MRI in a substantial early RRMS cohort beg the question as to what does indeed cause fatigue in RRMS. A number of potential MS-related pathophysiological mechanisms may drive fatigue, including loss of myelin and axonal integrity leading to impaired neuronal conduction, mitochondrial dysfunction interfering with normal neuronal function and subtle or diffuse low-grade inflammation.^50^

Functional, microstructural, metabolic and molecular imaging approaches show potential for exploring disease mechanisms underlying fatigue *in vivo*.^51^ fMRI may reveal changes in functional networks^52^ and provide an objective biomarker of fatigue, although it is less specific to pathophysiological mechanisms leading to those changes. Microstructural MRI techniques such as diffusion^53^ and magnetization transfer MRI^54^ can detect changes at the axonal and myelin level. Blood biomarkers such as plasma neurofilament light chain provide markers of central neurodegeneration.^55^ Metabolic insights from spectroscopic techniques, sodium imaging and PET can probe regional energetic status. Paramagnetic rim lesions due to iron-laden macrophages detected with susceptibility-weighted imaging or quantitative susceptibility maps are thought to provide surrogate indicators of low grade or smouldering inflammation,^56^ and 18 kD human translocator protein (TSPO) PET is highly sensitive to global and focal neuroinflammation.^57^ Additionally, the relationship between neuroinflammatory lesion distribution and fatigue may also be relevant; this has been previously explored with other symptoms.^58,59^

### Limitations

Accurate fatigue assessment is difficult due to its multidimensional nature. In the FutureMS study, the FSS was chosen as a single measure of generic fatigue, due to its validation in MS and wide use.^25^ The FSS, however, does not explore different domains of fatigue, and is non-linear in nature; we therefore dichotomized scores into ‘fatigued’ and ‘non-fatigued’ groups, based on a previously reported threshold value.^27^ Nonetheless, current methods of fatigue assessment remain a significant limitation in all studies of this type.

The RRMS cohort included in this study was relatively homogenous, based on time of RRMS diagnosis as an objective measure of disease duration; we acknowledge that symptom duration prior to diagnosis will be variable for pwMS, however no accurate measure is available for this.

Healthy control data were not available. The aim of this study was, however, to investigate changes in neurodegeneration and neuroinflammation related to fatigue in RRMS, rather than assessing whether these changes were different from healthy people.

MRI for the multi-centre FutureMS study was acquired across five systems. The use of different MR systems may bias results. Data pooling across sites allows larger cohort numbers, however, and is key for clinical studies, as it improves external validity. Importantly, all participants underwent baseline and follow-up MRI on the same system with the same protocol, in order to reduce bias. Furthermore, MRI system was included as control variable in the statistical models.

Pseudoatrophy, an apparent decrease in volume not caused by actual tissue loss, is attributed to resolving inflammation; it may be associated with DMT and can also occur spontaneously. This phenomenon is particularly notable in the WM, primarily impacted by inflammation, and less pronounced in the GM.^12^ Since a relationship between GM volume and fatigue was not observed, which is consistent with results for all the other examined regions, the influence of pseudoatrophy on the results is likely to be minimal. Furthermore, we have corrected for the effect of MS treatment on atrophy, by including DMT as a control variable.

The FutureMS imaging protocol did not include dedicated spinal cord imaging, which limited spinal cord evaluation to upper cervical spinal cord cross-sectional area measures from a volumetrically acquired T1W brain sequence. Nonetheless, given the limited existing literature, we believe that these upper cervical atrophy data in a well-characterized early RRMS cohort contribute significant novel information. Further studies including dedicated whole spinal cord imaging are required to establish what role spinal cord degeneration and inflammation plays in fatigue.

## Conclusions

Our results suggest that fatigue in early RRMS is not driven by neuroinflammation or neurodegeneration measured with current structural MRI. These novel negative findings in a large multi-centre cohort of people with recently diagnosed RRMS help to resolve uncertainty in existing literature. Furthermore, we showed that fatigue is prevalent in people without radiological relapse in the brain, who are not considered to have active disease. This suggests that symptom detection and treatment should remain a clinical priority regardless of neuroinflammatory disease activity. Our findings emphasize the need for applying more sensitive and specific biomarkers of neurodegeneration, neuronal function and neuroinflammation in the brain and spinal cord to elucidate the mechanisms underlying fatigue in RRMS; this will ultimately aid development of more effective future targeted treatments.

## Supplementary Material

fcae278_Supplementary_Data

## Data Availability

The FutureMS project agreement, under reference LG/CPH/UOF001.2011, between The University Courts of the University of Glasgow, Edinburgh and Aberdeen, as well as the Grampian, Tayside, Lothian and Greater Glasgow Health boards, states that personal data cannot be released or made available to any third party unless the FutureMS steering committee have expressly permitted the data sharing, or personal data have been anonymized sufficiently. The magnetic resonance imaging data used in the current paper are not defaced and hence not sufficiently anonymized for open data sharing. Unrestricted access to these images in current form would present a confidentiality/privacy risk for participants. Additionally, the informed consent, approved by the South East Scotland Research Ethics Committee 02 under reference 15/SS/0233, states that by consenting to the FutureMS study, participants agree to share their data with the wider study team. The authors have not obtained explicit consent for deidentified data sharing beyond the wider study team. As such, they cannot make deidentified data openly available. The authors do, however, highly encourage collaboration with other research teams, and any researchers may request access to anonymized patient data from FutureMS-1 by contacting future-ms@ed.ac.uk. Proposals will be reviewed by the FutureMS steering committee and if approved, a signed data sharing agreement will be issued. Statistical analysis code generated and used in the current study can be downloaded from https://git.ecdf.ed.ac.uk/rmeijboo/statistical-analysis.

## Appendix I

### Members of the FutureMS consortium

The FutureMS consortium includes clinicians, data analysts or other FutureMS team members that have contributed to acquisition of data, access to patients or whom have been relied upon for their input.

Amit Akula, Sergio Baranzini, Fiona Barret, Mark Bastin, Chris Batchelor, Emily Beswick, Fraser Brown, Tracy Brunton, Javier Carod Artal, Siddharthan Chandran, Jessie Chang, Yingdi Chen, Shuna Colville, Peter Connick, Annette Cooper, Denise Cranley, Rachel Dakin, Baljean Dhillon, Elizabeth Elliott, Peter Foley, James Finlayson, Stella Glasmacher, Angus Grossart, Haane Haagenrud, Katarzyna Hafezi, Emily Harrison, Adil Harroud, Sara Hathorn, Michael Hendry, Samantha Hendry, Justine Hudson, David Hunt, Tracey Hopkins, Aidan Hutchison, Baljit Jagpal, Charlotte Jardine, Kiran Jayprakash, Matt Justin, Agniete Kampaite, Patrick Kearns, Gwen Kennedy, Lucy Kessler, Michaela Kleynhans, Juan Larraz, Katherine Love, Dawn Lyle, James MacDonald, Niall MacDougall, Jen MacFarlane, Lesley Macfarlane, Alan Maclean, Bev MacLennan, Margaret-Ann MacLeod, Nicola MacLeod, Don Mahad, Paula Marshall, Sarah-Jane Martin, Conni McCarthy, Rozanna Meijboom, Daisy Mollison, Ian Megson, Mary Monaghan, Lee Murphy, Judith Newton, Jonathan O’Riordan, David Perry, Claire Purdie, Suzanne Quigley, Laura-Jayne Queripel, Adam Scotson, Scott Semple, Amy Stenson, Michaela Stuart, Thomas Suslak, Michael Thrippleton, Gordon Waiter, Adam Waldman, Christine Weaver, Stuart Webb, Belinda Weller, Anna Williams, Isabelle Williams, Stewart Wiseman, Nicole White, Charis Wong, Michael Wong, Rosie Woodward and Elizabeth York.
