# Supplementary material for: Fatigue in early multiple sclerosis: MRI metrics of neuroinflammation, relapse and neurodegeneration
Source: Brain Commun. 2024 Aug 14;6(5):fcae278. doi: 10.1093/braincomms/fcae278 (PMC11462441; doi:10.1093/braincomms/fcae278)
Supplement: fcae278_Supplementary_Data [file fcae278_supplementary_data.pdf]

SUPPLEMENT

Supplementary Section 1. Demographic comparisons for the spinal cord sample

FSS score did not change significantly over time (t(323) = -0.084, p=0.93). Sex (X^2(1, 324) = 0.19; p=0.67) and age (t(322)=-1.21, p=0.23) were not significantly different between fatigue groups. Fatigued participants had a higher EDSS and PHQ-9 score than non-fatigued participants, at both baseline (t_{EDSS}(322)=-6.62, p<0.0001; t_{PHQ}(322)=-10.73, p<0.0001) and follow-up (t_{EDSS}(322)=-6.69, p<0.0001; t_{PHQ}(322)=-6.92 , p<0.0001). Additionally, a significant higher number (81.2%) of fatigued participants were receiving DMT at follow-up (X^2(1, 324)=4.68; p<0.05), and had a clinical relapse (26.2%) between baseline and follow-up (X^2(1, 324)=6.10; p<0.05), than in the non-fatigued group (DMT: 70.9%; clinical relapse: 14.9%). Despite this significant difference, the majority (73.8%) of participants with fatigue did not have a clinical relapse between baseline and follow-up.

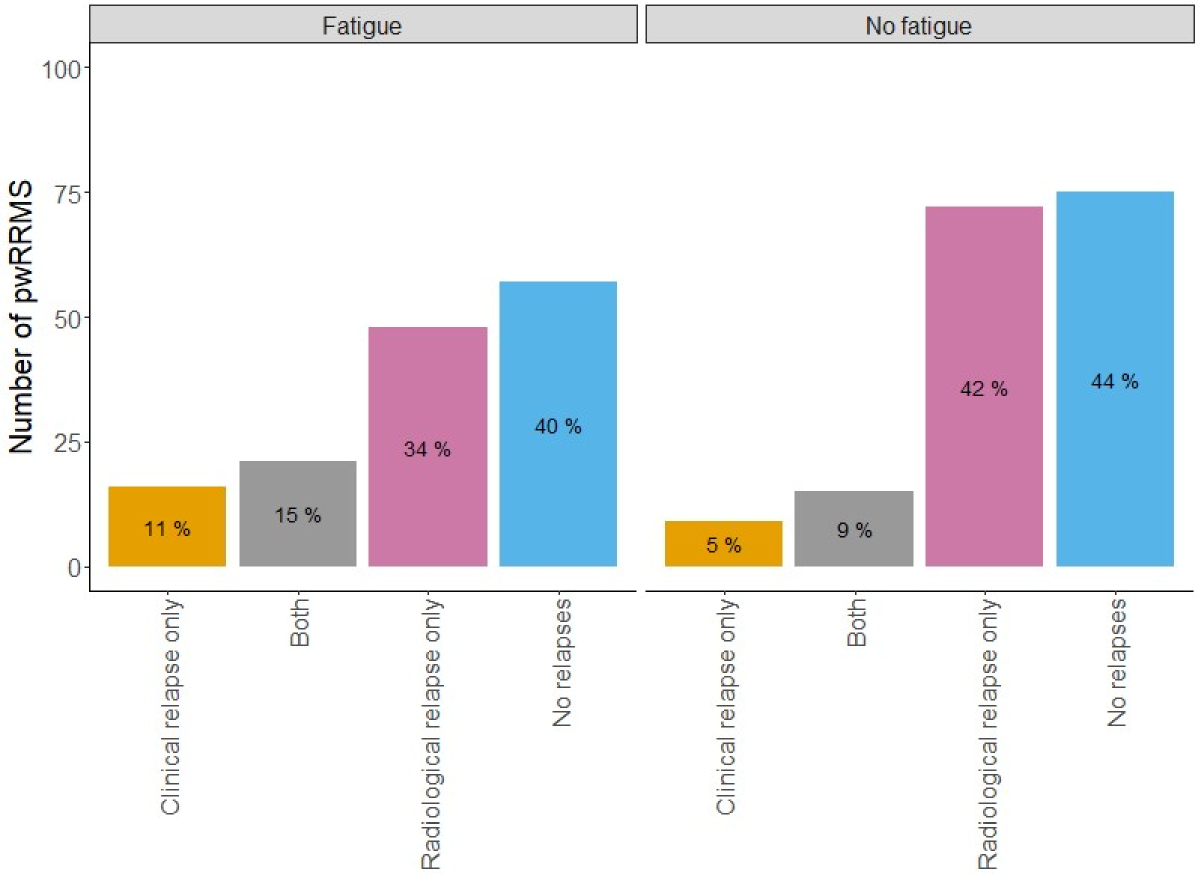

Supplementary Figure 1. Radiological and clinical relapse distribution across fatigue (left) and non-fatigued (right) people with relapsing-remitting multiple sclerosis (pwRRMS). Fatigue status was established using baseline Fatigue Severity Scale (FSS) score (fatigued: ≥36; non-fatigued: <36). Participant with 'clinical relapses only' are shown in yellow, 'radiological relapses only' are shown in grey, and 'both relapse types' are shown in green. Participants without clinical or radiological relapses are shown in blue. This figure is based on the brain data sample.

Supplementary Table 1. Overview of FutureMS MRI parameters of sequences included in the current paper.

PROTOCOL A[1]												
**Sequence**	**T1-weighted**				**T2-weighted**				**2D FLAIR**			
	**EDI1**	**GLA**	**DUN**	**ABN**	**EDI1**	**GLA**	**DUN**	**ABN**	**EDI1**	**GLA**	**DUN**	**ABN**
**Mode**	3D	3D	3D	3D	2D	2D	2D	3D	2D	2D	2D	2D
**FOV (mm)**	256	256	256	240	220	220	220	256	250	250	250	250
**Orientation**	Sag	Sag	Sag	Sag	Ax	Ax	Ax	Sag	Ax	Ax	Ax	Ax
**TR (ms)**	2530	2500	2500	3000	6000	6160	6160	2500	9500	9500	9500	11000
**TE (ms)**	3.37	2.26	2.26	3.9	96	96	96	310	124	124	124	125
**TI (ms)**	1100	1100	1100	1048	-	-	-	-	2400	2400	2400	2800
**Flip angle (deg)**	7	7	7	8	150	150	150	-	150	150	150	120
**Gap (mm)**	-	-	-	-	1.2	1.2	1.2	-	0	0	0	1
**Matrix (mm)**	256 × 256	256 × 256	256 × 256	240 × 240	320 × 320	320 × 314	314 x 314	256 x 256	256 × 256	256 × 256	256 x 256	252 x 226
**Voxel size (mm)**	1 × 1 × 1	1 × 1 × 1	1 × 1 × 1	1 x 1 x 1	0.7 × 0.7 × 4	0.7 × 0.7 × 4	0.7 × 0.7 × 4	1 x 1 x 1	1 × 1 × 3	1 × 1 × 3	1 x 1 x 3	1 x 1.1 x 3
**Slices reconstructed**	176	176	176	160	33	33	33	176	60	60	60	29
**Acq. Time (m: ss)**	6:03	5:59	5:59	5:38	1:26	1:03	1:03	3:42	7:38	7:38	7:38	5:08

PROTOCOL B[2]			
**Sequence**	**T1-weighted (MPRAGE)**	**T2-weighted dual echo (FSE)**	**2D FLAIR (PROPELLER)**
**Mode**	3D	2D	2D
**FOV (mm)**	256	250	250
**Orientation**	Sagittal	Axial	Axial
**TR (ms)**	2500	3630	9500
**TE (ms)**	2.26	9.6, 96	120
**TI (ms)**	1100	-	2400
**Flip angle (deg)**	7	150	150
**Gap (mm)**	-	0	0
**Matrix (mm)**	256 x 256	384 x 384	256 x 256
**Voxel size (mm)**	1 x 1 x1	0.7 x 0.7 x 3	1 x 1 x 3
**Slices**	176	60	60
**Acceleration factor (in-plane × slice)**	2 × 1	3 × 1	2 × 1
**Acq. Time (m:ss)**	5:59	4:01	4:47

FLAIR = fluid attenuated inversion recovery; EDI1 = Edinburgh scanner 1; ED2 = Edinburgh scanner 2; GLA = Glasgow; DUN = Dundee, ABN = Aberdeen, FOV = field of view; TR = repetition time; TE = echo time; TI = inversion time; deg = degree; acq. = acquisition; Sag = sagittal; Ax = axial

[1]Protocol A was used at EDI1, GLA, DUN and ABN

[2]Protocol B was used at EDI2, GLA, DUN and ABN

Supplementary Table 2. Number of people with RRMS who received disease-modifying treatment (DMT) after the baseline visit, including type(s) of DMT received, for fatigued and non-fatigue participants. DMT numbers and types are reported separately for brain and spinal cord samples. All participants were DMT-naïve at baseline.

	**Participants where brain volumetric data were available**		**Participants where spinal cord data were available**	
	**Fatigued**	**Non-Fatigued**	**Fatigued**	**Non-Fatigued**
Number of cases with DMT at 1-year follow-up (%)	115 (80.99%)	115 (67.25%)	121 (81.21%)	124 (70.86%)
Dimethyl fumarate	47	50	48	53
Glatirimer acetate	14	17	15	20
Alemtuzumab	9	13	10	13
Beta interferon	8	8	10	9
Teriflunomide	5	0	3	3
Fingolimod	2	5	1	5
Natalizumab	2	6	2	4
Azathioprine	1	0	1	0
Beta interferon & Glatirimer acetate	0	1	0	1
Beta interferon & Teriflunomide	1	0	1	0
Dimethyl fumarate & Alemtuzumab	0	1	0	1
Dimethyl fumarate & Azathioprine	1	0	1	0
Dimethyl fumarate & Beta interferon	1	2	1	2
Dimethyl fumarate & Fingolimod	0	1	0	1
Dimethyl fumarate & Glatirimer acetate	2	1	2	1
Dimethyl fumarate & Natalizumab	2	0	3	0
Dimethyl fumarate & Teriflunomide	1	1	1	1
Glatirimer acetate & Fingolimod	1	0	1	0
Other	15	6	14	7
Unknown	3	3	7	3

Supplementary Table 3. Results for baseline brain tissue volumes, WML volumes and SCCSA-C2-3 differences between RRMS participants with and without fatigue (based on FSS score). This was assessed using multiple linear regression models, with fatigue group as regressor of interest and MRI scanner, age_{w0}, sex, WML_{w0} (not for WML, whole-brain and SCCSA) and depression score (based on PHQ-9) as control variables. P-values for the regressor of interest were corrected for multiple comparisons using the FDR (q<.05).

		$B_{standardised}$	SD	t-value	$p_{uncorrected}$	p_{FDR}	CI 2.5%	CI 97.5%
Brainstem	Intercept	-0.1853	0.2627	-0.7054	0.4811		-0.7022	0.3316
	Fatigue group: fatigued	0.1126	0.1241	0.9078	0.3647	0.8738	-0.1315	0.3568
	Scanner: EDI1	0.4804	0.2704	1.7766	0.0766		-0.0517	1.0125
	Scanner: EDI2	-0.3254	0.2692	-1.2090	0.2276		-0.8551	0.2042
	Scanner: DUN	0.0247	0.2972	0.0830	0.9339		-0.5601	0.6094
	Scanner: GLA	-0.1490	0.2605	-0.5719	0.5678		-0.6616	0.3636
	Age	-0.1291	0.0539	-2.3936	0.0173		-0.2353	-0.0230
	Sex: female	0.2072	0.1241	1.6693	0.0961		-0.0371	0.4514
	WML	-0.1608	0.0544	-2.9541	0.0034		-0.2678	-0.0537
	Depression score	-0.1007	0.0621	-1.6220	0.1058		-0.2228	0.0215
Accumbens	Intercept	-0.5377	0.2522	-2.1318	0.0338		-1.0340	-0.0414
	Fatigue group: fatigued	0.2537	0.1191	2.1293	0.0340	0.7776	0.0192	0.4882
	Scanner: EDI1	0.0056	0.2596	0.0216	0.9828		-0.5053	0.5165
	Scanner: EDI2	0.1775	0.2584	0.6868	0.4927		-0.3311	0.6861
	Scanner: DUN	0.1461	0.2853	0.5119	0.6091		-0.4154	0.7075
	Scanner: GLA	0.1398	0.2501	0.5590	0.5766		-0.3524	0.6320
	Age	-0.2344	0.0518	-4.5256	0.0000		-0.3363	-0.1325
	Sex: female	0.4135	0.1192	3.4698	0.0006		0.1790	0.6480
	WML	-0.2961	0.0523	-5.6665	0.0000		-0.3989	-0.1933
	Depression score	-0.0817	0.0596	-1.3706	0.1715		-0.1990	0.0356
Amygdala	Intercept	0.1309	0.2627	0.4984	0.6186		-0.3860	0.6479
	Fatigue group: fatigued	0.0390	0.1241	0.3142	0.7536	0.8917	-0.2052	0.2832
	Scanner: EDI1	-0.5344	0.2704	-1.9765	0.0490		-1.0665	-0.0024
	Scanner: EDI2	-0.2159	0.2692	-0.8020	0.4232		-0.7455	0.3138
	Scanner: DUN	-0.3827	0.2972	-1.2879	0.1988		-0.9674	0.2020
	Scanner: GLA	-0.2162	0.2605	-0.8299	0.4072		-0.7288	0.2964
	Age	-0.2628	0.0539	-4.8728	0.0000		-0.3690	-0.1567
	Sex: female	0.1963	0.1241	1.5820	0.1147		-0.0479	0.4406
	WML	-0.1804	0.0544	-3.3156	0.0010		-0.2875	-0.0733
	Depression score	-0.0228	0.0621	-0.3677	0.7134		-0.1450	0.0993
Caudate	Intercept	-0.1392	0.2395	-0.5813	0.5615		-0.6105	0.3320
	Fatigue group: fatigued	0.2222	0.1131	1.9646	0.0504	0.7776	-0.0004	0.4448
	Scanner: EDI1	0.3607	0.2465	1.4635	0.1444		-0.1243	0.8458
	Scanner: EDI2	-0.6100	0.2454	-2.4861	0.0135		-1.0929	-0.1272
	Scanner: DUN	-0.5796	0.2709	-2.1398	0.0332		-1.1127	-0.0466
	Scanner: GLA	-0.3510	0.2375	-1.4782	0.1404		-0.8183	0.1163
	Age	-0.0816	0.0492	-1.6594	0.0981		-0.1784	0.0152
	Sex: female	0.3948	0.1131	3.4888	0.0006		0.1721	0.6174
	WML	-0.3134	0.0496	-6.3173	0.0000		-0.4110	-0.2158

	Depression score	-0.0445	0.0566	-0.7867	0.4321		-0.1559	0.0668
GM cerebellar	Intercept	-0.3245	0.2509	-1.2931	0.1970		-0.8182	0.1693
	Fatigue group: fatigued	-0.1759	0.1185	-1.4839	0.1389	0.8738	-0.4091	0.0574
	Scanner: EDI1	0.4270	0.2583	1.6533	0.0993		-0.0812	0.9352
	Scanner: EDI2	-0.2869	0.2571	-1.1159	0.2654		-0.7928	0.2190
	Scanner: DUN	0.2707	0.2838	0.9536	0.3410		-0.2879	0.8292
	Scanner: GLA	0.2079	0.2488	0.8354	0.4041		-0.2818	0.6975
	Age	-0.3384	0.0515	-6.5687	0.0000		-0.4398	-0.2371
	Sex: female	0.3532	0.1186	2.9796	0.0031		0.1199	0.5865
	WML	-0.0345	0.0520	-0.6630	0.5078		-0.1368	0.0678
	Depression score	-0.0273	0.0593	-0.4612	0.6450		-0.1440	0.0893
GM cerebral	Intercept	-0.6429	0.2324	-2.7667	0.0060		-1.1001	-0.1856
	Fatigue group: fatigued	0.0698	0.1098	0.6359	0.5253	0.9673	-0.1462	0.2858
	Scanner: EDI1	0.7847	0.2392	3.2808	0.0012		0.3140	1.2553
	Scanner: EDI2	-0.1511	0.2381	-0.6348	0.5261		-0.6196	0.3174
	Scanner: DUN	0.2319	0.2628	0.8823	0.3783		-0.2853	0.7491
	Scanner: GLA	0.4069	0.2304	1.7660	0.0784		-0.0465	0.8603
	Age	-0.4054	0.0477	-8.4972	0.0000		-0.4993	-0.3115
	Sex: female	0.3826	0.1098	3.4853	0.0006		0.1666	0.5987
	WML	-0.1192	0.0481	-2.4771	0.0138		-0.2140	-0.0245
	Depression score	-0.0548	0.0549	-0.9988	0.3187		-0.1629	0.0532
Hippocampus	Intercept	0.1135	0.2784	0.4077	0.6838		-0.4344	0.6614
	Fatigue group: fatigued	-0.0054	0.1315	-0.0410	0.9673	0.8738	-0.2642	0.2534
	Scanner: EDI1	-0.2369	0.2866	-0.8267	0.4090		-0.8009	0.3270
	Scanner: EDI2	-0.2464	0.2853	-0.8636	0.3885		-0.8078	0.3150
	Scanner: DUN	-0.3909	0.3150	-1.2410	0.2156		-1.0106	0.2289
	Scanner: GLA	-0.2544	0.2761	-0.9215	0.3575		-0.7978	0.2889
	Age	-0.1325	0.0572	-2.3176	0.0211		-0.2450	-0.0200
	Sex: female	0.1867	0.1316	1.4189	0.1570		-0.0722	0.4455
	WML	-0.0693	0.0577	-1.2017	0.2304		-0.1828	0.0442
	Depression score	-0.0223	0.0658	-0.3388	0.7350		-0.1518	0.1072
NAWM cerebral	Intercept	-0.0127	0.2497	-0.0510	0.9594		-0.5041	0.4786
	Fatigue group: fatigued	0.1236	0.1179	1.0479	0.2955	0.8738	-0.1085	0.3557
	Scanner: EDI1	0.2051	0.2570	0.7982	0.4254		-0.3006	0.7109
	Scanner: EDI2	-0.5966	0.2558	-2.3319	0.0204		-1.1000	-0.0931
	Scanner: DUN	-0.0887	0.2824	-0.3141	0.7536		-0.6445	0.4671
	Scanner: GLA	-0.1425	0.2476	-0.5755	0.5654		-0.6297	0.3447
	Age	-0.0208	0.0513	-0.4048	0.6859		-0.1216	0.0801
	Sex: female	0.1512	0.1180	1.2817	0.2009		-0.0809	0.3833
	WML	-0.3903	0.0517	-7.5460	0.0000		-0.4921	-0.2885
	Depression score	-0.0057	0.0590	-0.0959	0.9236		-0.1218	0.1104
NAWM cerebellar	Intercept	0.0845	0.2781	0.3038	0.7615		-0.4628	0.6317
	Fatigue group: fatigued	-0.0963	0.1314	-0.7331	0.4640	0.8738	-0.3548	0.1622
	Scanner: EDI1	-0.1438	0.2862	-0.5023	0.6158		-0.7071	0.4195

	Scanner: EDI2	0.2750	0.2849	0.9652	0.3352		-0.2857	0.8358
	Scanner: DUN	0.1435	0.3146	0.4562	0.6486		-0.4755	0.7625
	Scanner: GLA	-0.1628	0.2758	-0.5903	0.5554		-0.7055	0.3799
	Age	0.0350	0.0571	0.6129	0.5404		-0.0774	0.1474
	Sex: female	-0.0340	0.1314	-0.2586	0.7961		-0.2925	0.2246
	WML	-0.0451	0.0576	-0.7836	0.4339		-0.1585	0.0682
	Depression score	-0.0178	0.0657	-0.2710	0.7866		-0.1471	0.1115
Pallidum	Intercept	-0.0482	0.2803	-0.1720	0.8636		-0.5998	0.5034
	Fatigue group: fatigued	-0.0799	0.1324	-0.6036	0.5466	0.7887	-0.3405	0.1806
	Scanner: EDI1	0.0847	0.2885	0.2936	0.7693		-0.4831	0.6525
	Scanner: EDI2	0.3914	0.2872	1.3628	0.1740		-0.1738	0.9566
	Scanner: DUN	0.1855	0.3171	0.5850	0.5590		-0.4385	0.8095
	Scanner: GLA	0.1229	0.2780	0.4423	0.6586		-0.4241	0.6700
	Age	0.0399	0.0576	0.6937	0.4884		-0.0733	0.1532
	Sex: female	-0.1243	0.1324	-0.9385	0.3487		-0.3849	0.1363
	WML	-0.0698	0.0581	-1.2025	0.2301		-0.1841	0.0444
	Depression score	-0.0135	0.0662	-0.2038	0.8386		-0.1438	0.1168
Putamen	Intercept	-0.5750	0.2482	-2.3166	0.0212		-1.0635	-0.0866
	Fatigue group: fatigued	0.1653	0.1173	1.4098	0.1596	0.8738	-0.0654	0.3960
	Scanner: EDI1	0.3552	0.2555	1.3903	0.1655		-0.1476	0.8580
	Scanner: EDI2	0.0758	0.2543	0.2980	0.7659		-0.4247	0.5763
	Scanner: DUN	0.3092	0.2808	1.1013	0.2717		-0.2433	0.8617
	Scanner: GLA	0.1782	0.2461	0.7239	0.4697		-0.3062	0.6625
	Age	-0.3430	0.0510	-6.7292	0.0000		-0.4433	-0.2427
	Sex: female	0.3994	0.1173	3.4059	0.0007		0.1687	0.6302
	WML	-0.2380	0.0514	-4.6289	0.0000		-0.3392	-0.1368
	Depression score	-0.0358	0.0587	-0.6098	0.5425		-0.1512	0.0797
Thalamus	Intercept	0.0876	0.2809	0.3121	0.7552		-0.4650	0.6403
	Fatigue group: fatigued	-0.1082	0.1327	-0.8156	0.4154	0.7887	-0.3693	0.1529
	Scanner: EDI1	0.0629	0.2891	0.2176	0.8279		-0.5060	0.6318
	Scanner: EDI2	0.2713	0.2878	0.9427	0.3466		-0.2950	0.8376
	Scanner: DUN	0.0646	0.3177	0.2033	0.8390		-0.5606	0.6898
	Scanner: GLA	0.0360	0.2785	0.1291	0.8974		-0.5121	0.5840
	Age	0.0210	0.0577	0.3640	0.7161		-0.0925	0.1345
	Sex: female	-0.1823	0.1327	-1.3741	0.1704		-0.4435	0.0788
	WML	-0.0591	0.0582	-1.0161	0.3104		-0.1736	0.0554
	Depression score	-0.0058	0.0664	-0.0872	0.9306		-0.1364	0.1248
Ventral Diencephalon	Intercept	-0.0915	0.2661	-0.3439	0.7311		-0.6152	0.4322
	Fatigue group: fatigued	0.1780	0.1257	1.4158	0.1579	0.7776	-0.0694	0.4253
	Scanner: EDI1	-0.0960	0.2739	-0.3505	0.7262		-0.6350	0.4430
	Scanner: EDI2	-0.1955	0.2727	-0.7171	0.4738		-0.7321	0.3410
	Scanner: DUN	-0.1572	0.3010	-0.5222	0.6019		-0.7496	0.4352
	Scanner: GLA	-0.3128	0.2639	-1.1854	0.2368		-0.8321	0.2065
	Age	-0.1477	0.0546	-2.7034	0.0073		-0.2553	-0.0402

	Sex: female	0.2866	0.1257	2.2793	0.0233		0.0392	0.5340
	WML	-0.2443	0.0551	-4.4306	0.0000		-0.3527	-0.1358
	Depression score	-0.0556	0.0629	-0.8846	0.3770		-0.1794	0.0681
GM banks sts	Intercept	-0.5875	0.2541	-2.3123	0.0214		-1.0874	-0.0875
	Fatigue group: fatigued	-0.1832	0.1200	-1.5263	0.1280	0.7776	-0.4193	0.0530
	Scanner: EDI1	0.5563	0.2615	2.1274	0.0342		0.0417	1.0709
	Scanner: EDI2	0.6061	0.2603	2.3282	0.0206		0.0938	1.1183
	Scanner: DUN	0.4395	0.2874	1.5294	0.1272		-0.1260	1.0051
	Scanner: GLA	0.2776	0.2519	1.1020	0.2713		-0.2181	0.7734
	Age	-0.3496	0.0522	-6.7015	0.0000		-0.4523	-0.2469
	Sex: female	0.3314	0.1200	2.7611	0.0061		0.0952	0.5676
	WML	-0.0553	0.0526	-1.0516	0.2938		-0.1589	0.0482
	Depression score	0.0565	0.0600	0.9419	0.3470		-0.0616	0.1747
GM caudal anterior cingulate	Intercept	-0.7711	0.2574	-2.9964	0.0030		-1.2776	-0.2647
	Fatigue group: fatigued	0.1819	0.1216	1.4964	0.1356	0.7776	-0.0573	0.4211
	Scanner: EDI1	0.6784	0.2649	2.5612	0.0109		0.1572	1.1997
	Scanner: EDI2	0.0275	0.2637	0.1041	0.9171		-0.4914	0.5464
	Scanner: DUN	0.9557	0.2911	3.2831	0.0011		0.3829	1.5286
	Scanner: GLA	0.4140	0.2552	1.6221	0.1058		-0.0882	0.9162
	Age	-0.2000	0.0528	-3.7848	0.0002		-0.3040	-0.0960
	Sex: female	0.3540	0.1216	2.9113	0.0039		0.1147	0.5933
	WML	-0.1522	0.0533	-2.8541	0.0046		-0.2571	-0.0472
	Depression score	-0.1153	0.0608	-1.8962	0.0589		-0.2350	0.0044
GM caudal middle frontal	Intercept	-0.3507	0.2462	-1.4248	0.1552		-0.8352	0.1337
	Fatigue group: fatigued	0.0616	0.1163	0.5300	0.5965	0.8738	-0.1672	0.2905
	Scanner: EDI1	0.5210	0.2534	2.0560	0.0406		0.0223	1.0196
	Scanner: EDI2	-0.6142	0.2522	-2.4350	0.0155		-1.1106	-0.1179
	Scanner: DUN	-0.0302	0.2785	-0.1086	0.9136		-0.5782	0.5177
	Scanner: GLA	0.1723	0.2441	0.7057	0.4809		-0.3081	0.6526
	Age	-0.2324	0.0505	-4.5973	0.0000		-0.3318	-0.1329
	Sex: female	0.3797	0.1163	3.2647	0.0012		0.1508	0.6086
	WML	-0.0768	0.0510	-1.5068	0.1329		-0.1772	0.0235
	Depression score	-0.0753	0.0582	-1.2948	0.1964		-0.1898	0.0392
GM cuneus	Intercept	-0.2855	0.2760	-1.0343	0.3018		-0.8287	0.2577
	Fatigue group: fatigued	0.0747	0.1304	0.5726	0.5674	0.8738	-0.1819	0.3312
	Scanner: EDI1	0.3642	0.2841	1.2818	0.2009		-0.1949	0.9233
	Scanner: EDI2	0.2161	0.2828	0.7640	0.4455		-0.3405	0.7726
	Scanner: DUN	0.0450	0.3122	0.1443	0.8854		-0.5694	0.6595
	Scanner: GLA	0.4395	0.2737	1.6057	0.1094		-0.0991	0.9782
	Age	-0.1464	0.0567	-2.5836	0.0102		-0.2580	-0.0349
	Sex: female	-0.0729	0.1304	-0.5592	0.5765		-0.3296	0.1837
	WML	-0.0638	0.0572	-1.1166	0.2651		-0.1764	0.0487
	Depression score	-0.0465	0.0652	-0.7125	0.4767		-0.1748	0.0819
GM ent	Intercept	-0.4575	0.2698	-1.6955	0.0910		-0.9885	0.0735

	Fatigue group: fatigued	-0.1483	0.1275	-1.1633	0.2456	0.8738	-0.3991	0.1026
	Scanner: EDI1	0.8842	0.2777	3.1833	0.0016		0.3376	1.4307
	Scanner: EDI2	0.2961	0.2765	1.0709	0.2851		-0.2480	0.8402
	Scanner: DUN	0.7136	0.3052	2.3379	0.0200		0.1130	1.3143
	Scanner: GLA	0.6866	0.2676	2.5659	0.0108		0.1600	1.2132
	Age	-0.0835	0.0554	-1.5074	0.1327		-0.1926	0.0255
	Sex: female	-0.1140	0.1275	-0.8941	0.3720		-0.3649	0.1369
	WML	-0.1601	0.0559	-2.8649	0.0045		-0.2701	-0.0501
	Depression score	0.0718	0.0638	1.1268	0.2607		-0.0536	0.1973
GM frontal pole	Intercept	-0.1360	0.2494	-0.5453	0.5860		-0.6268	0.3548
	Fatigue group: fatigued	-0.0842	0.1178	-0.7145	0.4755	0.8738	-0.3160	0.1477
	Scanner: EDI1	0.1605	0.2567	0.6254	0.5322		-0.3446	0.6657
	Scanner: EDI2	-0.0825	0.2556	-0.3228	0.7471		-0.5854	0.4204
	Scanner: DUN	-0.1603	0.2821	-0.5683	0.5702		-0.7155	0.3948
	Scanner: GLA	0.1033	0.2473	0.4177	0.6764		-0.3834	0.5900
	Age	-0.4352	0.0512	-8.4980	0.0000		-0.5360	-0.3344
	Sex: female	0.1796	0.1178	1.5238	0.1286		-0.0523	0.4115
	WML	-0.0576	0.0517	-1.1146	0.2659		-0.1593	0.0441
	Depression score	0.0019	0.0589	0.0325	0.9741		-0.1141	0.1179
GM fusiform	Intercept	-0.7910	0.2521	-3.1375	0.0019		-1.2871	-0.2949
	Fatigue group: fatigued	-0.0289	0.1191	-0.2423	0.8087	0.9019	-0.2632	0.2055
	Scanner: EDI1	1.1338	0.2595	4.3692	0.0000		0.6232	1.6445
	Scanner: EDI2	0.4579	0.2583	1.7727	0.0773		-0.0504	0.9663
	Scanner: DUN	0.6862	0.2852	2.4061	0.0167		0.1250	1.2474
	Scanner: GLA	0.7894	0.2500	3.1573	0.0018		0.2974	1.2813
	Age	-0.2651	0.0518	-5.1211	0.0000		-0.3670	-0.1632
	Sex: female	0.0838	0.1191	0.7038	0.4821		-0.1506	0.3182
	WML	-0.2019	0.0522	-3.8664	0.0001		-0.3047	-0.0992
	Depression score	-0.0501	0.0596	-0.8402	0.4014		-0.1673	0.0672
GM inferior parietal	Intercept	-0.7659	0.2551	-3.0024	0.0029		-1.2679	-0.2639
	Fatigue group: fatigued	-0.1788	0.1205	-1.4840	0.1388	0.7776	-0.4160	0.0583
	Scanner: EDI1	0.9763	0.2626	3.7180	0.0002		0.4596	1.4930
	Scanner: EDI2	0.4714	0.2614	1.8035	0.0723		-0.0429	0.9858
	Scanner: DUN	0.5601	0.2886	1.9408	0.0532		-0.0078	1.1279
	Scanner: GLA	0.4462	0.2530	1.7637	0.0788		-0.0516	0.9440
	Age	-0.2674	0.0524	-5.1057	0.0000		-0.3705	-0.1644
	Sex: female	0.3780	0.1205	3.1357	0.0019		0.1408	0.6151
	WML	-0.0853	0.0528	-1.6134	0.1077		-0.1893	0.0187
	Depression score	-0.0027	0.0603	-0.0454	0.9638		-0.1214	0.1159
GM inferior temporal	Intercept	-0.4771	0.2537	-1.8803	0.0610		-0.9764	0.0222
	Fatigue group: fatigued	-0.1477	0.1199	-1.2325	0.2187	0.8738	-0.3836	0.0881
	Scanner: EDI1	0.6894	0.2612	2.6399	0.0087		0.1755	1.2033
	Scanner: EDI2	-0.0119	0.2600	-0.0457	0.9636		-0.5235	0.4997
	Scanner: DUN	0.5192	0.2870	1.8090	0.0714		-0.0456	1.0840

	Scanner: GLA	0.4990	0.2516	1.9833	0.0482		0.0039	0.9941
	Age	-0.3151	0.0521	-6.0491	0.0000		-0.4177	-0.2126
	Sex: female	0.1902	0.1199	1.5862	0.1137		-0.0457	0.4261
	WML	-0.1014	0.0526	-1.9289	0.0547		-0.2048	0.0020
	Depression score	0.0024	0.0600	0.0406	0.9677		-0.1156	0.1204
GM insula	Intercept	-0.0083	0.2552	-0.0325	0.9741		-0.5104	0.4939
	Fatigue group: fatigued	0.0663	0.1205	0.5499	0.5828	0.8738	-0.1709	0.3035
	Scanner: EDI1	0.4109	0.2627	1.5645	0.1187		-0.1059	0.9278
	Scanner: EDI2	-0.3441	0.2615	-1.3161	0.1891		-0.8586	0.1704
	Scanner: DUN	-0.4405	0.2887	-1.5261	0.1280		-1.0085	0.1275
	Scanner: GLA	-0.1664	0.2530	-0.6577	0.5112		-0.6644	0.3315
	Age	-0.2981	0.0524	-5.6902	0.0000		-0.4013	-0.1950
	Sex: female	0.1025	0.1206	0.8498	0.3961		-0.1348	0.3397
	WML	-0.0065	0.0529	-0.1232	0.9020		-0.1105	0.0975
	Depression score	-0.1087	0.0603	-1.8022	0.0725		-0.2273	0.0100
GM isthmus cingulate	Intercept	-0.1327	0.2655	-0.4999	0.6175		-0.6553	0.3898
	Fatigue group: fatigued	0.2092	0.1254	1.6674	0.0965	0.7776	-0.0377	0.4560
	Scanner: EDI1	0.3443	0.2733	1.2597	0.2087		-0.1935	0.8822
	Scanner: EDI2	-0.3207	0.2721	-1.1786	0.2395		-0.8561	0.2147
	Scanner: DUN	-0.0603	0.3004	-0.2007	0.8411		-0.6514	0.5308
	Scanner: GLA	0.1821	0.2633	0.6915	0.4898		-0.3361	0.7003
	Age	-0.2172	0.0545	-3.9830	0.0001		-0.3245	-0.1099
	Sex: female	-0.0383	0.1255	-0.3049	0.7607		-0.2851	0.2086
	WML	-0.1038	0.0550	-1.8877	0.0600		-0.2121	0.0044
	Depression score	-0.0372	0.0627	-0.5930	0.5537		-0.1607	0.0863
GM lateral occipital	Intercept	-0.6380	0.2637	-2.4195	0.0161		-1.1570	-0.1191
	Fatigue group: fatigued	0.0623	0.1246	0.5002	0.6173	0.8738	-0.1828	0.3074
	Scanner: EDI1	0.9536	0.2714	3.5132	0.0005		0.4195	1.4877
	Scanner: EDI2	0.4269	0.2702	1.5799	0.1152		-0.1048	0.9586
	Scanner: DUN	0.7424	0.2983	2.4889	0.0134		0.1554	1.3294
	Scanner: GLA	0.8001	0.2615	3.0599	0.0024		0.2856	1.3147
	Age	-0.2622	0.0541	-4.8427	0.0000		-0.3688	-0.1557
	Sex: female	-0.1246	0.1246	-0.9997	0.3183		-0.3697	0.1206
	WML	-0.0282	0.0546	-0.5153	0.6067		-0.1356	0.0793
	Depression score	-0.0161	0.0623	-0.2576	0.7969		-0.1387	0.1066
GM lateral orbitofrontal	Intercept	-0.6581	0.2469	-2.6657	0.0081		-1.1439	-0.1723
	Fatigue group: fatigued	0.0712	0.1166	0.6103	0.5421	0.8738	-0.1583	0.3007
	Scanner: EDI1	0.7964	0.2541	3.1342	0.0019		0.2964	1.2965
	Scanner: EDI2	0.1923	0.2530	0.7601	0.4478		-0.3055	0.6901
	Scanner: DUN	0.3689	0.2793	1.3210	0.1875		-0.1806	0.9185
	Scanner: GLA	0.5373	0.2448	2.1947	0.0289		0.0555	1.0191
	Age	-0.3864	0.0507	-7.6217	0.0000		-0.4861	-0.2866
	Sex: female	0.2055	0.1166	1.7614	0.0792		-0.0241	0.4350
	WML	-0.1081	0.0511	-2.1143	0.0353		-0.2088	-0.0075

	Depression score	-0.0716	0.0583	-1.2280	0.2204		-0.1864	0.0432
GM lingual	Intercept	-0.2261	0.2735	-0.8269	0.4089		-0.7643	0.3120
	Fatigue group: fatigued	0.0574	0.1292	0.4441	0.6573	0.8764	-0.1968	0.3116
	Scanner: EDI1	0.3058	0.2815	1.0865	0.2781		-0.2481	0.8597
	Scanner: EDI2	0.2197	0.2802	0.7841	0.4336		-0.3317	0.7711
	Scanner: DUN	-0.0026	0.3093	-0.0082	0.9934		-0.6113	0.6062
	Scanner: GLA	0.1494	0.2712	0.5509	0.5821		-0.3842	0.6830
	Age	-0.1971	0.0562	-3.5095	0.0005		-0.3076	-0.0866
	Sex: female	0.0300	0.1292	0.2318	0.8168		-0.2243	0.2842
	WML	-0.1228	0.0566	-2.1677	0.0310		-0.2343	-0.0113
	Depression score	0.0064	0.0646	0.0991	0.9211		-0.1208	0.1336
GM medial orbitofrontal	Intercept	-0.3407	0.2460	-1.3848	0.1671		-0.8248	0.1434
	Fatigue group: fatigued	0.1410	0.1162	1.2132	0.2260	0.8738	-0.0877	0.3697
	Scanner: EDI1	0.3885	0.2532	1.5344	0.1260		-0.1098	0.8868
	Scanner: EDI2	0.0639	0.2521	0.2534	0.8001		-0.4322	0.5599
	Scanner: DUN	-0.3468	0.2783	-1.2464	0.2136		-0.8945	0.2008
	Scanner: GLA	0.3078	0.2440	1.2616	0.2081		-0.1723	0.7878
	Age	-0.3882	0.0505	-7.6842	0.0000		-0.4876	-0.2888
	Sex: female	0.1244	0.1162	1.0698	0.2855		-0.1044	0.3531
	WML	-0.1096	0.0510	-2.1515	0.0322		-0.2099	-0.0094
	Depression score	-0.0974	0.0581	-1.6761	0.0947		-0.2118	0.0170
GM middle temporal	Intercept	-0.4307	0.2410	-1.7875	0.0749		-0.9049	0.0434
	Fatigue group: fatigued	-0.2014	0.1138	-1.7699	0.0778	0.7776	-0.4254	0.0225
	Scanner: EDI1	0.7072	0.2480	2.8516	0.0046		0.2192	1.1953
	Scanner: EDI2	0.2456	0.2469	0.9947	0.3207		-0.2402	0.7314
	Scanner: DUN	0.2088	0.2726	0.7662	0.4442		-0.3275	0.7452
	Scanner: GLA	0.3050	0.2389	1.2765	0.2028		-0.1652	0.7752
	Age	-0.4422	0.0495	-8.9385	0.0000		-0.5396	-0.3449
	Sex: female	0.2195	0.1138	1.9278	0.0548		-0.0046	0.4435
	WML	-0.0185	0.0499	-0.3716	0.7105		-0.1168	0.0797
	Depression score	-0.0286	0.0569	-0.5016	0.6163		-0.1406	0.0835
GM paracentral	Intercept	-0.7131	0.2493	-2.8610	0.0045		-1.2036	-0.2226
	Fatigue group: fatigued	0.0408	0.1177	0.3465	0.7292	0.8917	-0.1909	0.2725
	Scanner: EDI1	0.6827	0.2566	2.6612	0.0082		0.1779	1.1876
	Scanner: EDI2	-0.2222	0.2554	-0.8702	0.3849		-0.7248	0.2803
	Scanner: DUN	0.3277	0.2820	1.1621	0.2461		-0.2272	0.8825
	Scanner: GLA	0.3644	0.2472	1.4742	0.1415		-0.1220	0.8508
	Age	-0.2299	0.0512	-4.4912	0.0000		-0.3306	-0.1291
	Sex: female	0.5545	0.1178	4.7080	0.0000		0.3227	0.7862
	WML	-0.1147	0.0516	-2.2216	0.0270		-0.2163	-0.0131
	Depression score	-0.0311	0.0589	-0.5274	0.5983		-0.1470	0.0848
GM parahippoca	Intercept	-0.7570	0.2577	-2.9380	0.0036		-1.2641	-0.2500
	Fatigue group: fatigued	0.0431	0.1217	0.3542	0.7235	0.8917	-0.1964	0.2826
	Scanner: EDI1	0.3960	0.2652	1.4929	0.1365		-0.1259	0.9179

	Scanner: EDI2	0.2170	0.2640	0.8217	0.4119		-0.3026	0.7365
	Scanner: DUN	0.0181	0.2915	0.0623	0.9504		-0.5554	0.5917
	Scanner: GLA	0.3890	0.2555	1.5225	0.1289		-0.1138	0.8918
	Age	-0.2078	0.0529	-3.9282	0.0001		-0.3120	-0.1037
	Sex: female	0.5940	0.1217	4.8789	0.0000		0.3544	0.8335
	WML	-0.1364	0.0534	-2.5560	0.0111		-0.2415	-0.0314
	Depression score	-0.1049	0.0609	-1.7231	0.0859		-0.2247	0.0149
GM pars opercularis	Intercept	-0.0816	0.2484	-0.3282	0.7430		-0.5705	0.4074
	Fatigue group: fatigued	-0.0346	0.1174	-0.2947	0.7684	0.8917	-0.2655	0.1964
	Scanner: EDI1	0.1066	0.2557	0.4167	0.6772		-0.3967	0.6098
	Scanner: EDI2	-0.4246	0.2546	-1.6678	0.0964		-0.9255	0.0764
	Scanner: DUN	-0.2984	0.2810	-1.0619	0.2891		-0.8515	0.2546
	Scanner: GLA	-0.1712	0.2464	-0.6948	0.4877		-0.6560	0.3136
	Age	-0.3811	0.0510	-7.4710	0.0000		-0.4815	-0.2807
	Sex: female	0.3598	0.1174	3.0650	0.0024		0.1288	0.5908
	WML	-0.1141	0.0515	-2.2176	0.0273		-0.2154	-0.0129
	Depression score	-0.0518	0.0587	-0.8818	0.3786		-0.1673	0.0638
GM pars orbitalis	Intercept	-0.6288	0.2516	-2.4995	0.0130		-1.1239	-0.1337
	Fatigue group: fatigued	0.0149	0.1188	0.1250	0.9006	0.9339	-0.2190	0.2487
	Scanner: EDI1	0.7190	0.2589	2.7766	0.0058		0.2094	1.2285
	Scanner: EDI2	0.4212	0.2578	1.6338	0.1033		-0.0861	0.9284
	Scanner: DUN	0.4402	0.2846	1.5470	0.1229		-0.1198	1.0002
	Scanner: GLA	0.4818	0.2495	1.9312	0.0544		-0.0091	0.9727
	Age	-0.4176	0.0517	-8.0839	0.0000		-0.5192	-0.3159
	Sex: female	0.1708	0.1189	1.4367	0.1518		-0.0631	0.4047
	WML	0.0183	0.0521	0.3512	0.7257		-0.0843	0.1209
	Depression score	-0.0378	0.0594	-0.6354	0.5257		-0.1548	0.0792
GM pars triangularis	Intercept	-0.2455	0.2578	-0.9525	0.3416		-0.7527	0.2617
	Fatigue group: fatigued	0.1273	0.1218	1.0457	0.2965	0.8738	-0.1123	0.3669
	Scanner: EDI1	0.4357	0.2653	1.6424	0.1015		-0.0863	0.9578
	Scanner: EDI2	-0.0979	0.2641	-0.3706	0.7112		-0.6176	0.4218
	Scanner: DUN	-0.0681	0.2916	-0.2335	0.8155		-0.6418	0.5057
	Scanner: GLA	0.1267	0.2556	0.4956	0.6205		-0.3763	0.6297
	Age	-0.3630	0.0529	-6.8580	0.0000		-0.4671	-0.2588
	Sex: female	0.0940	0.1218	0.7717	0.4409		-0.1457	0.3336
	WML	0.0402	0.0534	0.7538	0.4516		-0.0648	0.1453
	Depression score	-0.0696	0.0609	-1.1435	0.2537		-0.1895	0.0502
GM pericalcarine	Intercept	-0.4876	0.2733	-1.7842	0.0754		-1.0253	0.0502
	Fatigue group: fatigued	-0.0865	0.1291	-0.6704	0.5031	0.8738	-0.3406	0.1675
	Scanner: EDI1	0.3977	0.2813	1.4138	0.1584		-0.1558	0.9511
	Scanner: EDI2	0.6327	0.2800	2.2596	0.0246		0.0817	1.1837
	Scanner: DUN	0.0948	0.3091	0.3068	0.7592		-0.5135	0.7031
	Scanner: GLA	0.3154	0.2710	1.1641	0.2453		-0.2178	0.8487
	Age	-0.1296	0.0561	-2.3091	0.0216		-0.2400	-0.0192

	Sex: female	0.2102	0.1291	1.6281	0.1046		-0.0439	0.4643
	WML	-0.0530	0.0566	-0.9359	0.3501		-0.1644	0.0584
	Depression score	0.0557	0.0646	0.8629	0.3889		-0.0713	0.1828
GM postcentral	Intercept	-0.5844	0.2535	-2.3050	0.0218		-1.0832	-0.0855
	Fatigue group: fatigued	-0.1010	0.1198	-0.8433	0.3997	0.8738	-0.3366	0.1347
	Scanner: EDI1	1.0291	0.2609	3.9439	0.0001		0.5156	1.5426
	Scanner: EDI2	0.1479	0.2598	0.5695	0.5694		-0.3632	0.6591
	Scanner: DUN	0.3528	0.2868	1.2302	0.2196		-0.2115	0.9171
	Scanner: GLA	0.4425	0.2514	1.7603	0.0794		-0.0522	0.9373
	Age	-0.2109	0.0521	-4.0516	0.0001		-0.3133	-0.1085
	Sex: female	0.2054	0.1198	1.7151	0.0874		-0.0303	0.4411
	WML	-0.1667	0.0525	-3.1733	0.0017		-0.2700	-0.0633
	Depression score	0.0178	0.0599	0.2963	0.7672		-0.1001	0.1356
GM posterior cingulate	Intercept	-0.2809	0.2488	-1.1294	0.2596		-0.7704	0.2086
	Fatigue group: fatigued	0.1824	0.1175	1.5520	0.1217	0.7776	-0.0489	0.4136
	Scanner: EDI1	0.5231	0.2560	2.0430	0.0419		0.0192	1.0269
	Scanner: EDI2	-0.2476	0.2549	-0.9714	0.3321		-0.7491	0.2540
	Scanner: DUN	0.1500	0.2814	0.5331	0.5944		-0.4037	0.7037
	Scanner: GLA	0.0458	0.2467	0.1858	0.8528		-0.4396	0.5312
	Age	-0.3666	0.0511	-7.1768	0.0000		-0.4671	-0.2661
	Sex: female	0.1377	0.1175	1.1720	0.2421		-0.0935	0.3690
	WML	-0.1306	0.0515	-2.5343	0.0118		-0.2320	-0.0292
	Depression score	-0.0483	0.0588	-0.8217	0.4119		-0.1640	0.0674
GM precentral	Intercept	-0.6574	0.2450	-2.6837	0.0077		-1.1395	-0.1754
	Fatigue group: fatigued	0.0328	0.1157	0.2837	0.7768	0.8917	-0.1949	0.2605
	Scanner: EDI1	0.9959	0.2521	3.9497	0.0001		0.4997	1.4921
	Scanner: EDI2	-0.1628	0.2510	-0.6484	0.5172		-0.6567	0.3312
	Scanner: DUN	0.4688	0.2771	1.6920	0.0917		-0.0764	1.0141
	Scanner: GLA	0.5038	0.2429	2.0738	0.0389		0.0257	0.9818
	Age	-0.2211	0.0503	-4.3966	0.0000		-0.3201	-0.1222
	Sex: female	0.2804	0.1157	2.4224	0.0160		0.0526	0.5081
	WML	-0.1409	0.0507	-2.7774	0.0058		-0.2408	-0.0411
	Depression score	-0.0800	0.0579	-1.3813	0.1682		-0.1939	0.0340
GM precuneus	Intercept	-0.2052	0.2630	-0.7802	0.4359		-0.7226	0.3123
	Fatigue group: fatigued	-0.0422	0.1242	-0.3398	0.7343	0.8917	-0.2866	0.2022
	Scanner: EDI1	0.4000	0.2707	1.4779	0.1405		-0.1326	0.9326
	Scanner: EDI2	-0.1160	0.2694	-0.4304	0.6672		-0.6461	0.4142
	Scanner: DUN	-0.2777	0.2974	-0.9336	0.3512		-0.8630	0.3076
	Scanner: GLA	0.1098	0.2608	0.4210	0.6741		-0.4034	0.6229
	Age	-0.2465	0.0540	-4.5654	0.0000		-0.3528	-0.1403
	Sex: female	0.1981	0.1242	1.5941	0.1120		-0.0464	0.4425
	WML	-0.1079	0.0545	-1.9814	0.0485		-0.2151	-0.0007
	Depression score	-0.0080	0.0621	-0.1289	0.8975		-0.1303	0.1143
GM ros	Intercept	-0.5371	0.2552	-2.1042	0.0362		-1.0393	-0.0348

	Fatigue group: fatigued	0.0568	0.1206	0.4710	0.6380	0.8738	-0.1805	0.2940
	Scanner: EDI1	0.7404	0.2627	2.8182	0.0051		0.2234	1.2574
	Scanner: EDI2	0.2074	0.2615	0.7932	0.4283		-0.3072	0.7221
	Scanner: DUN	0.8488	0.2887	2.9399	0.0035		0.2807	1.4169
	Scanner: GLA	0.5591	0.2531	2.2088	0.0279		0.0610	1.0571
	Age	-0.3015	0.0524	-5.7535	0.0000		-0.4047	-0.1984
	Sex: female	-0.0154	0.1206	-0.1275	0.8986		-0.2527	0.2219
	WML	-0.1341	0.0529	-2.5363	0.0117		-0.2381	-0.0301
	Depression score	-0.1687	0.0603	-2.7967	0.0055		-0.2874	-0.0500
GM rostral middle frontal	Intercept	-0.4658	0.2460	-1.8934	0.0593		-0.9500	0.0183
	Fatigue group: fatigued	0.0586	0.1162	0.5044	0.6143	0.8738	-0.1701	0.2873
	Scanner: EDI1	0.7917	0.2532	3.1263	0.0019		0.2934	1.2900
	Scanner: EDI2	0.0657	0.2521	0.2606	0.7946		-0.4304	0.5618
	Scanner: DUN	0.4248	0.2783	1.5265	0.1279		-0.1228	0.9725
	Scanner: GLA	0.6198	0.2440	2.5404	0.0116		0.1397	1.0999
	Age	-0.3710	0.0505	-7.3446	0.0000		-0.4704	-0.2716
	Sex: female	-0.0525	0.1162	-0.4519	0.6516		-0.2813	0.1762
	WML	-0.1008	0.0510	-1.9785	0.0488		-0.2011	-0.0005
	Depression score	-0.1018	0.0581	-1.7510	0.0810		-0.2162	0.0126
GM superior frontal	Intercept	-0.1632	0.2237	-0.7295	0.4662		-0.6035	0.2770
	Fatigue group: fatigued	0.0836	0.1057	0.7912	0.4294	0.8738	-0.1243	0.2916
	Scanner: EDI1	0.5997	0.2303	2.6043	0.0097		0.1466	1.0529
	Scanner: EDI2	-0.6966	0.2292	-3.0389	0.0026		-1.1478	-0.2455
	Scanner: DUN	-0.1991	0.2531	-0.7866	0.4321		-0.6971	0.2989
	Scanner: GLA	0.1126	0.2219	0.5074	0.6122		-0.3240	0.5492
	Age	-0.3902	0.0459	-8.4935	0.0000		-0.4806	-0.2998
	Sex: female	0.1734	0.1057	1.6403	0.1020		-0.0346	0.3814
	WML	-0.0957	0.0463	-2.0659	0.0397		-0.1870	-0.0045
	Depression score	-0.0669	0.0529	-1.2659	0.2065		-0.1710	0.0371
GM superior parietal	Intercept	-0.8636	0.2566	-3.3650	0.0009		-1.3686	-0.3586
	Fatigue group: fatigued	-0.0067	0.1212	-0.0551	0.9561	0.9673	-0.2452	0.2319
	Scanner: EDI1	0.8862	0.2641	3.3548	0.0009		0.3664	1.4060
	Scanner: EDI2	0.2541	0.2630	0.9663	0.3346		-0.2633	0.7715
	Scanner: DUN	0.2564	0.2903	0.8833	0.3778		-0.3148	0.8277
	Scanner: GLA	0.6730	0.2545	2.6446	0.0086		0.1722	1.1738
	Age	-0.1854	0.0527	-3.5180	0.0005		-0.2891	-0.0817
	Sex: female	0.4268	0.1213	3.5198	0.0005		0.1882	0.6654
	WML	-0.1382	0.0532	-2.5987	0.0098		-0.2428	-0.0335
	Depression score	-0.0564	0.0606	-0.9297	0.3532		-0.1757	0.0630
GM superior temporal	Intercept	-0.4739	0.2459	-1.9274	0.0549		-0.9577	0.0099
	Fatigue group: fatigued	-0.0170	0.1161	-0.1460	0.8840	0.9299	-0.2455	0.2116
	Scanner: EDI1	0.4485	0.2531	1.7722	0.0774		-0.0495	0.9465
	Scanner: EDI2	0.2092	0.2519	0.8305	0.4069		-0.2865	0.7050
	Scanner: DUN	0.2151	0.2781	0.7735	0.4398		-0.3322	0.7624

	Scanner: GLA	0.1820	0.2438	0.7467	0.4558		-0.2977	0.6618
	Age	-0.4221	0.0505	-8.3614	0.0000		-0.5215	-0.3228
	Sex: female	0.3168	0.1162	2.7275	0.0068		0.0883	0.5454
	WML	-0.1207	0.0509	-2.3692	0.0185		-0.2209	-0.0204
	Depression score	-0.0590	0.0581	-1.0151	0.3109		-0.1733	0.0554
GM supramarginal	Intercept	-0.1938	0.2571	-0.7537	0.4516		-0.6997	0.3121
	Fatigue group: fatigued	0.0234	0.1214	0.1926	0.8474	0.9245	-0.2156	0.2624
	Scanner: EDI1	0.4562	0.2646	1.7240	0.0857		-0.0645	0.9769
	Scanner: EDI2	-0.0040	0.2634	-0.0154	0.9878		-0.5224	0.5143
	Scanner: DUN	-0.0333	0.2908	-0.1145	0.9089		-0.6056	0.5390
	Scanner: GLA	0.1041	0.2549	0.4081	0.6835		-0.3976	0.6057
	Age	-0.3515	0.0528	-6.6586	0.0000		-0.4554	-0.2476
	Sex: female	0.0591	0.1215	0.4861	0.6272		-0.1800	0.2981
	WML	-0.1098	0.0533	-2.0616	0.0401		-0.2146	-0.0050
	Depression score	-0.0109	0.0608	-0.1796	0.8576		-0.1305	0.1086
GM temporal pole	Intercept	-0.1991	0.2697	-0.7384	0.4609		-0.7298	0.3316
	Fatigue group: fatigued	0.0588	0.1274	0.4612	0.6450	0.8738	-0.1919	0.3094
	Scanner: EDI1	0.0160	0.2776	0.0577	0.9540		-0.5302	0.5622
	Scanner: EDI2	-0.1834	0.2763	-0.6638	0.5073		-0.7272	0.3603
	Scanner: DUN	-0.3753	0.3051	-1.2304	0.2195		-0.9756	0.2250
	Scanner: GLA	-0.1687	0.2674	-0.6307	0.5287		-0.6949	0.3576
	Age	-0.0591	0.0554	-1.0666	0.2870		-0.1680	0.0499
	Sex: female	0.4223	0.1274	3.3142	0.0010		0.1715	0.6730
	WML	-0.1611	0.0559	-2.8837	0.0042		-0.2710	-0.0512
	Depression score	-0.1149	0.0637	-1.8036	0.0723		-0.2403	0.0105
GM transverse temporal	Intercept	-0.2903	0.2664	-1.0898	0.2766		-0.8146	0.2339
	Fatigue group: fatigued	0.2403	0.1258	1.9095	0.0571	0.7776	-0.0073	0.4879
	Scanner: EDI1	0.0292	0.2742	0.1064	0.9153		-0.5104	0.5688
	Scanner: EDI2	-0.0576	0.2730	-0.2109	0.8331		-0.5947	0.4796
	Scanner: DUN	0.0974	0.3013	0.3233	0.7467		-0.4956	0.6904
	Scanner: GLA	-0.2217	0.2642	-0.8391	0.4021		-0.7415	0.2982
	Age	-0.2594	0.0547	-4.7427	0.0000		-0.3671	-0.1518
	Sex: female	0.3497	0.1259	2.7781	0.0058		0.1020	0.5974
	WML	-0.0766	0.0552	-1.3886	0.1660		-0.1852	0.0320
	Depression score	-0.0680	0.0630	-1.0806	0.2808		-0.1919	0.0559
NAWM banks sts	Intercept	-0.5134	0.2668	-1.9243	0.0553		-1.0385	0.0116
	Fatigue group: fatigued	-0.2149	0.1260	-1.7053	0.0892	0.7776	-0.4629	0.0331
	Scanner: EDI1	0.3436	0.2746	1.2511	0.2119		-0.1968	0.8840
	Scanner: EDI2	0.4191	0.2734	1.5328	0.1264		-0.1189	0.9570
	Scanner: DUN	0.5100	0.3018	1.6898	0.0921		-0.0839	1.1039
	Scanner: GLA	0.1969	0.2646	0.7440	0.4575		-0.3238	0.7175
	Age	-0.0700	0.0548	-1.2783	0.2021		-0.1778	0.0378
	Sex: female	0.4054	0.1261	3.2162	0.0014		0.1574	0.6535
	WML	-0.2167	0.0553	-3.9202	0.0001		-0.3254	-0.1079

	Depression score	0.0616	0.0630	0.9768	0.3294		-0.0625	0.1857
NAWM caudal anterior cingulate	Intercept	-0.5455	0.2478	-2.2013	0.0285		-1.0331	-0.0579
	Fatigue group: fatigued	0.1051	0.1171	0.8979	0.3699	0.8738	-0.1252	0.3355
	Scanner: EDI1	0.6646	0.2551	2.6057	0.0096		0.1627	1.1665
	Scanner: EDI2	-0.1550	0.2539	-0.6104	0.5420		-0.6546	0.3447
	Scanner: DUN	0.7519	0.2803	2.6823	0.0077		0.2003	1.3035
	Scanner: GLA	0.0749	0.2457	0.3046	0.7609		-0.4087	0.5584
	Age	-0.0631	0.0509	-1.2403	0.2158		-0.1632	0.0370
	Sex: female	0.3634	0.1171	3.1037	0.0021		0.1330	0.5938
	WML	-0.3168	0.0513	-6.1721	0.0000		-0.4178	-0.2158
	Depression score	-0.0876	0.0586	-1.4960	0.1357		-0.2028	0.0276
NAWM caudal middle frontal	Intercept	-0.0787	0.2582	-0.3048	0.7608		-0.5867	0.4293
	Fatigue group: fatigued	0.0332	0.1219	0.2724	0.7855	0.8917	-0.2068	0.2732
	Scanner: EDI1	0.0491	0.2657	0.1849	0.8535		-0.4738	0.5720
	Scanner: EDI2	-0.7948	0.2645	-3.0047	0.0029		-1.3153	-0.2743
	Scanner: DUN	-0.2342	0.2920	-0.8022	0.4231		-0.8089	0.3404
	Scanner: GLA	-0.2733	0.2560	-1.0675	0.2866		-0.7770	0.2305
	Age	0.0008	0.0530	0.0154	0.9877		-0.1035	0.1051
	Sex: female	0.4902	0.1220	4.0189	0.0001		0.2502	0.7302
	WML	-0.1977	0.0535	-3.6966	0.0003		-0.3029	-0.0925
	Depression score	-0.0900	0.0610	-1.4751	0.1412		-0.2100	0.0301
NAWM cuneus	Intercept	0.3576	0.2768	1.2917	0.1974		-0.1872	0.9024
	Fatigue group: fatigued	0.1173	0.1308	0.8969	0.3705	0.8738	-0.1401	0.3746
	Scanner: EDI1	-0.1359	0.2850	-0.4769	0.6338		-0.6967	0.4248
	Scanner: EDI2	-0.5180	0.2837	-1.8261	0.0688		-1.0762	0.0402
	Scanner: DUN	-0.3556	0.3132	-1.1357	0.2570		-0.9719	0.2606
	Scanner: GLA	-0.2469	0.2745	-0.8995	0.3691		-0.7872	0.2933
	Age	0.0693	0.0568	1.2198	0.2235		-0.0425	0.1812
	Sex: female	-0.1676	0.1308	-1.2816	0.2010		-0.4250	0.0898
	WML	-0.1231	0.0573	-2.1464	0.0326		-0.2360	-0.0102
	Depression score	-0.0301	0.0654	-0.4606	0.6454		-0.1589	0.0986
NAWM entorhinal	Intercept	-0.3365	0.2731	-1.2323	0.2188		-0.8738	0.2008
	Fatigue group: fatigued	-0.1413	0.1290	-1.0956	0.2741	0.8738	-0.3951	0.1125
	Scanner: EDI1	0.6535	0.2811	2.3252	0.0207		0.1004	1.2066
	Scanner: EDI2	0.2175	0.2798	0.7773	0.4376		-0.3331	0.7680
	Scanner: DUN	0.6622	0.3089	2.1438	0.0328		0.0543	1.2700
	Scanner: GLA	0.3467	0.2708	1.2804	0.2014		-0.1861	0.8795
	Age	0.0057	0.0561	0.1023	0.9186		-0.1046	0.1161
	Sex: female	-0.0043	0.1290	-0.0331	0.9736		-0.2581	0.2496
	WML	-0.2095	0.0566	-3.7041	0.0003		-0.3208	-0.0982
	Depression score	0.0395	0.0645	0.6119	0.5411		-0.0875	0.1665
NAWM frontal pole	Intercept	0.1876	0.2726	0.6883	0.4918		-0.3488	0.7240
	Fatigue group: fatigued	0.0433	0.1288	0.3366	0.7366	0.8917	-0.2100	0.2967
	Scanner: EDI1	-0.2658	0.2806	-0.9473	0.3442		-0.8179	0.2863

	Scanner: EDI2	-0.6372	0.2793	-2.2814	0.0232		-1.1869	-0.0876
	Scanner: DUN	-0.7309	0.3084	-2.3702	0.0184		-1.3376	-0.1241
	Scanner: GLA	-0.1573	0.2703	-0.5820	0.5610		-0.6893	0.3746
	Age	0.0320	0.0560	0.5722	0.5676		-0.0781	0.1422
	Sex: female	0.1882	0.1288	1.4615	0.1449		-0.0652	0.4417
	WML	-0.1182	0.0565	-2.0937	0.0371		-0.2294	-0.0071
	Depression score	0.0040	0.0644	0.0618	0.9507		-0.1228	0.1307
NAWM fusiform	Intercept	-0.5285	0.2576	-2.0518	0.0411		-1.0354	-0.0216
	Fatigue group: fatigued	0.0693	0.1217	0.5694	0.5695	0.8738	-0.1701	0.3087
	Scanner: EDI1	0.4931	0.2651	1.8597	0.0639		-0.0287	1.0148
	Scanner: EDI2	0.1364	0.2639	0.5170	0.6055		-0.3829	0.6558
	Scanner: DUN	0.2429	0.2914	0.8338	0.4050		-0.3304	0.8163
	Scanner: GLA	0.3519	0.2554	1.3779	0.1693		-0.1507	0.8546
	Age	-0.0669	0.0529	-1.2651	0.2068		-0.1710	0.0372
	Sex: female	0.2558	0.1217	2.1021	0.0364		0.0163	0.4953
	WML	-0.3617	0.0534	-6.7793	0.0000		-0.4667	-0.2567
	Depression score	-0.0477	0.0609	-0.7834	0.4340		-0.1675	0.0721
NAWM inferior parietal	Intercept	-0.2275	0.2699	-0.8429	0.3999		-0.7587	0.3036
	Fatigue group: fatigued	-0.1513	0.1275	-1.1867	0.2363	0.8738	-0.4022	0.0996
	Scanner: EDI1	0.5410	0.2778	1.9472	0.0524		-0.0057	1.0877
	Scanner: EDI2	0.1746	0.2766	0.6311	0.5284		-0.3697	0.7188
	Scanner: DUN	0.3195	0.3053	1.0462	0.2963		-0.2814	0.9203
	Scanner: GLA	0.1012	0.2677	0.3779	0.7058		-0.4256	0.6279
	Age	0.0155	0.0554	0.2801	0.7796		-0.0935	0.1246
	Sex: female	0.0789	0.1275	0.6189	0.5365		-0.1720	0.3299
	WML	-0.2470	0.0559	-4.4165	0.0000		-0.3570	-0.1369
	Depression score	0.0183	0.0638	0.2869	0.7744		-0.1072	0.1438
NAWM inferior temporal	Intercept	-0.0372	0.2625	-0.1416	0.8875		-0.5536	0.4793
	Fatigue group: fatigued	-0.0641	0.1240	-0.5173	0.6053	0.8738	-0.3081	0.1798
	Scanner: EDI1	0.5270	0.2701	1.9510	0.0520		-0.0046	1.0586
	Scanner: EDI2	-0.2206	0.2689	-0.8203	0.4127		-0.7498	0.3086
	Scanner: DUN	0.0653	0.2969	0.2201	0.8260		-0.5189	0.6495
	Scanner: GLA	0.0538	0.2603	0.2066	0.8365		-0.4584	0.5659
	Age	-0.0600	0.0539	-1.1126	0.2667		-0.1660	0.0461
	Sex: female	-0.0394	0.1240	-0.3173	0.7512		-0.2834	0.2047
	WML	-0.2773	0.0544	-5.1010	0.0000		-0.3843	-0.1703
	Depression score	0.0061	0.0620	0.0977	0.9222		-0.1160	0.1281
NAWM insula	Intercept	0.1154	0.2526	0.4569	0.6481		-0.3817	0.6125
	Fatigue group: fatigued	0.0649	0.1193	0.5437	0.5870	0.8738	-0.1699	0.2997
	Scanner: EDI1	0.2257	0.2600	0.8682	0.3860		-0.2859	0.7374
	Scanner: EDI2	-0.6244	0.2588	-2.4125	0.0164		-1.1338	-0.1151
	Scanner: DUN	-0.5077	0.2857	-1.7769	0.0766		-1.0700	0.0546
	Scanner: GLA	-0.5266	0.2505	-2.1024	0.0363		-1.0196	-0.0337
	Age	0.0535	0.0519	1.0321	0.3028		-0.0485	0.1556

	Sex: female	0.2752	0.1194	2.3059	0.0218		0.0404	0.5101
	WML	-0.2673	0.0523	-5.1080	0.0000		-0.3703	-0.1643
	Depression score	-0.0467	0.0597	-0.7821	0.4348		-0.1641	0.0708
NAWM isthmus cingulate	Intercept	0.0553	0.2582	0.2143	0.8304		-0.4528	0.5634
	Fatigue group: fatigued	0.2985	0.1220	2.4472	0.0150	0.7776	0.0585	0.5385
	Scanner: EDI1	-0.2393	0.2658	-0.9005	0.3685		-0.7623	0.2836
	Scanner: EDI2	-0.5552	0.2646	-2.0988	0.0367		-1.0759	-0.0346
	Scanner: DUN	-0.4347	0.2921	-1.4884	0.1377		-1.0095	0.1400
	Scanner: GLA	-0.3957	0.2560	-1.5454	0.1233		-0.8995	0.1081
	Age	0.0858	0.0530	1.6177	0.1068		-0.0186	0.1901
	Sex: female	0.2557	0.1220	2.0958	0.0369		0.0156	0.4957
	WML	-0.3428	0.0535	-6.4084	0.0000		-0.4480	-0.2375
	Depression score	-0.0457	0.0610	-0.7494	0.4542		-0.1658	0.0743
NAWM lateral occipital	Intercept	-0.1779	0.2747	-0.6476	0.5177		-0.7184	0.3626
	Fatigue group: fatigued	0.0505	0.1298	0.3888	0.6977	0.8917	-0.2049	0.3058
	Scanner: EDI1	0.5292	0.2827	1.8716	0.0622		-0.0272	1.0855
	Scanner: EDI2	0.0119	0.2815	0.0423	0.9663		-0.5419	0.5657
	Scanner: DUN	0.5099	0.3107	1.6410	0.1018		-0.1016	1.1213
	Scanner: GLA	0.3567	0.2724	1.3094	0.1914		-0.1793	0.8927
	Age	-0.0946	0.0564	-1.6777	0.0944		-0.2056	0.0164
	Sex: female	-0.2130	0.1298	-1.6413	0.1018		-0.4684	0.0424
	WML	-0.0925	0.0569	-1.6251	0.1052		-0.2044	0.0195
	Depression score	0.0028	0.0649	0.0434	0.9654		-0.1249	0.1305
NAWM lateral orbitofrontal	Intercept	-0.3924	0.2622	-1.4965	0.1356		-0.9083	0.1236
	Fatigue group: fatigued	0.2672	0.1239	2.1578	0.0317	0.7776	0.0235	0.5110
	Scanner: EDI1	0.4506	0.2699	1.6698	0.0960		-0.0804	0.9817
	Scanner: EDI2	-0.1151	0.2686	-0.4283	0.6688		-0.6437	0.4136
	Scanner: DUN	0.1892	0.2966	0.6380	0.5240		-0.3944	0.7728
	Scanner: GLA	-0.0289	0.2600	-0.1112	0.9115		-0.5405	0.4827
	Age	0.0495	0.0538	0.9201	0.3583		-0.0564	0.1555
	Sex: female	0.2481	0.1239	2.0029	0.0461		0.0043	0.4919
	WML	-0.2671	0.0543	-4.9182	0.0000		-0.3740	-0.1602
	Depression score	0.0005	0.0620	0.0080	0.9936		-0.1214	0.1224
NAWM lingual	Intercept	-0.3503	0.2686	-1.3039	0.1932		-0.8789	0.1783
	Fatigue group: fatigued	0.0972	0.1269	0.7659	0.4443	0.8738	-0.1525	0.3469
	Scanner: EDI1	0.3695	0.2765	1.3361	0.1825		-0.1747	0.9136
	Scanner: EDI2	-0.0456	0.2753	-0.1658	0.8684		-0.5873	0.4960
	Scanner: DUN	0.0934	0.3039	0.3074	0.7588		-0.5046	0.6914
	Scanner: GLA	0.1806	0.2664	0.6780	0.4983		-0.3436	0.7049
	Age	-0.0352	0.0552	-0.6381	0.5239		-0.1437	0.0734
	Sex: female	0.2046	0.1269	1.6123	0.1079		-0.0451	0.4544
	WML	-0.2637	0.0557	-4.7392	0.0000		-0.3733	-0.1542
	Depression score	0.0226	0.0635	0.3554	0.7225		-0.1024	0.1475
NAW	Intercept	0.2990	0.2653	1.1271	0.2606		-0.2230	0.8211

	Fatigue group: fatigued	0.2483	0.1253	1.9816	0.0484	0.7776	0.0017	0.4949
	Scanner: EDI1	-0.0566	0.2731	-0.2074	0.8358		-0.5940	0.4807
	Scanner: EDI2	-0.5659	0.2718	-2.0818	0.0382		-1.1008	-0.0310
	Scanner: DUN	-0.5887	0.3001	-1.9619	0.0507		-1.1793	0.0018
	Scanner: GLA	-0.3351	0.2631	-1.2738	0.2037		-0.8528	0.1826
	Age	0.0827	0.0545	1.5174	0.1302		-0.0245	0.1899
	Sex: female	-0.0991	0.1253	-0.7908	0.4297		-0.3458	0.1475
	WML	-0.2480	0.0550	-4.5125	0.0000		-0.3561	-0.1398
	Depression score	-0.1030	0.0627	-1.6437	0.1013		-0.2264	0.0203
NAWM middle temporal	Intercept	0.0134	0.2734	0.0491	0.9609		-0.5245	0.5514
	Fatigue group: fatigued	-0.1278	0.1291	-0.9898	0.3231	0.8738	-0.3819	0.1263
	Scanner: EDI1	0.1115	0.2814	0.3961	0.6923		-0.4422	0.6652
	Scanner: EDI2	-0.3161	0.2801	-1.1285	0.2600		-0.8673	0.2351
	Scanner: DUN	-0.1351	0.3092	-0.4368	0.6626		-0.7436	0.4734
	Scanner: GLA	-0.1441	0.2711	-0.5316	0.5954		-0.6776	0.3893
	Age	-0.0774	0.0561	-1.3796	0.1687		-0.1879	0.0330
	Sex: female	0.2170	0.1292	1.6802	0.0940		-0.0372	0.4712
	WML	-0.1689	0.0566	-2.9833	0.0031		-0.2804	-0.0575
	Depression score	0.0018	0.0646	0.0273	0.9782		-0.1254	0.1289
NAWM paracentral	Intercept	0.1341	0.2589	0.5179	0.6049		-0.3753	0.6435
	Fatigue group: fatigued	0.0762	0.1223	0.6230	0.5337	0.8738	-0.1644	0.3168
	Scanner: EDI1	-0.2095	0.2665	-0.7863	0.4323		-0.7339	0.3148
	Scanner: EDI2	-0.7335	0.2652	-2.7655	0.0060		-1.2555	-0.2116
	Scanner: DUN	-0.3540	0.2928	-1.2088	0.2277		-0.9302	0.2223
	Scanner: GLA	-0.3151	0.2567	-1.2274	0.2206		-0.8202	0.1901
	Age	-0.0551	0.0532	-1.0363	0.3009		-0.1597	0.0495
	Sex: female	0.2787	0.1223	2.2784	0.0234		0.0380	0.5194
	WML	-0.3285	0.0536	-6.1263	0.0000		-0.4341	-0.2230
	Depression score	-0.0089	0.0612	-0.1449	0.8849		-0.1292	0.1115
NAWM parahippocampal	Intercept	-0.3184	0.2592	-1.2283	0.2203		-0.8286	0.1917
	Fatigue group: fatigued	0.0972	0.1225	0.7941	0.4278	0.8738	-0.1437	0.3382
	Scanner: EDI1	0.1885	0.2668	0.7065	0.4804		-0.3366	0.7136
	Scanner: EDI2	-0.0706	0.2656	-0.2658	0.7905		-0.5933	0.4521
	Scanner: DUN	-0.1063	0.2933	-0.3625	0.7172		-0.6834	0.4708
	Scanner: GLA	0.1064	0.2571	0.4140	0.6792		-0.3995	0.6123
	Age	0.1187	0.0532	2.2299	0.0265		0.0139	0.2235
	Sex: female	0.2914	0.1225	2.3789	0.0180		0.0504	0.5324
	WML	-0.3526	0.0537	-6.5657	0.0000		-0.4583	-0.2469
	Depression score	-0.0311	0.0613	-0.5070	0.6125		-0.1516	0.0895
NAWM pars opercularis	Intercept	0.0909	0.2689	0.3378	0.7357		-0.4384	0.6201
	Fatigue group: fatigued	0.0183	0.1270	0.1440	0.8856	0.9299	-0.2317	0.2683
	Scanner: EDI1	0.1008	0.2768	0.3643	0.7159		-0.4439	0.6456
	Scanner: EDI2	-0.5147	0.2756	-1.8679	0.0627		-1.0570	0.0275
	Scanner: DUN	-0.2449	0.3042	-0.8049	0.4215		-0.8435	0.3538

	Scanner: GLA	-0.2006	0.2667	-0.7520	0.4526		-0.7254	0.3242
	Age	-0.1139	0.0552	-2.0622	0.0400		-0.2226	-0.0052
	Sex: female	0.1352	0.1271	1.0641	0.2881		-0.1148	0.3853
	WML	-0.1978	0.0557	-3.5498	0.0004		-0.3074	-0.0881
	Depression score	-0.0089	0.0636	-0.1396	0.8890		-0.1339	0.1162
NAWM pars orbitalis	Intercept	0.3024	0.2748	1.1006	0.2720		-0.2383	0.8431
	Fatigue group: fatigued	-0.0228	0.1298	-0.1753	0.8610	0.9272	-0.2781	0.2326
	Scanner: EDI1	0.2096	0.2828	0.7412	0.4591		-0.3469	0.7661
	Scanner: EDI2	-0.3855	0.2815	-1.3694	0.1719		-0.9395	0.1685
	Scanner: DUN	-0.1826	0.3108	-0.5874	0.5574		-0.7942	0.4290
	Scanner: GLA	-0.0692	0.2725	-0.2540	0.7997		-0.6054	0.4670
	Age	0.0261	0.0564	0.4624	0.6442		-0.0849	0.1371
	Sex: female	-0.2708	0.1298	-2.0863	0.0378		-0.5263	-0.0154
	WML	-0.0826	0.0569	-1.4505	0.1480		-0.1946	0.0294
	Depression score	0.0601	0.0649	0.9260	0.3552		-0.0676	0.1879
NAWM pars triangularis	Intercept	-0.0619	0.2769	-0.2234	0.8234		-0.6067	0.4830
	Fatigue group: fatigued	0.1333	0.1308	1.0191	0.3090	0.8738	-0.1241	0.3907
	Scanner: EDI1	0.3828	0.2850	1.3432	0.1802		-0.1780	0.9437
	Scanner: EDI2	-0.1837	0.2837	-0.6475	0.5178		-0.7420	0.3746
	Scanner: DUN	0.0914	0.3132	0.2917	0.7707		-0.5250	0.7077
	Scanner: GLA	0.0731	0.2746	0.2662	0.7902		-0.4672	0.6134
	Age	-0.0036	0.0569	-0.0625	0.9502		-0.1154	0.1083
	Sex: female	-0.1073	0.1308	-0.8201	0.4128		-0.3647	0.1502
	WML	-0.0929	0.0574	-1.6196	0.1064		-0.2058	0.0200
	Depression score	-0.0408	0.0654	-0.6233	0.5336		-0.1695	0.0880
NAWM pericalcarine	Intercept	-0.0818	0.2683	-0.3050	0.7606		-0.6098	0.4461
	Fatigue group: fatigued	0.0603	0.1267	0.4759	0.6345	0.8738	-0.1891	0.3097
	Scanner: EDI1	0.1540	0.2761	0.5575	0.5776		-0.3895	0.6974
	Scanner: EDI2	-0.2190	0.2749	-0.7967	0.4262		-0.7600	0.3219
	Scanner: DUN	-0.1736	0.3035	-0.5721	0.5677		-0.7708	0.4236
	Scanner: GLA	0.0474	0.2660	0.1783	0.8586		-0.4761	0.5709
	Age	-0.0695	0.0551	-1.2616	0.2081		-0.1779	0.0389
	Sex: female	0.0950	0.1268	0.7492	0.4543		-0.1545	0.3444
	WML	-0.2686	0.0556	-4.8330	0.0000		-0.3780	-0.1592
	Depression score	0.0683	0.0634	1.0773	0.2822		-0.0565	0.1931
NAWM postcentral	Intercept	0.4368	0.2612	1.6723	0.0955		-0.0772	0.9509
	Fatigue group: fatigued	-0.0081	0.1234	-0.0659	0.9475	0.9673	-0.2510	0.2347
	Scanner: EDI1	-0.0110	0.2689	-0.0410	0.9673		-0.5401	0.5181
	Scanner: EDI2	-0.8877	0.2677	-3.3167	0.0010		-1.4145	-0.3610
	Scanner: DUN	-0.6988	0.2955	-2.3650	0.0187		-1.2803	-0.1174
	Scanner: GLA	-0.4857	0.2590	-1.8748	0.0618		-0.9954	0.0241
	Age	0.0598	0.0536	1.1145	0.2659		-0.0458	0.1653
	Sex: female	0.0513	0.1234	0.4154	0.6781		-0.1916	0.2942
	WML	-0.2094	0.0541	-3.8700	0.0001		-0.3159	-0.1029

	Depression score	-0.0215	0.0617	-0.3488	0.7275		-0.1430	0.0999
NAWM posterior cingulate	Intercept	-0.2112	0.2302	-0.9174	0.3597		-0.6643	0.2419
	Fatigue group: fatigued	0.1119	0.1088	1.0291	0.3043	0.8738	-0.1021	0.3259
	Scanner: EDI1	0.3794	0.2370	1.6009	0.1104		-0.0869	0.8457
	Scanner: EDI2	-0.3988	0.2359	-1.6907	0.0919		-0.8631	0.0654
	Scanner: DUN	-0.0062	0.2604	-0.0237	0.9811		-0.5187	0.5063
	Scanner: GLA	-0.0613	0.2283	-0.2686	0.7884		-0.5106	0.3880
	Age	-0.0735	0.0473	-1.5553	0.1209		-0.1666	0.0195
	Sex: female	0.2552	0.1088	2.3457	0.0196		0.0411	0.4692
	WML	-0.5026	0.0477	-10.5382	0.0000		-0.5965	-0.4088
	Depression score	0.0161	0.0544	0.2956	0.7677		-0.0910	0.1231
NAWM precentral	Intercept	0.0829	0.2631	0.3151	0.7529		-0.4349	0.6007
	Fatigue group: fatigued	0.0760	0.1243	0.6117	0.5412	0.8738	-0.1686	0.3206
	Scanner: EDI1	0.1147	0.2708	0.4235	0.6722		-0.4183	0.6477
	Scanner: EDI2	-0.6198	0.2696	-2.2990	0.0222		-1.1504	-0.0893
	Scanner: DUN	-0.1547	0.2976	-0.5198	0.6036		-0.7404	0.4310
	Scanner: GLA	-0.3615	0.2609	-1.3856	0.1669		-0.8750	0.1519
	Age	0.1002	0.0540	1.8550	0.0646		-0.0061	0.2065
	Sex: female	0.2085	0.1243	1.6772	0.0945		-0.0361	0.4532
	WML	-0.2385	0.0545	-4.3755	0.0000		-0.3458	-0.1312
	Depression score	-0.0529	0.0622	-0.8511	0.3954		-0.1753	0.0694
NAWM precuneus	Intercept	0.4720	0.2627	1.7967	0.0734		-0.0449	0.9890
	Fatigue group: fatigued	-0.0770	0.1241	-0.6206	0.5354	0.8738	-0.3212	0.1672
	Scanner: EDI1	-0.2339	0.2704	-0.8651	0.3876		-0.7660	0.2982
	Scanner: EDI2	-0.4287	0.2692	-1.5925	0.1123		-0.9583	0.1010
	Scanner: DUN	-0.4857	0.2972	-1.6346	0.1032		-1.0705	0.0990
	Scanner: GLA	-0.4125	0.2605	-1.5833	0.1144		-0.9251	0.1002
	Age	0.0493	0.0539	0.9139	0.3615		-0.0568	0.1554
	Sex: female	-0.0974	0.1241	-0.7848	0.4332		-0.3417	0.1468
	WML	-0.3545	0.0544	-6.5144	0.0000		-0.4616	-0.2474
	Depression score	0.0507	0.0621	0.8170	0.4146		-0.0714	0.1729
NAWM rostral anterior cingulate	Intercept	0.3241	0.2718	1.1923	0.2341		-0.2108	0.8589
	Fatigue group: fatigued	0.1117	0.1284	0.8701	0.3849	0.8738	-0.1409	0.3643
	Scanner: EDI1	-0.3241	0.2797	-1.1586	0.2475		-0.8746	0.2264
	Scanner: EDI2	-0.8079	0.2785	-2.9012	0.0040		-1.3559	-0.2599
	Scanner: DUN	-0.5521	0.3074	-1.7959	0.0735		-1.1571	0.0528
	Scanner: GLA	-0.4846	0.2695	-1.7979	0.0732		-1.0149	0.0458
	Age	-0.0677	0.0558	-1.2124	0.2263		-0.1775	0.0422
	Sex: female	0.1771	0.1284	1.3792	0.1688		-0.0756	0.4298
	WML	-0.1754	0.0563	-3.1146	0.0020		-0.2861	-0.0646
	Depression score	-0.0245	0.0642	-0.3819	0.7028		-0.1509	0.1019
NAWM rostral middle	Intercept	-0.0756	0.2672	-0.2828	0.7775		-0.6015	0.4503
	Fatigue group: fatigued	0.0362	0.1262	0.2869	0.7744	0.8917	-0.2122	0.2846
	Scanner: EDI1	0.5240	0.2751	1.9049	0.0577		-0.0173	1.0652

	Scanner: EDI2	-0.2558	0.2738	-0.9343	0.3509		-0.7947	0.2830
	Scanner: DUN	0.3003	0.3023	0.9934	0.3213		-0.2946	0.8952
	Scanner: GLA	0.2888	0.2650	1.0897	0.2767		-0.2327	0.8103
	Age	-0.0571	0.0549	-1.0413	0.2986		-0.1651	0.0508
	Sex: female	-0.1910	0.1263	-1.5124	0.1315		-0.4394	0.0575
	WML	-0.1829	0.0554	-3.3040	0.0011		-0.2918	-0.0740
	Depression score	-0.0215	0.0631	-0.3399	0.7342		-0.1457	0.1028
NAWM superior frontal	Intercept	0.3154	0.2617	1.2055	0.2290		-0.1995	0.8304
	Fatigue group: fatigued	0.1145	0.1236	0.9264	0.3550	0.8738	-0.1287	0.3577
	Scanner: EDI1	0.0091	0.2693	0.0339	0.9730		-0.5209	0.5391
	Scanner: EDI2	-0.9351	0.2681	-3.4877	0.0006		-1.4627	-0.4075
	Scanner: DUN	-0.3804	0.2960	-1.2853	0.1997		-0.9629	0.2020
	Scanner: GLA	-0.3483	0.2595	-1.3424	0.1805		-0.8589	0.1623
	Age	0.0160	0.0537	0.2981	0.7659		-0.0897	0.1217
	Sex: female	0.0313	0.1236	0.2533	0.8002		-0.2120	0.2746
	WML	-0.2028	0.0542	-3.7411	0.0002		-0.3095	-0.0961
	Depression score	-0.0249	0.0618	-0.4031	0.6871		-0.1466	0.0967
NAWM superior parietal	Intercept	-0.1497	0.2668	-0.5610	0.5752		-0.6748	0.3754
	Fatigue group: fatigued	0.0662	0.1260	0.5253	0.5997	0.8738	-0.1818	0.3143
	Scanner: EDI1	0.0971	0.2747	0.3536	0.7239		-0.4434	0.6376
	Scanner: EDI2	-0.2467	0.2734	-0.9022	0.3677		-0.7847	0.2914
	Scanner: DUN	-0.4082	0.3018	-1.3525	0.1772		-1.0022	0.1857
	Scanner: GLA	-0.0117	0.2646	-0.0441	0.9649		-0.5324	0.5091
	Age	0.0181	0.0548	0.3310	0.7408		-0.0897	0.1260
	Sex: female	0.2717	0.1261	2.1548	0.0320		0.0236	0.5198
	WML	-0.2590	0.0553	-4.6855	0.0000		-0.3678	-0.1502
	Depression score	-0.0296	0.0631	-0.4688	0.6396		-0.1536	0.0945
NAWM superior temporal	Intercept	0.2744	0.2720	1.0091	0.3137		-0.2607	0.8096
	Fatigue group: fatigued	0.1670	0.1285	1.2999	0.1946	0.8738	-0.0858	0.4198
	Scanner: EDI1	0.0122	0.2799	0.0437	0.9652		-0.5386	0.5631
	Scanner: EDI2	-0.4363	0.2786	-1.5658	0.1184		-0.9846	0.1120
	Scanner: DUN	-0.1425	0.3076	-0.4632	0.6436		-0.7478	0.4629
	Scanner: GLA	-0.3248	0.2697	-1.2042	0.2294		-0.8554	0.2059
	Age	0.0069	0.0558	0.1244	0.9011		-0.1029	0.1168
	Sex: female	-0.1481	0.1285	-1.1530	0.2498		-0.4010	0.1047
	WML	-0.2137	0.0563	-3.7926	0.0002		-0.3245	-0.1028
	Depression score	-0.0664	0.0643	-1.0329	0.3025		-0.1928	0.0601
NAWM supramarginal	Intercept	0.4278	0.2704	1.5824	0.1146		-0.1042	0.9598
	Fatigue group: fatigued	0.0934	0.1277	0.7317	0.4649	0.8738	-0.1579	0.3447
	Scanner: EDI1	-0.0948	0.2783	-0.3406	0.7337		-0.6424	0.4528
	Scanner: EDI2	-0.4474	0.2770	-1.6150	0.1073		-0.9925	0.0977
	Scanner: DUN	-0.4762	0.3058	-1.5571	0.1205		-1.0780	0.1256
	Scanner: GLA	-0.4109	0.2681	-1.5326	0.1264		-0.9384	0.1167
	Age	0.0199	0.0555	0.3591	0.7198		-0.0893	0.1292

	Sex: female	-0.1799	0.1277	-1.4088	0.1599		-0.4313	0.0714
	WML	-0.2440	0.0560	-4.3569	0.0000		-0.3542	-0.1338
	Depression score	0.0274	0.0639	0.4286	0.6685		-0.0983	0.1531
NAWM temporal pole	Intercept	0.2939	0.2628	1.1184	0.2643		-0.2233	0.8111
	Fatigue group: fatigued	-0.0289	0.1242	-0.2329	0.8160	0.9019	-0.2732	0.2154
	Scanner: EDI1	0.2454	0.2705	0.9070	0.3651		-0.2870	0.7777
	Scanner: EDI2	-0.4768	0.2693	-1.7705	0.0777		-1.0067	0.0531
	Scanner: DUN	-0.6201	0.2973	-2.0858	0.0378		-1.2052	-0.0351
	Scanner: GLA	-0.2134	0.2606	-0.8189	0.4135		-0.7263	0.2994
	Age	0.0841	0.0540	1.5590	0.1200		-0.0221	0.1903
	Sex: female	-0.1013	0.1242	-0.8161	0.4151		-0.3457	0.1430
	WML	-0.2234	0.0544	-4.1031	0.0001		-0.3305	-0.1163
	Depression score	0.0099	0.0621	0.1597	0.8732		-0.1123	0.1321
NAWM transverse temporal	Intercept	-0.4198	0.2772	-1.5145	0.1310		-0.9652	0.1257
	Fatigue group: fatigued	0.1462	0.1309	1.1168	0.2649	0.8738	-0.1114	0.4039
	Scanner: EDI1	0.0548	0.2853	0.1923	0.8477		-0.5065	0.6162
	Scanner: EDI2	0.0181	0.2840	0.0637	0.9493		-0.5408	0.5769
	Scanner: DUN	0.0389	0.3135	0.1239	0.9015		-0.5781	0.6558
	Scanner: GLA	0.0958	0.2748	0.3486	0.7276		-0.4450	0.6367
	Age	0.0406	0.0569	0.7132	0.4763		-0.0714	0.1526
	Sex: female	0.3940	0.1310	3.0086	0.0028		0.1363	0.6517
	WML	-0.0902	0.0574	-1.5705	0.1173		-0.2032	0.0228
	Depression score	0.0096	0.0655	0.1463	0.8838		-0.1193	0.1385
WML	Intercept	-0.1175	0.2768	-0.4244	0.6716		-0.6621	0.4272
	Fatigue group: fatigued	-0.0728	0.1307	-0.5568	0.5781	0.8738	-0.3300	0.1844
	Scanner: EDI1	0.2807	0.2845	0.9867	0.3246		-0.2791	0.8406
	Scanner: EDI2	0.1439	0.2836	0.5073	0.6123		-0.4141	0.7019
	Scanner: DUN	0.6746	0.3108	2.1706	0.0307		0.0630	1.2862
	Scanner: GLA	0.3109	0.2740	1.1348	0.2574		-0.2282	0.8500
	Age	0.0881	0.0566	1.5554	0.1209		-0.0234	0.1995
	Sex: female	-0.1854	0.1304	-1.4223	0.1560		-0.4420	0.0711
	Depression score	0.0800	0.0653	1.2263	0.2210		-0.0484	0.2085
Whole-brain	Intercept	-0.4366	0.2335	-1.8697	0.0625		-0.8961	0.0229
	Fatigue group: fatigued	0.0323	0.1103	0.2925	0.7701	0.8917	-0.1847	0.2493
	Scanner: EDI1	0.7996	0.2400	3.3313	0.0010		0.3273	1.2719
	Scanner: EDI2	-0.3585	0.2392	-1.4987	0.1350		-0.8293	0.1122
	Scanner: DUN	0.0651	0.2622	0.2483	0.8040		-0.4508	0.5810
	Scanner: GLA	0.1649	0.2311	0.7132	0.4762		-0.2900	0.6197
	Age	-0.3687	0.0478	-7.7173	0.0000		-0.4627	-0.2747
	Sex: female	0.3382	0.1100	3.0747	0.0023		0.1218	0.5546
	Depression score	-0.0775	0.0551	-1.4070	0.1605		-0.1858	0.0309
SCCSA-C2-3	Intercept	0.4154	0.2604	1.5949	0.1117		-0.0970	0.9278
	Fatigue group: fatigued	0.0199	0.1244	0.1602	0.8728	0.8728	-0.2248	0.2647
	Scanner: EDI1	-0.3875	0.2653	-1.4605	0.1451		-0.9095	0.1345

	Scanner: EDI2	-0.4037	0.2631	-1.5342	0.1260		-0.9214	0.1140
	Scanner: DUN	0.1460	0.2887	0.5058	0.6134		-0.4221	0.7141
	Scanner: GLA	-0.1949	0.2549	-0.7646	0.4451		-0.6966	0.3067
	Age	-0.2264	0.0533	-4.2488	0.0000		-0.3312	-0.1216
	Sex: female	-0.2485	0.1231	-2.0189	0.0443		-0.4906	-0.0063
	Depression score	-0.1610	0.0622	-2.5875	0.0101		-0.2835	-0.0386

RRMS=relapsing-remitting multiple sclerosis, FDR=false discovery rate, $B_{standardised}$=standardised beta value, SE=standard error, CI=confidence interval for beta value, w0=baseline, w1=1-year follow-up, GM=grey matter, NAWM=normal-appearing white matter, sts=superior temporal sulcus, WML=white matter lesion, ED1=Edinburgh scanner 1, EDI2=Edinburgh scanner 2, DUN=Dundee, GLA=Glasgow, SCCSA-C2-3=spinal cord cross-sectional area cervical levels 2 and 3, PHQ-9=patient health questionnaire, FSS=fatigue severity scale

Supplementary Table 4. Results for 1-year follow-up brain tissue volumes, WML volumes and SCCSA-C2-3 differences between RRMS participants with and without fatigue (based on FSS score). This was assessed using multiple linear regression models, with fatigue group as regressor of interest and MRI scanner, age_{w0}, sex, DMT intake at w1, WML_{w0} (not for WML, whole-brain and SCCSA) and depression score (based on PHQ-9) as control variables. P-values for the regressor of interest were corrected for multiple comparisons using the FDR (q<.05).

		$B_{standardised}$	SD	t-value	$p_{uncorrected}$	p_{FDR}	CI 2.5%	CI 97.5%
Brainstem	Intercept	-0.1672	0.2604	-0.6422	0.5212		-0.6796	0.3452
	Fatigue group: fatigued	-0.0059	0.1167	-0.0501	0.9600	0.9818	-0.2355	0.2238
	Scanner: EDI1	0.4262	0.2715	1.5695	0.1176		-0.1082	0.9605
	Scanner: EDI2	-0.3663	0.2716	-1.3490	0.1783		-0.9008	0.1681
	Scanner: DUN	-0.0293	0.3019	-0.0971	0.9227		-0.6233	0.5647
	Scanner: GLA	-0.2199	0.2693	-0.8165	0.4149		-0.7500	0.3101
	Age	-0.1003	0.0554	-1.8102	0.0713		-0.2092	0.0087
	Sex: female	0.2286	0.1232	1.8550	0.0646		-0.0139	0.4711
	WML	-0.1857	0.0542	-3.4240	0.0007		-0.2924	-0.0790
	Depression score	-0.1052	0.0576	-1.8274	0.0686		-0.2185	0.0081
	DMT w1: yes	0.1014	0.1323	0.7662	0.4442		-0.1590	0.3618
Accumbens	Intercept	-0.4547	0.2465	-1.8448	0.0660		-0.9398	0.0303
	Fatigue group: fatigued	0.2109	0.1105	1.9089	0.0572	0.9603	-0.0065	0.4284
	Scanner: EDI1	0.0341	0.2571	0.1327	0.8946		-0.4717	0.5399
	Scanner: EDI2	0.2272	0.2571	0.8836	0.3776		-0.2787	0.7331
	Scanner: DUN	0.1997	0.2858	0.6989	0.4851		-0.3626	0.7621
	Scanner: GLA	0.1898	0.2550	0.7445	0.4571		-0.3119	0.6916
	Age	-0.1840	0.0524	-3.5101	0.0005		-0.2872	-0.0809
	Sex: female	0.3709	0.1167	3.1792	0.0016		0.1413	0.6004
	WML	-0.3719	0.0513	-7.2432	0.0000		-0.4729	-0.2708
	Depression score	-0.0569	0.0545	-1.0437	0.2975		-0.1641	0.0504
	DMT w1: yes	-0.1015	0.1253	-0.8105	0.4183		-0.3481	0.1450
Amygdala	Intercept	0.1014	0.2616	0.3878	0.6985		-0.4134	0.6162
	Fatigue group: fatigued	0.1154	0.1173	0.9837	0.3260	0.9603	-0.1154	0.3461
	Scanner: EDI1	-0.5279	0.2728	-1.9349	0.0539		-1.0647	0.0090
	Scanner: EDI2	-0.1198	0.2729	-0.4391	0.6609		-0.6568	0.4171
	Scanner: DUN	-0.2719	0.3033	-0.8964	0.3707		-0.8687	0.3250
	Scanner: GLA	-0.1996	0.2706	-0.7375	0.4614		-0.7321	0.3330
	Age	-0.2526	0.0556	-4.5389	0.0000		-0.3621	-0.1431
	Sex: female	0.2372	0.1238	1.9158	0.0563		-0.0064	0.4808
	WML	-0.1633	0.0545	-2.9970	0.0030		-0.2705	-0.0561
	Depression score	-0.0662	0.0578	-1.1441	0.2535		-0.1800	0.0476
	DMT w1: yes	-0.1063	0.1330	-0.7996	0.4245		-0.3680	0.1553
Caudate	Intercept	-0.1641	0.2334	-0.7031	0.4825		-0.6234	0.2952
	Fatigue group: fatigued	0.0469	0.1046	0.4485	0.6541	0.9603	-0.1590	0.2528
	Scanner: EDI1	0.5005	0.2434	2.0563	0.0406		0.0215	0.9795
	Scanner: EDI2	-0.5109	0.2434	-2.0986	0.0367		-0.9900	-0.0318
	Scanner: DUN	-0.7266	0.2706	-2.6852	0.0076		-1.2592	-0.1941
	Scanner: GLA	-0.4919	0.2415	-2.0372	0.0425		-0.9670	-0.0167
	Age	-0.0891	0.0496	-1.7946	0.0737		-0.1868	0.0086

	Sex: female	0.4523	0.1105	4.0947	0.0001		0.2349	0.6697
	WML	-0.2670	0.0486	-5.4912	0.0000		-0.3626	-0.1713
	Depression score	0.0310	0.0516	0.6010	0.5483		-0.0705	0.1326
	DMT w1: yes	0.1039	0.1186	0.8759	0.3818		-0.1295	0.3373
GM cerebellar	Intercept	-0.3595	0.2528	-1.4221	0.1560		-0.8568	0.1379
	Fatigue group: fatigued	-0.2177	0.1133	-1.9215	0.0556	0.9603	-0.4407	0.0053
	Scanner: EDI1	0.4117	0.2636	1.5619	0.1194		-0.1070	0.9304
	Scanner: EDI2	-0.2658	0.2636	-1.0084	0.3141		-0.7846	0.2529
	Scanner: DUN	0.2486	0.2930	0.8485	0.3968		-0.3280	0.8253
	Scanner: GLA	0.2244	0.2615	0.8583	0.3914		-0.2901	0.7390
	Age	-0.3008	0.0538	-5.5940	0.0000		-0.4066	-0.1950
	Sex: female	0.3605	0.1196	3.0133	0.0028		0.1251	0.5959
	WML	-0.0363	0.0526	-0.6887	0.4915		-0.1399	0.0673
	Depression score	-0.0317	0.0559	-0.5678	0.5706		-0.1417	0.0782
	DMT w1: yes	0.0587	0.1285	0.4571	0.6479		-0.1941	0.3115
GM cerebral	Intercept	-0.5963	0.2319	-2.5713	0.0106		-1.0527	-0.1399
	Fatigue group: fatigued	0.0458	0.1040	0.4401	0.6602	0.9603	-0.1588	0.2503
	Scanner: EDI1	0.7911	0.2419	3.2708	0.0012		0.3151	1.2670
	Scanner: EDI2	-0.0529	0.2419	-0.2187	0.8270		-0.5289	0.4231
	Scanner: DUN	0.3420	0.2689	1.2718	0.2044		-0.1871	0.8711
	Scanner: GLA	0.4487	0.2399	1.8703	0.0624		-0.0234	0.9208
	Age	-0.4196	0.0493	-8.5067	0.0000		-0.5167	-0.3226
	Sex: female	0.4451	0.1098	4.0553	0.0001		0.2291	0.6611
	WML	-0.1024	0.0483	-2.1204	0.0348		-0.1975	-0.0074
	Depression score	-0.0552	0.0513	-1.0765	0.2826		-0.1561	0.0457
	DMT w1: yes	-0.1836	0.1179	-1.5576	0.1204		-0.4156	0.0484
Hippocampus	Intercept	0.0681	0.2763	0.2466	0.8054		-0.4755	0.6118
	Fatigue group: fatigued	-0.0532	0.1238	-0.4294	0.6679	0.9603	-0.2969	0.1905
	Scanner: EDI1	-0.3279	0.2881	-1.1382	0.2559		-0.8949	0.2390
	Scanner: EDI2	-0.2450	0.2881	-0.8502	0.3959		-0.8120	0.3221
	Scanner: DUN	-0.4312	0.3203	-1.3462	0.1793		-1.0615	0.1991
	Scanner: GLA	-0.3684	0.2858	-1.2892	0.1983		-0.9308	0.1940
	Age	-0.0971	0.0588	-1.6518	0.0996		-0.2127	0.0186
	Sex: female	0.1985	0.1307	1.5181	0.1300		-0.0588	0.4558
	WML	-0.0948	0.0575	-1.6471	0.1006		-0.2080	0.0185
	Depression score	-0.0318	0.0611	-0.5209	0.6028		-0.1520	0.0884
	DMT w1: yes	0.1723	0.1404	1.2270	0.2208		-0.1040	0.4486
NAWM cerebral	Intercept	-0.0350	0.2446	-0.1432	0.8862		-0.5164	0.4464
	Fatigue group: fatigued	0.1139	0.1097	1.0384	0.2999	0.9603	-0.1019	0.3297
	Scanner: EDI1	0.2090	0.2551	0.8193	0.4132		-0.2930	0.7111
	Scanner: EDI2	-0.5146	0.2552	-2.0169	0.0446		-1.0168	-0.0125
	Scanner: DUN	-0.0618	0.2836	-0.2178	0.8277		-0.6199	0.4964
	Scanner: GLA	-0.1637	0.2531	-0.6468	0.5182		-0.6617	0.3343
	Age	-0.0032	0.0520	-0.0617	0.9508		-0.1056	0.0992
	Sex: female	0.1866	0.1158	1.6118	0.1080		-0.0412	0.4145
	WML	-0.4349	0.0510	-8.5343	0.0000		-0.5351	-0.3346

	Depression score	-0.0184	0.0541	-0.3403	0.7339		-0.1249	0.0880
	DMT w1: yes	-0.0200	0.1243	-0.1605	0.8726		-0.2646	0.2247
NAWM cerebellar	Intercept	0.0501	0.2767	0.1811	0.8564		-0.4944	0.5946
	Fatigue group: fatigued	-0.1296	0.1240	-1.0451	0.2968	0.9603	-0.3737	0.1144
	Scanner: EDI1	-0.1973	0.2885	-0.6838	0.4946		-0.7651	0.3705
	Scanner: EDI2	0.1641	0.2886	0.5686	0.5701		-0.4038	0.7320
	Scanner: DUN	0.0261	0.3208	0.0814	0.9352		-0.6051	0.6574
	Scanner: GLA	-0.2851	0.2862	-0.9961	0.3200		-0.8484	0.2781
	Age	0.0499	0.0589	0.8483	0.3969		-0.0659	0.1657
	Sex: female	-0.0181	0.1309	-0.1382	0.8902		-0.2758	0.2396
	WML	-0.0562	0.0576	-0.9760	0.3298		-0.1697	0.0572
	Depression score	-0.0296	0.0612	-0.4832	0.6293		-0.1499	0.0908
	DMT w1: yes	0.1841	0.1406	1.3089	0.1915		-0.0927	0.4608
Pallidum	Intercept	-0.0923	0.2784	-0.3316	0.7404		-0.6402	0.4555
	Fatigue group: fatigued	-0.1033	0.1248	-0.8277	0.4085	0.9603	-0.3489	0.1423
	Scanner: EDI1	0.0014	0.2903	0.0047	0.9962		-0.5700	0.5727
	Scanner: EDI2	0.3191	0.2904	1.0990	0.2727		-0.2523	0.8906
	Scanner: DUN	0.0516	0.3228	0.1599	0.8731		-0.5836	0.6868
	Scanner: GLA	0.0031	0.2880	0.0107	0.9914		-0.5637	0.5699
	Age	0.0574	0.0592	0.9700	0.3328		-0.0591	0.1740
	Sex: female	-0.1018	0.1318	-0.7727	0.4403		-0.3611	0.1575
	WML	-0.0689	0.0580	-1.1890	0.2354		-0.1831	0.0452
	Depression score	-0.0181	0.0616	-0.2941	0.7689		-0.1392	0.1030
	DMT w1: yes	0.1825	0.1415	1.2897	0.1981		-0.0960	0.4610
Putamen	Intercept	-0.5440	0.2446	-2.2236	0.0269		-1.0254	-0.0626
	Fatigue group: fatigued	0.1287	0.1097	1.1740	0.2413	0.9603	-0.0871	0.3446
	Scanner: EDI1	0.3158	0.2551	1.2379	0.2167		-0.1862	0.8179
	Scanner: EDI2	0.0689	0.2552	0.2701	0.7873		-0.4332	0.5710
	Scanner: DUN	0.3310	0.2836	1.1669	0.2442		-0.2272	0.8891
	Scanner: GLA	0.1310	0.2531	0.5176	0.6051		-0.3670	0.6290
	Age	-0.2981	0.0520	-5.7279	0.0000		-0.4005	-0.1957
	Sex: female	0.4087	0.1158	3.5299	0.0005		0.1809	0.6365
	WML	-0.2997	0.0510	-5.8808	0.0000		-0.3999	-0.1994
	Depression score	-0.0070	0.0541	-0.1292	0.8973		-0.1134	0.0995
	DMT w1: yes	0.0066	0.1243	0.0529	0.9578		-0.2381	0.2513
Thalamus	Intercept	0.0260	0.2787	0.0933	0.9258		-0.5224	0.5744
	Fatigue group: fatigued	-0.1316	0.1249	-1.0535	0.2930	0.9603	-0.3774	0.1142
	Scanner: EDI1	0.0117	0.2906	0.0404	0.9678		-0.5602	0.5836
	Scanner: EDI2	0.2115	0.2907	0.7276	0.4674		-0.3605	0.7835
	Scanner: DUN	-0.0209	0.3231	-0.0646	0.9485		-0.6567	0.6149
	Scanner: GLA	-0.0677	0.2883	-0.2350	0.8144		-0.6350	0.4996
	Age	0.0429	0.0593	0.7236	0.4699		-0.0738	0.1595
	Sex: female	-0.1695	0.1319	-1.2849	0.1998		-0.4290	0.0901
	WML	-0.0737	0.0580	-1.2695	0.2053		-0.1879	0.0405
	Depression score	-0.0204	0.0616	-0.3308	0.7411		-0.1416	0.1009
	DMT w1: yes	0.1867	0.1416	1.3183	0.1884		-0.0920	0.4654

Ventral Diencephalon	Intercept	0.0193	0.2531	0.0764	0.9392		-0.4788	0.5175
	Fatigue group: fatigued	0.1800	0.1135	1.5860	0.1138	0.9603	-0.0433	0.4033
	Scanner: EDI1	-0.1604	0.2640	-0.6076	0.5439		-0.6799	0.3591
	Scanner: EDI2	-0.1847	0.2640	-0.6994	0.4848		-0.7042	0.3349
	Scanner: DUN	-0.3237	0.2935	-1.1028	0.2710		-0.9012	0.2539
	Scanner: GLA	-0.3757	0.2619	-1.4345	0.1525		-0.8910	0.1397
	Age	-0.1035	0.0538	-1.9220	0.0555		-0.2094	0.0025
	Sex: female	0.2553	0.1198	2.1311	0.0339		0.0196	0.4911
	WML	-0.3652	0.0527	-6.9271	0.0000		-0.4690	-0.2615
	Depression score	-0.0828	0.0560	-1.4794	0.1401		-0.1929	0.0273
	DMT w1: yes	-0.0463	0.1287	-0.3600	0.7191		-0.2995	0.2069
GM banks sts	Intercept	-0.7107	0.2524	-2.8151	0.0052		-1.2074	-0.2139
	Fatigue group: fatigued	-0.1670	0.1132	-1.4757	0.1411	0.9603	-0.3897	0.0557
	Scanner: EDI1	0.6547	0.2633	2.4867	0.0134		0.1366	1.1727
	Scanner: EDI2	0.6600	0.2633	2.5064	0.0127		0.1418	1.1781
	Scanner: DUN	0.5573	0.2927	1.9043	0.0578		-0.0186	1.1333
	Scanner: GLA	0.3462	0.2612	1.3256	0.1860		-0.1677	0.8601
	Age	-0.3432	0.0537	-6.3920	0.0000		-0.4489	-0.2376
	Sex: female	0.3636	0.1195	3.0430	0.0025		0.1285	0.5987
	WML	-0.0406	0.0526	-0.7722	0.4406		-0.1441	0.0629
	Depression score	0.0089	0.0558	0.1600	0.8730		-0.1009	0.1188
	DMT w1: yes	0.0242	0.1283	0.1885	0.8506		-0.2283	0.2767
GM caudal anterior cingulate	Intercept	-0.6821	0.2572	-2.6525	0.0084		-1.1882	-0.1761
	Fatigue group: fatigued	0.0964	0.1153	0.8359	0.4039	0.9603	-0.1305	0.3232
	Scanner: EDI1	0.6409	0.2682	2.3897	0.0175		0.1131	1.1687
	Scanner: EDI2	0.0413	0.2682	0.1540	0.8777		-0.4865	0.5691
	Scanner: DUN	0.9652	0.2982	3.2371	0.0013		0.3784	1.5519
	Scanner: GLA	0.3879	0.2660	1.4579	0.1459		-0.1357	0.9114
	Age	-0.1992	0.0547	-3.6419	0.0003		-0.3069	-0.0916
	Sex: female	0.3741	0.1217	3.0736	0.0023		0.1346	0.6136
	WML	-0.1481	0.0536	-2.7652	0.0060		-0.2535	-0.0427
	Depression score	-0.0794	0.0569	-1.3968	0.1635		-0.1913	0.0325
	DMT w1: yes	-0.0694	0.1307	-0.5309	0.5959		-0.3266	0.1878
GM caudal middle frontal	Intercept	-0.3271	0.2454	-1.3330	0.1835		-0.8099	0.1558
	Fatigue group: fatigued	0.0486	0.1100	0.4418	0.6589	0.9603	-0.1679	0.2650
	Scanner: EDI1	0.5802	0.2559	2.2676	0.0241		0.0767	1.0838
	Scanner: EDI2	-0.4674	0.2559	-1.8262	0.0688		-0.9710	0.0363
	Scanner: DUN	0.1717	0.2845	0.6036	0.5466		-0.3881	0.7315
	Scanner: GLA	0.2567	0.2538	1.0114	0.3126		-0.2428	0.7562
	Age	-0.2525	0.0522	-4.8378	0.0000		-0.3552	-0.1498
	Sex: female	0.4190	0.1161	3.6082	0.0004		0.1905	0.6475
	WML	-0.0653	0.0511	-1.2786	0.2020		-0.1659	0.0352
	Depression score	-0.1250	0.0543	-2.3034	0.0219		-0.2317	-0.0182
	DMT w1: yes	-0.2031	0.1247	-1.6287	0.1044		-0.4485	0.0423
GM cuneus	Intercept	-0.2669	0.2760	-0.9668	0.3344		-0.8100	0.2763
	Fatigue group: fatigued	0.0355	0.1237	0.2866	0.7746	0.9603	-0.2080	0.2789

	Scanner: EDI1	0.3283	0.2878	1.1404	0.2550		-0.2382	0.8947
	Scanner: EDI2	0.2384	0.2879	0.8281	0.4083		-0.3281	0.8049
	Scanner: DUN	0.0644	0.3200	0.2012	0.8407		-0.5653	0.6941
	Scanner: GLA	0.3892	0.2855	1.3629	0.1739		-0.1727	0.9511
	Age	-0.1369	0.0587	-2.3320	0.0204		-0.2524	-0.0214
	Sex: female	-0.0557	0.1306	-0.4264	0.6701		-0.3128	0.2014
	WML	-0.0790	0.0575	-1.3738	0.1705		-0.1921	0.0341
	Depression score	-0.0032	0.0610	-0.0517	0.9588		-0.1233	0.1169
	DMT w1: yes	0.0086	0.1403	0.0614	0.9511		-0.2675	0.2847
GM entorhinal	Intercept	-0.5693	0.2689	-2.1170	0.0351		-1.0985	-0.0401
	Fatigue group: fatigued	-0.1596	0.1206	-1.3237	0.1866	0.9603	-0.3968	0.0777
	Scanner: EDI1	0.9316	0.2805	3.3218	0.0010		0.3797	1.4836
	Scanner: EDI2	0.3854	0.2805	1.3738	0.1705		-0.1666	0.9374
	Scanner: DUN	0.7513	0.3118	2.4096	0.0166		0.1377	1.3649
	Scanner: GLA	0.8088	0.2782	2.9072	0.0039		0.2613	1.3563
	Age	-0.0824	0.0572	-1.4398	0.1510		-0.1949	0.0302
	Sex: female	-0.0858	0.1273	-0.6740	0.5008		-0.3362	0.1647
	WML	-0.1119	0.0560	-1.9972	0.0467		-0.2221	-0.0016
	Depression score	0.0767	0.0595	1.2903	0.1979		-0.0403	0.1937
	DMT w1: yes	0.0180	0.1367	0.1317	0.8953		-0.2510	0.2870
GM frontal pole	Intercept	-0.0534	0.2400	-0.2225	0.8241		-0.5258	0.4190
	Fatigue group: fatigued	-0.0634	0.1076	-0.5889	0.5564	0.9603	-0.2751	0.1484
	Scanner: EDI1	0.2092	0.2503	0.8357	0.4040		-0.2834	0.7018
	Scanner: EDI2	-0.0025	0.2504	-0.0100	0.9920		-0.4952	0.4902
	Scanner: DUN	-0.2134	0.2783	-0.7667	0.4438		-0.7610	0.3343
	Scanner: GLA	0.1928	0.2483	0.7765	0.4381		-0.2958	0.6815
	Age	-0.4884	0.0511	-9.5645	0.0000		-0.5888	-0.3879
	Sex: female	0.2438	0.1136	2.1461	0.0327		0.0202	0.4674
	WML	-0.0187	0.0500	-0.3749	0.7080		-0.1171	0.0796
	Depression score	0.0142	0.0531	0.2676	0.7892		-0.0902	0.1186
	DMT w1: yes	-0.2701	0.1220	-2.2143	0.0276		-0.5102	-0.0301
GM fusiform	Intercept	-0.7916	0.2518	-3.1441	0.0018		-1.2870	-0.2961
	Fatigue group: fatigued	-0.0042	0.1129	-0.0375	0.9701	0.9818	-0.2263	0.2179
	Scanner: EDI1	1.1631	0.2626	4.4299	0.0000		0.6464	1.6797
	Scanner: EDI2	0.5446	0.2626	2.0738	0.0389		0.0278	1.0613
	Scanner: DUN	0.7488	0.2919	2.5653	0.0108		0.1744	1.3232
	Scanner: GLA	0.9188	0.2604	3.5279	0.0005		0.4063	1.4313
	Age	-0.2575	0.0536	-4.8085	0.0000		-0.3629	-0.1521
	Sex: female	0.1317	0.1192	1.1055	0.2698		-0.1027	0.3662
	WML	-0.1680	0.0524	-3.2028	0.0015		-0.2711	-0.0648
	Depression score	-0.1103	0.0557	-1.9815	0.0484		-0.2198	-0.0008
	DMT w1: yes	-0.1767	0.1280	-1.3810	0.1683		-0.4285	0.0751
GM inferior parietal	Intercept	-0.8047	0.2534	-3.1760	0.0016		-1.3033	-0.3061
	Fatigue group: fatigued	-0.2027	0.1136	-1.7846	0.0753	0.9603	-0.4262	0.0208
	Scanner: EDI1	0.9444	0.2642	3.5740	0.0004		0.4244	1.4643
	Scanner: EDI2	0.4517	0.2643	1.7093	0.0884		-0.0683	0.9718

	Scanner: DUN	0.5611	0.2938	1.9102	0.0571		-0.0169	1.1392
	Scanner: GLA	0.3878	0.2621	1.4796	0.1400		-0.1280	0.9036
	Age	-0.2416	0.0539	-4.4834	0.0000		-0.3477	-0.1356
	Sex: female	0.4374	0.1199	3.6474	0.0003		0.2014	0.6733
	WML	-0.0969	0.0528	-1.8352	0.0675		-0.2007	0.0070
	Depression score	-0.0196	0.0560	-0.3503	0.7264		-0.1299	0.0906
	DMT w1: yes	0.0532	0.1288	0.4130	0.6799		-0.2002	0.3066
GM inferior temporal	Intercept	-0.5034	0.2556	-1.9697	0.0498		-1.0064	-0.0005
	Fatigue group: fatigued	-0.1301	0.1146	-1.1352	0.2572	0.9603	-0.3555	0.0954
	Scanner: EDI1	0.7216	0.2665	2.7074	0.0072		0.1971	1.2461
	Scanner: EDI2	0.0758	0.2666	0.2843	0.7764		-0.4488	0.6004
	Scanner: DUN	0.5415	0.2963	1.8273	0.0686		-0.0416	1.1246
	Scanner: GLA	0.5642	0.2644	2.1340	0.0336		0.0439	1.0845
	Age	-0.2866	0.0544	-5.2726	0.0000		-0.3936	-0.1797
	Sex: female	0.2116	0.1210	1.7491	0.0813		-0.0265	0.4496
	WML	-0.1020	0.0532	-1.9163	0.0563		-0.2068	0.0027
	Depression score	-0.0391	0.0565	-0.6913	0.4899		-0.1503	0.0721
	DMT w1: yes	-0.0720	0.1299	-0.5544	0.5797		-0.3276	0.1836
GM insula	Intercept	0.0748	0.2548	0.2937	0.7692		-0.4266	0.5763
	Fatigue group: fatigued	0.0299	0.1142	0.2614	0.7939	0.9603	-0.1949	0.2546
	Scanner: EDI1	0.2539	0.2657	0.9553	0.3402		-0.2691	0.7768
	Scanner: EDI2	-0.4527	0.2658	-1.7035	0.0895		-0.9757	0.0703
	Scanner: DUN	-0.5465	0.2954	-1.8501	0.0653		-1.1279	0.0348
	Scanner: GLA	-0.3518	0.2636	-1.3346	0.1830		-0.8705	0.1669
	Age	-0.2946	0.0542	-5.4363	0.0000		-0.4013	-0.1880
	Sex: female	0.1292	0.1206	1.0712	0.2849		-0.1081	0.3665
	WML	0.0279	0.0531	0.5263	0.5990		-0.0765	0.1324
	Depression score	-0.1065	0.0563	-1.8903	0.0597		-0.2174	0.0044
	DMT w1: yes	0.0774	0.1295	0.5975	0.5506		-0.1775	0.3322
GM isthmus cingulate	Intercept	-0.1746	0.2649	-0.6591	0.5103		-0.6958	0.3467
	Fatigue group: fatigued	0.1202	0.1187	1.0119	0.3124	0.9603	-0.1135	0.3538
	Scanner: EDI1	0.3986	0.2762	1.4432	0.1500		-0.1449	0.9422
	Scanner: EDI2	-0.2124	0.2763	-0.7687	0.4427		-0.7560	0.3313
	Scanner: DUN	0.0212	0.3071	0.0690	0.9450		-0.5831	0.6255
	Scanner: GLA	0.2121	0.2740	0.7741	0.4395		-0.3271	0.7513
	Age	-0.1934	0.0563	-3.4330	0.0007		-0.3043	-0.0826
	Sex: female	-0.0162	0.1254	-0.1294	0.8971		-0.2629	0.2305
	WML	-0.1543	0.0552	-2.7966	0.0055		-0.2629	-0.0457
	Depression score	0.0360	0.0586	0.6148	0.5391		-0.0792	0.1513
	DMT w1: yes	0.0105	0.1346	0.0781	0.9378		-0.2544	0.2754
GM lateral occipital	Intercept	-0.6321	0.2614	-2.4186	0.0162		-1.1465	-0.1178
	Fatigue group: fatigued	0.0287	0.1172	0.2451	0.8065	0.9603	-0.2018	0.2593
	Scanner: EDI1	1.0814	0.2726	3.9675	0.0001		0.5450	1.6178
	Scanner: EDI2	0.5660	0.2726	2.0763	0.0387		0.0296	1.1025
	Scanner: DUN	0.8951	0.3030	2.9538	0.0034		0.2988	1.4914
	Scanner: GLA	0.9790	0.2704	3.6209	0.0003		0.4470	1.5111

	Age	-0.2703	0.0556	-4.8611	0.0000		-0.3797	-0.1609
	Sex: female	-0.0792	0.1237	-0.6399	0.5227		-0.3226	0.1643
	WML	-0.0321	0.0544	-0.5891	0.5562		-0.1392	0.0751
	Depression score	0.0156	0.0578	0.2705	0.7870		-0.0981	0.1294
	DMT w1: yes	-0.2332	0.1328	-1.7553	0.0802		-0.4946	0.0282
GM lateral orbitofrontal	Intercept	-0.6286	0.2493	-2.5209	0.0122		-1.1193	-0.1379
	Fatigue group: fatigued	0.0168	0.1118	0.1507	0.8803	0.9603	-0.2031	0.2368
	Scanner: EDI1	0.6615	0.2600	2.5438	0.0115		0.1498	1.1732
	Scanner: EDI2	0.2244	0.2601	0.8627	0.3890		-0.2874	0.7362
	Scanner: DUN	0.2858	0.2891	0.9887	0.3236		-0.2831	0.8547
	Scanner: GLA	0.4334	0.2579	1.6800	0.0940		-0.0742	0.9410
	Age	-0.3748	0.0530	-7.0666	0.0000		-0.4792	-0.2704
	Sex: female	0.2344	0.1180	1.9862	0.0479		0.0022	0.4666
	WML	-0.0945	0.0519	-1.8202	0.0697		-0.1967	0.0077
	Depression score	-0.0653	0.0551	-1.1841	0.2373		-0.1738	0.0432
	DMT w1: yes	0.0618	0.1267	0.4874	0.6263		-0.1876	0.3112
GM lingual	Intercept	-0.2484	0.2691	-0.9231	0.3567		-0.7779	0.2811
	Fatigue group: fatigued	0.0544	0.1206	0.4509	0.6524	0.9603	-0.1830	0.2918
	Scanner: EDI1	0.2942	0.2806	1.0485	0.2952		-0.2580	0.8464
	Scanner: EDI2	0.2211	0.2807	0.7879	0.4314		-0.3312	0.7734
	Scanner: DUN	-0.0443	0.3120	-0.1420	0.8872		-0.6582	0.5696
	Scanner: GLA	0.1079	0.2784	0.3878	0.6984		-0.4398	0.6557
	Age	-0.1789	0.0572	-3.1256	0.0019		-0.2915	-0.0663
	Sex: female	0.0731	0.1273	0.5739	0.5664		-0.1775	0.3237
	WML	-0.1840	0.0560	-3.2829	0.0011		-0.2943	-0.0737
	Depression score	-0.0059	0.0595	-0.0989	0.9213		-0.1230	0.1112
	DMT w1: yes	0.0190	0.1368	0.1387	0.8898		-0.2502	0.2881
GM medial orbitofrontal	Intercept	-0.1272	0.2473	-0.5142	0.6075		-0.6139	0.3595
	Fatigue group: fatigued	0.1069	0.1109	0.9638	0.3359	0.9603	-0.1113	0.3251
	Scanner: EDI1	0.1309	0.2579	0.5073	0.6123		-0.3767	0.6384
	Scanner: EDI2	-0.0049	0.2580	-0.0189	0.9850		-0.5125	0.5028
	Scanner: DUN	-0.4730	0.2868	-1.6494	0.1001		-1.0373	0.0913
	Scanner: GLA	0.1281	0.2559	0.5007	0.6169		-0.3754	0.6316
	Age	-0.4043	0.0526	-7.6841	0.0000		-0.5078	-0.3007
	Sex: female	0.1304	0.1171	1.1136	0.2663		-0.1000	0.3607
	WML	-0.0841	0.0515	-1.6334	0.1034		-0.1855	0.0172
	Depression score	-0.0758	0.0547	-1.3865	0.1666		-0.1834	0.0318
	DMT w1: yes	-0.0619	0.1257	-0.4921	0.6230		-0.3092	0.1855
GM middle temporal	Intercept	-0.4720	0.2418	-1.9521	0.0519		-0.9478	0.0038
	Fatigue group: fatigued	-0.2735	0.1084	-2.5230	0.0121	0.9466	-0.4868	-0.0602
	Scanner: EDI1	0.6498	0.2522	2.5767	0.0104		0.1535	1.1460
	Scanner: EDI2	0.2610	0.2522	1.0350	0.3015		-0.2353	0.7573
	Scanner: DUN	0.1291	0.2803	0.4606	0.6454		-0.4226	0.6808
	Scanner: GLA	0.2380	0.2501	0.9515	0.3421		-0.2542	0.7303
	Age	-0.4129	0.0514	-8.0282	0.0000		-0.5141	-0.3117
	Sex: female	0.2499	0.1144	2.1837	0.0298		0.0247	0.4751

	WML	-0.0154	0.0504	-0.3062	0.7596		-0.1145	0.0837
	Depression score	0.0025	0.0535	0.0474	0.9622		-0.1027	0.1077
	DMT w1: yes	0.1292	0.1229	1.0510	0.2941		-0.1127	0.3710
GM paracentral	Intercept	-0.6492	0.2465	-2.6339	0.0089		-1.1343	-0.1642
	Fatigue group: fatigued	0.0553	0.1105	0.5006	0.6170	0.9603	-0.1621	0.2728
	Scanner: EDI1	0.6425	0.2571	2.4996	0.0130		0.1367	1.1484
	Scanner: EDI2	-0.1996	0.2571	-0.7764	0.4381		-0.7055	0.3063
	Scanner: DUN	0.4790	0.2858	1.6761	0.0947		-0.0834	1.0414
	Scanner: GLA	0.3181	0.2550	1.2476	0.2132		-0.1837	0.8199
	Age	-0.2623	0.0524	-5.0020	0.0000		-0.3654	-0.1591
	Sex: female	0.6136	0.1167	5.2601	0.0000		0.3841	0.8432
	WML	-0.0809	0.0513	-1.5767	0.1159		-0.1820	0.0201
	Depression score	-0.0841	0.0545	-1.5427	0.1240		-0.1913	0.0232
	DMT w1: yes	-0.1494	0.1253	-1.1923	0.2341		-0.3959	0.0972
GM parahippocampal	Intercept	-0.6909	0.2588	-2.6695	0.0080		-1.2003	-0.1816
	Fatigue group: fatigued	-0.0491	0.1160	-0.4233	0.6724	0.9603	-0.2774	0.1792
	Scanner: EDI1	0.3671	0.2699	1.3601	0.1748		-0.1641	0.8983
	Scanner: EDI2	0.2140	0.2700	0.7929	0.4285		-0.3172	0.7453
	Scanner: DUN	-0.0457	0.3001	-0.1522	0.8792		-0.6362	0.5448
	Scanner: GLA	0.3180	0.2678	1.1877	0.2359		-0.2089	0.8449
	Age	-0.1943	0.0551	-3.5285	0.0005		-0.3026	-0.0859
	Sex: female	0.5707	0.1225	4.6593	0.0000		0.3297	0.8118
	WML	-0.1389	0.0539	-2.5762	0.0105		-0.2450	-0.0328
	Depression score	-0.0154	0.0572	-0.2690	0.7881		-0.1280	0.0972
	DMT w1: yes	0.0470	0.1315	0.3574	0.7210		-0.2118	0.3059
GM pars opercularis	Intercept	-0.1247	0.2476	-0.5037	0.6148		-0.6119	0.3625
	Fatigue group: fatigued	-0.0617	0.1110	-0.5558	0.5787	0.9603	-0.2801	0.1567
	Scanner: EDI1	0.1479	0.2582	0.5727	0.5672		-0.3602	0.6559
	Scanner: EDI2	-0.3328	0.2582	-1.2889	0.1984		-0.8409	0.1753
	Scanner: DUN	-0.2196	0.2870	-0.7652	0.4448		-0.7844	0.3452
	Scanner: GLA	-0.1594	0.2561	-0.6223	0.5342		-0.6633	0.3446
	Age	-0.3786	0.0527	-7.1899	0.0000		-0.4822	-0.2750
	Sex: female	0.3835	0.1172	3.2729	0.0012		0.1529	0.6140
	WML	-0.1116	0.0516	-2.1637	0.0313		-0.2130	-0.0101
	Depression score	-0.0476	0.0547	-0.8696	0.3852		-0.1553	0.0601
	DMT w1: yes	-0.0084	0.1258	-0.0669	0.9467		-0.2560	0.2392
GM pars orbitalis	Intercept	-0.6393	0.2483	-2.5752	0.0105		-1.1279	-0.1508
	Fatigue group: fatigued	0.0269	0.1113	0.2416	0.8092	0.9603	-0.1921	0.2459
	Scanner: EDI1	0.7139	0.2589	2.7574	0.0062		0.2044	1.2234
	Scanner: EDI2	0.4937	0.2589	1.9066	0.0575		-0.0159	1.0033
	Scanner: DUN	0.4570	0.2878	1.5877	0.1134		-0.1094	1.0234
	Scanner: GLA	0.4287	0.2568	1.6690	0.0962		-0.0767	0.9341
	Age	-0.4321	0.0528	-8.1821	0.0000		-0.5360	-0.3282
	Sex: female	0.2095	0.1175	1.7827	0.0756		-0.0218	0.4407
	WML	0.0252	0.0517	0.4864	0.6270		-0.0766	0.1269
	Depression score	-0.0552	0.0549	-1.0049	0.3157		-0.1632	0.0529

	DMT w1: yes	-0.0289	0.1262	-0.2287	0.8192		-0.2772	0.2194
GM pars triangularis	Intercept	-0.2047	0.2569	-0.7968	0.4262		-0.7102	0.3008
	Fatigue group: fatigued	0.0676	0.1152	0.5870	0.5577	0.9603	-0.1590	0.2942
	Scanner: EDI1	0.4437	0.2679	1.6561	0.0987		-0.0835	0.9708
	Scanner: EDI2	-0.0427	0.2679	-0.1595	0.8734		-0.5700	0.4845
	Scanner: DUN	-0.0108	0.2978	-0.0364	0.9710		-0.5969	0.5753
	Scanner: GLA	0.1375	0.2657	0.5173	0.6053		-0.3855	0.6604
	Age	-0.3776	0.0546	-6.9099	0.0000		-0.4851	-0.2700
	Sex: female	0.1310	0.1216	1.0772	0.2823		-0.1083	0.3702
	WML	0.0465	0.0535	0.8687	0.3857		-0.0588	0.1518
	Depression score	-0.0199	0.0568	-0.3509	0.7259		-0.1317	0.0918
	DMT w1: yes	-0.0905	0.1306	-0.6930	0.4888		-0.3474	0.1664
GM pericalcarine	Intercept	-0.5193	0.2692	-1.9286	0.0547		-1.0491	0.0106
	Fatigue group: fatigued	-0.1098	0.1207	-0.9098	0.3637	0.9603	-0.3473	0.1277
	Scanner: EDI1	0.3685	0.2808	1.3126	0.1903		-0.1840	0.9211
	Scanner: EDI2	0.5817	0.2808	2.0716	0.0392		0.0291	1.1344
	Scanner: DUN	-0.0039	0.3121	-0.0123	0.9902		-0.6181	0.6104
	Scanner: GLA	0.2085	0.2785	0.7486	0.4547		-0.3396	0.7566
	Age	-0.1290	0.0573	-2.2531	0.0250		-0.2417	-0.0163
	Sex: female	0.2109	0.1274	1.6555	0.0989		-0.0398	0.4617
	WML	-0.0933	0.0561	-1.6643	0.0971		-0.2037	0.0170
	Depression score	0.0821	0.0595	1.3792	0.1689		-0.0350	0.1992
	DMT w1: yes	0.1523	0.1368	1.1128	0.2667		-0.1170	0.4215
GM postcentral	Intercept	-0.6404	0.2536	-2.5256	0.0121		-1.1394	-0.1414
	Fatigue group: fatigued	-0.0282	0.1137	-0.2484	0.8040	0.9603	-0.2519	0.1955
	Scanner: EDI1	0.9987	0.2644	3.7764	0.0002		0.4783	1.5190
	Scanner: EDI2	0.2163	0.2645	0.8179	0.4140		-0.3041	0.7368
	Scanner: DUN	0.5311	0.2940	1.8064	0.0719		-0.0475	1.1096
	Scanner: GLA	0.4183	0.2623	1.5945	0.1119		-0.0979	0.9345
	Age	-0.2415	0.0539	-4.4775	0.0000		-0.3477	-0.1354
	Sex: female	0.2959	0.1200	2.4657	0.0142		0.0597	0.5321
	WML	-0.1018	0.0528	-1.9277	0.0548		-0.2058	0.0021
	Depression score	-0.0923	0.0561	-1.6455	0.1009		-0.2026	0.0181
	DMT w1: yes	-0.0870	0.1289	-0.6754	0.5000		-0.3407	0.1666
GM posterior cingulate	Intercept	-0.1921	0.2491	-0.7714	0.4411		-0.6823	0.2980
	Fatigue group: fatigued	0.1291	0.1117	1.1565	0.2484	0.9603	-0.0906	0.3489
	Scanner: EDI1	0.5039	0.2598	1.9399	0.0533		-0.0073	1.0151
	Scanner: EDI2	-0.1915	0.2598	-0.7371	0.4616		-0.7027	0.3197
	Scanner: DUN	0.2695	0.2888	0.9332	0.3515		-0.2988	0.8378
	Scanner: GLA	0.0583	0.2577	0.2264	0.8210		-0.4487	0.5654
	Age	-0.3832	0.0530	-7.2323	0.0000		-0.4874	-0.2789
	Sex: female	0.1711	0.1179	1.4513	0.1477		-0.0609	0.4031
	WML	-0.0928	0.0519	-1.7879	0.0748		-0.1948	0.0093
	Depression score	-0.0057	0.0551	-0.1044	0.9169		-0.1141	0.1026
	DMT w1: yes	-0.1581	0.1266	-1.2489	0.2127		-0.4072	0.0910
GM pre[illegible]	Intercept	-0.6052	0.2401	-2.5206	0.0122		-1.0777	-0.1327

	Fatigue group: fatigued	0.0940	0.1076	0.8737	0.3830	0.9603	-0.1178	0.3058
	Scanner: EDI1	1.0524	0.2504	4.2029	0.0000		0.5596	1.5451
	Scanner: EDI2	0.0160	0.2504	0.0640	0.9490		-0.4768	0.5088
	Scanner: DUN	0.7058	0.2784	2.5353	0.0117		0.1580	1.2535
	Scanner: GLA	0.6559	0.2484	2.6408	0.0087		0.1671	1.1447
	Age	-0.2740	0.0511	-5.3642	0.0000		-0.3745	-0.1735
	Sex: female	0.3569	0.1136	3.1413	0.0018		0.1333	0.5806
	WML	-0.1040	0.0500	-2.0788	0.0385		-0.2024	-0.0055
	Depression score	-0.1453	0.0531	-2.7373	0.0066		-0.2498	-0.0408
	DMT w1: yes	-0.3757	0.1220	-3.0787	0.0023		-0.6158	-0.1356
GM precuneus	Intercept	-0.2200	0.2610	-0.8429	0.4000		-0.7336	0.2936
	Fatigue group: fatigued	-0.0661	0.1170	-0.5651	0.5724	0.9603	-0.2964	0.1641
	Scanner: EDI1	0.4105	0.2722	1.5081	0.1326		-0.1251	0.9461
	Scanner: EDI2	-0.0733	0.2722	-0.2693	0.7879		-0.6090	0.4624
	Scanner: DUN	-0.1522	0.3026	-0.5029	0.6154		-0.7476	0.4433
	Scanner: GLA	0.1224	0.2700	0.4535	0.6505		-0.4089	0.6538
	Age	-0.2564	0.0555	-4.6192	0.0000		-0.3657	-0.1472
	Sex: female	0.2803	0.1235	2.2692	0.0240		0.0372	0.5234
	WML	-0.1002	0.0544	-1.8426	0.0664		-0.2071	0.0068
	Depression score	-0.0410	0.0577	-0.7109	0.4777		-0.1546	0.0725
	DMT w1: yes	-0.0910	0.1327	-0.6863	0.4931		-0.3521	0.1700
GM rostral anterior cingulate	Intercept	-0.5524	0.2543	-2.1724	0.0306		-1.0528	-0.0520
	Fatigue group: fatigued	-0.0286	0.1140	-0.2507	0.8022	0.9603	-0.2529	0.1957
	Scanner: EDI1	0.7260	0.2652	2.7376	0.0066		0.2041	1.2479
	Scanner: EDI2	0.1993	0.2652	0.7514	0.4530		-0.3227	0.7212
	Scanner: DUN	0.8790	0.2948	2.9814	0.0031		0.2988	1.4592
	Scanner: GLA	0.5623	0.2631	2.1373	0.0334		0.0446	1.0799
	Age	-0.2889	0.0541	-5.3406	0.0000		-0.3953	-0.1824
	Sex: female	0.0489	0.1204	0.4061	0.6850		-0.1880	0.2857
	WML	-0.1360	0.0530	-2.5685	0.0107		-0.2403	-0.0318
	Depression score	-0.1459	0.0562	-2.5949	0.0099		-0.2565	-0.0353
	DMT w1: yes	0.0088	0.1292	0.0683	0.9456		-0.2455	0.2632
GM rostral middle frontal	Intercept	-0.3885	0.2469	-1.5735	0.1167		-0.8744	0.0974
	Fatigue group: fatigued	-0.0234	0.1107	-0.2117	0.8325	0.9603	-0.2412	0.1944
	Scanner: EDI1	0.6879	0.2575	2.6716	0.0080		0.1812	1.1946
	Scanner: EDI2	0.0326	0.2575	0.1266	0.8993		-0.4742	0.5394
	Scanner: DUN	0.3476	0.2863	1.2143	0.2256		-0.2157	0.9109
	Scanner: GLA	0.4929	0.2554	1.9297	0.0546		-0.0097	0.9955
	Age	-0.3781	0.0525	-7.1996	0.0000		-0.4815	-0.2748
	Sex: female	0.0011	0.1169	0.0093	0.9926		-0.2289	0.2310
	WML	-0.0889	0.0514	-1.7295	0.0847		-0.1901	0.0123
	Depression score	-0.0777	0.0546	-1.4229	0.1558		-0.1851	0.0297
	DMT w1: yes	0.0108	0.1255	0.0860	0.9315		-0.2362	0.2577
GM superior	Intercept	-0.1413	0.2275	-0.6210	0.5351		-0.5891	0.3065
	Fatigue group: fatigued	0.0299	0.1020	0.2929	0.7698	0.9603	-0.1708	0.2306
	Scanner: EDI1	0.5341	0.2373	2.2508	0.0251		0.0671	1.0011

	Scanner: EDI2	-0.6133	0.2373	-2.5841	0.0102		-1.0803	-0.1463
	Scanner: DUN	-0.1122	0.2638	-0.4251	0.6711		-0.6313	0.4070
	Scanner: GLA	0.0206	0.2354	0.0875	0.9303		-0.4426	0.4838
	Age	-0.4127	0.0484	-8.5278	0.0000		-0.5080	-0.3175
	Sex: female	0.2538	0.1077	2.3568	0.0191		0.0419	0.4657
	WML	-0.0764	0.0474	-1.6130	0.1078		-0.1697	0.0168
	Depression score	-0.0792	0.0503	-1.5749	0.1163		-0.1782	0.0198
	DMT w1: yes	-0.0499	0.1156	-0.4312	0.6666		-0.2774	0.1777
GM superior parietal	Intercept	-0.8047	0.2572	-3.1292	0.0019		-1.3107	-0.2986
	Fatigue group: fatigued	-0.0552	0.1153	-0.4791	0.6322	0.9603	-0.2821	0.1716
	Scanner: EDI1	0.7821	0.2682	2.9162	0.0038		0.2543	1.3098
	Scanner: EDI2	0.2437	0.2682	0.9085	0.3643		-0.2841	0.7715
	Scanner: DUN	0.3262	0.2981	1.0941	0.2748		-0.2605	0.9129
	Scanner: GLA	0.5789	0.2660	2.1761	0.0303		0.0554	1.1024
	Age	-0.2022	0.0547	-3.6970	0.0003		-0.3099	-0.0946
	Sex: female	0.5125	0.1217	4.2113	0.0000		0.2730	0.7520
	WML	-0.1117	0.0536	-2.0863	0.0378		-0.2172	-0.0063
	Depression score	-0.0453	0.0569	-0.7969	0.4262		-0.1572	0.0666
	DMT w1: yes	-0.0635	0.1307	-0.4860	0.6273		-0.3207	0.1937
GM superior temporal	Intercept	-0.4359	0.2430	-1.7937	0.0739		-0.9141	0.0423
	Fatigue group: fatigued	-0.0166	0.1089	-0.1528	0.8787	0.9603	-0.2310	0.1977
	Scanner: EDI1	0.4637	0.2534	1.8295	0.0683		-0.0351	0.9624
	Scanner: EDI2	0.3066	0.2535	1.2096	0.2274		-0.1922	0.8054
	Scanner: DUN	0.2738	0.2818	0.9718	0.3319		-0.2806	0.8282
	Scanner: GLA	0.2205	0.2514	0.8771	0.3811		-0.2742	0.7152
	Age	-0.4388	0.0517	-8.4886	0.0000		-0.5405	-0.3371
	Sex: female	0.3747	0.1150	3.2582	0.0012		0.1484	0.6011
	WML	-0.0854	0.0506	-1.6878	0.0925		-0.1850	0.0142
	Depression score	-0.0544	0.0537	-1.0117	0.3125		-0.1601	0.0514
	DMT w1: yes	-0.1754	0.1235	-1.4199	0.1567		-0.4184	0.0677
GM supramarginal	Intercept	-0.2000	0.2563	-0.7804	0.4358		-0.7045	0.3044
	Fatigue group: fatigued	-0.0228	0.1149	-0.1985	0.8428	0.9603	-0.2489	0.2033
	Scanner: EDI1	0.4111	0.2673	1.5379	0.1251		-0.1149	0.9371
	Scanner: EDI2	-0.0010	0.2674	-0.0037	0.9971		-0.5271	0.5251
	Scanner: DUN	-0.0431	0.2972	-0.1449	0.8849		-0.6279	0.5417
	Scanner: GLA	0.0257	0.2652	0.0970	0.9228		-0.4961	0.5475
	Age	-0.3528	0.0545	-6.4716	0.0000		-0.4601	-0.2456
	Sex: female	0.1280	0.1213	1.0552	0.2922		-0.1107	0.3667
	WML	-0.0822	0.0534	-1.5401	0.1246		-0.1873	0.0228
	Depression score	-0.0064	0.0567	-0.1137	0.9096		-0.1180	0.1051
	DMT w1: yes	0.0219	0.1303	0.1679	0.8668		-0.2345	0.2782
GM temporal pole	Intercept	-0.1200	0.2716	-0.4417	0.6591		-0.6544	0.4145
	Fatigue group: fatigued	-0.0126	0.1218	-0.1035	0.9176	0.9700	-0.2522	0.2270
	Scanner: EDI1	-0.0283	0.2833	-0.1000	0.9204		-0.5857	0.5291
	Scanner: EDI2	-0.1465	0.2833	-0.5171	0.6055		-0.7040	0.4110
	Scanner: DUN	-0.2793	0.3149	-0.8869	0.3759		-0.8989	0.3404

	Scanner: GLA	-0.0636	0.2810	-0.2263	0.8211		-0.6165	0.4893
	Age	-0.0445	0.0578	-0.7694	0.4422		-0.1581	0.0692
	Sex: female	0.4451	0.1285	3.4630	0.0006		0.1922	0.6981
	WML	-0.1257	0.0566	-2.2217	0.0270		-0.2370	-0.0144
	Depression score	-0.0640	0.0601	-1.0652	0.2876		-0.1821	0.0542
	DMT w1: yes	-0.1541	0.1380	-1.1163	0.2652		-0.4257	0.1176
GM transverse temporal	Intercept	-0.3150	0.2628	-1.1988	0.2315		-0.8321	0.2021
	Fatigue group: fatigued	0.2701	0.1178	2.2930	0.0225	0.9466	0.0383	0.5019
	Scanner: EDI1	0.1221	0.2740	0.4456	0.6562		-0.4172	0.6614
	Scanner: EDI2	0.0409	0.2741	0.1493	0.8814		-0.4984	0.5803
	Scanner: DUN	0.2502	0.3047	0.8212	0.4122		-0.3493	0.8497
	Scanner: GLA	-0.0991	0.2718	-0.3646	0.7157		-0.6341	0.4358
	Age	-0.2758	0.0559	-4.9346	0.0000		-0.3858	-0.1658
	Sex: female	0.4061	0.1244	3.2653	0.0012		0.1614	0.6508
	WML	-0.0404	0.0547	-0.7385	0.4608		-0.1481	0.0673
	Depression score	-0.0764	0.0581	-1.3158	0.1893		-0.1908	0.0379
	DMT w1: yes	-0.1889	0.1336	-1.4147	0.1582		-0.4518	0.0739
NAWM banks sts	Intercept	-0.6265	0.2618	-2.3929	0.0173		-1.1417	-0.1113
	Fatigue group: fatigued	-0.1531	0.1174	-1.3047	0.1930	0.9603	-0.3841	0.0778
	Scanner: EDI1	0.3410	0.2730	1.2490	0.2126		-0.1963	0.8783
	Scanner: EDI2	0.3839	0.2731	1.4057	0.1608		-0.1535	0.9212
	Scanner: DUN	0.4590	0.3035	1.5122	0.1315		-0.1383	1.0564
	Scanner: GLA	0.1268	0.2708	0.4681	0.6401		-0.4062	0.6598
	Age	-0.0368	0.0557	-0.6609	0.5092		-0.1464	0.0728
	Sex: female	0.4262	0.1239	3.4399	0.0007		0.1824	0.6701
	WML	-0.2696	0.0545	-4.9444	0.0000		-0.3769	-0.1623
	Depression score	-0.0327	0.0579	-0.5640	0.5732		-0.1466	0.0813
	DMT w1: yes	0.1505	0.1331	1.1311	0.2589		-0.1113	0.4124
NAWM caudal anterior cingulate	Intercept	-0.5133	0.2341	-2.1933	0.0290		-0.9739	-0.0528
	Fatigue group: fatigued	0.1015	0.1049	0.9670	0.3343	0.9603	-0.1050	0.3079
	Scanner: EDI1	0.6587	0.2441	2.6988	0.0074		0.1784	1.1391
	Scanner: EDI2	-0.1813	0.2441	-0.7426	0.4583		-0.6617	0.2991
	Scanner: DUN	0.7031	0.2714	2.5909	0.0100		0.1691	1.2371
	Scanner: GLA	0.0654	0.2421	0.2702	0.7872		-0.4110	0.5419
	Age	-0.0350	0.0498	-0.7039	0.4820		-0.1330	0.0629
	Sex: female	0.3353	0.1108	3.0271	0.0027		0.1173	0.5533
	WML	-0.4282	0.0488	-8.7845	0.0000		-0.5242	-0.3323
	Depression score	-0.1116	0.0518	-2.1569	0.0318		-0.2135	-0.0098
	DMT w1: yes	0.0095	0.1190	0.0796	0.9366		-0.2246	0.2436
NAWM caudal middle frontal	Intercept	-0.0521	0.2547	-0.2044	0.8382		-0.5534	0.4492
	Fatigue group: fatigued	0.0178	0.1142	0.1562	0.8760	0.9603	-0.2069	0.2426
	Scanner: EDI1	-0.0276	0.2657	-0.1037	0.9175		-0.5504	0.4952
	Scanner: EDI2	-0.7898	0.2657	-2.9725	0.0032		-1.3127	-0.2669
	Scanner: DUN	-0.2040	0.2954	-0.6907	0.4903		-0.7852	0.3772
	Scanner: GLA	-0.3278	0.2635	-1.2440	0.2145		-0.8464	0.1908
	Age	0.0000	0.0542	0.0000	1.0000		-0.1066	0.1066

	Sex: female	0.5057	0.1206	4.1946	0.0000		0.2685	0.7430
	WML	-0.2427	0.0531	-4.5737	0.0000		-0.3471	-0.1383
	Depression score	-0.1294	0.0563	-2.2982	0.0222		-0.2403	-0.0186
	DMT w1: yes	0.0035	0.1295	0.0272	0.9783		-0.2513	0.2583
NAWM cuneus	Intercept	0.4141	0.2768	1.4961	0.1357		-0.1306	0.9587
	Fatigue group: fatigued	0.0559	0.1241	0.4505	0.6527	0.9603	-0.1883	0.3000
	Scanner: EDI1	-0.0759	0.2886	-0.2629	0.7928		-0.6439	0.4921
	Scanner: EDI2	-0.4109	0.2887	-1.4236	0.1556		-0.9790	0.1571
	Scanner: DUN	-0.2047	0.3209	-0.6378	0.5241		-0.8361	0.4268
	Scanner: GLA	-0.1463	0.2863	-0.5109	0.6098		-0.7097	0.4172
	Age	0.0482	0.0589	0.8189	0.4135		-0.0676	0.1641
	Sex: female	-0.1535	0.1310	-1.1721	0.2421		-0.4113	0.1042
	WML	-0.0984	0.0576	-1.7074	0.0888		-0.2119	0.0150
	Depression score	0.0306	0.0612	0.4995	0.6178		-0.0899	0.1510
	DMT w1: yes	-0.1807	0.1407	-1.2847	0.1999		-0.4575	0.0961
NAWM entorhinal	Intercept	-0.4222	0.2721	-1.5517	0.1218		-0.9577	0.1132
	Fatigue group: fatigued	-0.1356	0.1220	-1.1116	0.2672	0.9603	-0.3756	0.1044
	Scanner: EDI1	0.6095	0.2838	2.1479	0.0325		0.0511	1.1679
	Scanner: EDI2	0.2668	0.2838	0.9399	0.3480		-0.2917	0.8253
	Scanner: DUN	0.6178	0.3155	1.9584	0.0511		-0.0030	1.2386
	Scanner: GLA	0.3262	0.2815	1.1589	0.2474		-0.2277	0.8801
	Age	0.0522	0.0579	0.9014	0.3681		-0.0617	0.1661
	Sex: female	-0.0078	0.1288	-0.0603	0.9520		-0.2612	0.2457
	WML	-0.2243	0.0567	-3.9582	0.0001		-0.3359	-0.1128
	Depression score	0.0010	0.0602	0.0164	0.9869		-0.1174	0.1194
	DMT w1: yes	0.1319	0.1383	0.9537	0.3410		-0.1403	0.4040
NAWM frontal pole	Intercept	0.1876	0.2740	0.6846	0.4941		-0.3516	0.7268
	Fatigue group: fatigued	-0.0110	0.1228	-0.0893	0.9289	0.9700	-0.2527	0.2307
	Scanner: EDI1	-0.1877	0.2857	-0.6569	0.5117		-0.7500	0.3746
	Scanner: EDI2	-0.5137	0.2858	-1.7975	0.0733		-1.0761	0.0487
	Scanner: DUN	-0.7051	0.3177	-2.2195	0.0272		-1.3302	-0.0800
	Scanner: GLA	-0.1387	0.2835	-0.4894	0.6249		-0.6965	0.4191
	Age	0.0122	0.0583	0.2096	0.8341		-0.1025	0.1269
	Sex: female	0.2003	0.1297	1.5450	0.1234		-0.0548	0.4555
	WML	-0.0788	0.0571	-1.3802	0.1686		-0.1911	0.0335
	Depression score	0.0076	0.0606	0.1247	0.9009		-0.1117	0.1268
	DMT w1: yes	-0.0556	0.1393	-0.3993	0.6899		-0.3297	0.2184
NAWM fusiform	Intercept	-0.5478	0.2535	-2.1606	0.0315		-1.0466	-0.0489
	Fatigue group: fatigued	0.0431	0.1136	0.3795	0.7046	0.9603	-0.1805	0.2668
	Scanner: EDI1	0.5089	0.2644	1.9247	0.0552		-0.0114	1.0292
	Scanner: EDI2	0.1828	0.2644	0.6912	0.4900		-0.3376	0.7031
	Scanner: DUN	0.2452	0.2939	0.8342	0.4048		-0.3332	0.8236
	Scanner: GLA	0.3304	0.2623	1.2596	0.2088		-0.1857	0.8465
	Age	-0.0260	0.0539	-0.4825	0.6298		-0.1321	0.0801
	Sex: female	0.2541	0.1200	2.1178	0.0350		0.0180	0.4902
	WML	-0.3951	0.0528	-7.4814	0.0000		-0.4990	-0.2911

	Depression score	-0.0667	0.0561	-1.1905	0.2348		-0.1770	0.0436
	DMT w1: yes	0.0355	0.1289	0.2753	0.7833		-0.2181	0.2890
NAWM inferior parietal	Intercept	-0.3117	0.2617	-1.1908	0.2347		-0.8267	0.2034
	Fatigue group: fatigued	-0.1973	0.1173	-1.6817	0.0937	0.9603	-0.4282	0.0336
	Scanner: EDI1	0.4178	0.2730	1.5305	0.1269		-0.1194	0.9549
	Scanner: EDI2	0.0424	0.2730	0.1553	0.8767		-0.4948	0.5796
	Scanner: DUN	0.1589	0.3035	0.5236	0.6009		-0.4383	0.7561
	Scanner: GLA	-0.1231	0.2708	-0.4545	0.6498		-0.6559	0.4098
	Age	0.0534	0.0557	0.9597	0.3380		-0.0561	0.1630
	Sex: female	0.1078	0.1239	0.8704	0.3848		-0.1359	0.3516
	WML	-0.3141	0.0545	-5.7621	0.0000		-0.4214	-0.2068
	Depression score	0.0196	0.0579	0.3391	0.7347		-0.0943	0.1335
	DMT w1: yes	0.3341	0.1330	2.5116	0.0125		0.0723	0.5959
NAWM inferior temporal	Intercept	-0.0377	0.2623	-0.1439	0.8857		-0.5539	0.4784
	Fatigue group: fatigued	-0.0993	0.1176	-0.8449	0.3988	0.9603	-0.3307	0.1320
	Scanner: EDI1	0.4611	0.2735	1.6857	0.0929		-0.0772	0.9994
	Scanner: EDI2	-0.2073	0.2736	-0.7578	0.4492		-0.7457	0.3310
	Scanner: DUN	-0.0381	0.3041	-0.1254	0.9003		-0.6366	0.5603
	Scanner: GLA	-0.0371	0.2713	-0.1368	0.8913		-0.5711	0.4968
	Age	-0.0222	0.0558	-0.3986	0.6905		-0.1320	0.0876
	Sex: female	-0.0384	0.1241	-0.3092	0.7574		-0.2827	0.2059
	WML	-0.2907	0.0546	-5.3219	0.0000		-0.3983	-0.1832
	Depression score	0.0200	0.0580	0.3449	0.7304		-0.0941	0.1341
	DMT w1: yes	0.1004	0.1333	0.7530	0.4521		-0.1620	0.3627
NAWM insula	Intercept	0.1330	0.2528	0.5262	0.5991		-0.3644	0.6304
	Fatigue group: fatigued	0.0690	0.1133	0.6091	0.5429	0.9603	-0.1540	0.2920
	Scanner: EDI1	0.2501	0.2636	0.9487	0.3435		-0.2687	0.7689
	Scanner: EDI2	-0.6190	0.2637	-2.3478	0.0195		-1.1379	-0.1002
	Scanner: DUN	-0.4933	0.2931	-1.6830	0.0934		-1.0700	0.0835
	Scanner: GLA	-0.4968	0.2615	-1.8999	0.0584		-1.0114	0.0178
	Age	0.0440	0.0538	0.8190	0.4134		-0.0618	0.1498
	Sex: female	0.2872	0.1196	2.4010	0.0170		0.0518	0.5227
	WML	-0.2510	0.0527	-4.7664	0.0000		-0.3546	-0.1473
	Depression score	-0.0584	0.0559	-1.0445	0.2971		-0.1684	0.0516
	DMT w1: yes	-0.0657	0.1285	-0.5115	0.6094		-0.3185	0.1871
NAWM isthmus cingulate	Intercept	0.0881	0.2479	0.3556	0.7224		-0.3996	0.5759
	Fatigue group: fatigued	0.1934	0.1111	1.7408	0.0827	0.9603	-0.0252	0.4121
	Scanner: EDI1	-0.2005	0.2585	-0.7757	0.4385		-0.7092	0.3081
	Scanner: EDI2	-0.4794	0.2585	-1.8544	0.0647		-0.9881	0.0293
	Scanner: DUN	-0.4790	0.2874	-1.6668	0.0966		-1.0444	0.0865
	Scanner: GLA	-0.4391	0.2564	-1.7127	0.0878		-0.9437	0.0654
	Age	0.1240	0.0527	2.3527	0.0193		0.0203	0.2278
	Sex: female	0.2467	0.1173	2.1033	0.0363		0.0159	0.4775
	WML	-0.4346	0.0516	-8.4182	0.0000		-0.5362	-0.3330
	Depression score	0.0115	0.0548	0.2102	0.8337		-0.0963	0.1194
	DMT w1: yes	0.0225	0.1260	0.1788	0.8582		-0.2254	0.2704

NAWM lateral occipital	Intercept	-0.0925	0.2717	-0.3406	0.7337		-0.6273	0.4422
	Fatigue group: fatigued	0.0697	0.1218	0.5722	0.5676	0.9603	-0.1700	0.3094
	Scanner: EDI1	0.5588	0.2834	1.9718	0.0495		0.0011	1.1165
	Scanner: EDI2	0.0566	0.2834	0.1998	0.8418		-0.5011	0.6144
	Scanner: DUN	0.5894	0.3151	1.8707	0.0624		-0.0306	1.2094
	Scanner: GLA	0.4487	0.2811	1.5961	0.1115		-0.1045	1.0019
	Age	-0.1151	0.0578	-1.9921	0.0473		-0.2289	-0.0014
	Sex: female	-0.2033	0.1286	-1.5811	0.1149		-0.4564	0.0497
	WML	-0.0886	0.0566	-1.5651	0.1186		-0.2000	0.0228
	Depression score	0.0415	0.0601	0.6901	0.4907		-0.0768	0.1597
	DMT w1: yes	-0.2210	0.1381	-1.6003	0.1106		-0.4928	0.0508
NAWM lateral orbitofrontal	Intercept	-0.4521	0.2599	-1.7391	0.0830		-0.9636	0.0595
	Fatigue group: fatigued	0.1582	0.1165	1.3579	0.1755	0.9603	-0.0711	0.3875
	Scanner: EDI1	0.4056	0.2711	1.4963	0.1356		-0.1278	0.9391
	Scanner: EDI2	-0.0461	0.2711	-0.1700	0.8651		-0.5796	0.4874
	Scanner: DUN	0.0418	0.3014	0.1388	0.8897		-0.5512	0.6349
	Scanner: GLA	-0.0794	0.2689	-0.2953	0.7680		-0.6086	0.4498
	Age	0.0566	0.0553	1.0240	0.3066		-0.0522	0.1654
	Sex: female	0.2474	0.1230	2.0113	0.0452		0.0053	0.4895
	WML	-0.3202	0.0541	-5.9141	0.0000		-0.4267	-0.2137
	Depression score	-0.0032	0.0575	-0.0557	0.9557		-0.1163	0.1099
	DMT w1: yes	0.1894	0.1321	1.4332	0.1528		-0.0706	0.4493
NAWM lingual	Intercept	-0.4507	0.2654	-1.6980	0.0905		-0.9730	0.0716
	Fatigue group: fatigued	0.0577	0.1190	0.4853	0.6278	0.9603	-0.1764	0.2919
	Scanner: EDI1	0.4151	0.2768	1.4994	0.1348		-0.1297	0.9598
	Scanner: EDI2	-0.0106	0.2769	-0.0383	0.9694		-0.5554	0.5342
	Scanner: DUN	0.0794	0.3077	0.2579	0.7966		-0.5262	0.6850
	Scanner: GLA	0.0927	0.2746	0.3376	0.7359		-0.4476	0.6331
	Age	0.0114	0.0565	0.2016	0.8404		-0.0997	0.1225
	Sex: female	0.2225	0.1256	1.7712	0.0775		-0.0247	0.4697
	WML	-0.2876	0.0553	-5.2028	0.0000		-0.3964	-0.1788
	Depression score	0.0092	0.0587	0.1563	0.8759		-0.1063	0.1247
	DMT w1: yes	0.1655	0.1349	1.2268	0.2209		-0.1000	0.4310
NAWM medial orbitofrontal	Intercept	0.4111	0.2671	1.5389	0.1249		-0.1146	0.9368
	Fatigue group: fatigued	0.1548	0.1198	1.2930	0.1970	0.9603	-0.0808	0.3905
	Scanner: EDI1	-0.0488	0.2786	-0.1751	0.8611		-0.5970	0.4994
	Scanner: EDI2	-0.3883	0.2786	-1.3937	0.1644		-0.9366	0.1600
	Scanner: DUN	-0.5399	0.3097	-1.7432	0.0823		-1.1494	0.0696
	Scanner: GLA	-0.2561	0.2763	-0.9269	0.3547		-0.8000	0.2877
	Age	0.0465	0.0568	0.8179	0.4141		-0.0653	0.1583
	Sex: female	-0.1514	0.1264	-1.1973	0.2321		-0.4002	0.0974
	WML	-0.2519	0.0556	-4.5273	0.0000		-0.3614	-0.1424
	Depression score	-0.0302	0.0591	-0.5110	0.6097		-0.1464	0.0860
	DMT w1: yes	-0.1486	0.1358	-1.0948	0.2745		-0.4158	0.1185
NAWM middle	Intercept	-0.0119	0.2695	-0.0440	0.9649		-0.5421	0.5184
	Fatigue group: fatigued	-0.1635	0.1208	-1.3534	0.1770	0.9603	-0.4012	0.0742

	Scanner: EDI1	0.0201	0.2810	0.0715	0.9431		-0.5329	0.5731
	Scanner: EDI2	-0.3258	0.2811	-1.1590	0.2474		-0.8789	0.2273
	Scanner: DUN	-0.2433	0.3124	-0.7788	0.4367		-0.8581	0.3715
	Scanner: GLA	-0.2606	0.2788	-0.9348	0.3507		-0.8092	0.2880
	Age	-0.0395	0.0573	-0.6884	0.4917		-0.1523	0.0733
	Sex: female	0.2192	0.1275	1.7190	0.0866		-0.0317	0.4702
	WML	-0.2328	0.0561	-4.1477	0.0000		-0.3433	-0.1224
	Depression score	0.0234	0.0596	0.3928	0.6947		-0.0938	0.1407
	DMT w1: yes	0.1622	0.1370	1.1843	0.2372		-0.1073	0.4317
NAWM paracentral	Intercept	0.0262	0.2602	0.1008	0.9198		-0.4858	0.5382
	Fatigue group: fatigued	0.0603	0.1166	0.5173	0.6053	0.9603	-0.1692	0.2899
	Scanner: EDI1	-0.2004	0.2713	-0.7384	0.4609		-0.7343	0.3336
	Scanner: EDI2	-0.6273	0.2714	-2.3114	0.0215		-1.1613	-0.0932
	Scanner: DUN	-0.1957	0.3017	-0.6487	0.5170		-0.7893	0.3979
	Scanner: GLA	-0.3222	0.2692	-1.1969	0.2323		-0.8519	0.2075
	Age	-0.0541	0.0553	-0.9775	0.3291		-0.1630	0.0548
	Sex: female	0.3426	0.1231	2.7820	0.0057		0.1003	0.5849
	WML	-0.3115	0.0542	-5.7471	0.0000		-0.4181	-0.2048
	Depression score	-0.0124	0.0575	-0.2148	0.8301		-0.1256	0.1009
	DMT w1: yes	0.0350	0.1322	0.2646	0.7915		-0.2252	0.2952
NAWM parahippocampal	Intercept	-0.2682	0.2611	-1.0272	0.3052		-0.7821	0.2456
	Fatigue group: fatigued	0.0281	0.1171	0.2396	0.8108	0.9603	-0.2023	0.2584
	Scanner: EDI1	0.1242	0.2723	0.4562	0.6486		-0.4117	0.6601
	Scanner: EDI2	-0.1159	0.2724	-0.4256	0.6707		-0.6519	0.4201
	Scanner: DUN	-0.2553	0.3028	-0.8434	0.3997		-0.8511	0.3404
	Scanner: GLA	-0.0190	0.2701	-0.0703	0.9440		-0.5506	0.5126
	Age	0.1499	0.0555	2.6986	0.0074		0.0406	0.2592
	Sex: female	0.2727	0.1236	2.2063	0.0281		0.0295	0.5159
	WML	-0.3329	0.0544	-6.1203	0.0000		-0.4399	-0.2259
	Depression score	-0.0298	0.0577	-0.5167	0.6057		-0.1435	0.0838
	DMT w1: yes	0.1152	0.1327	0.8680	0.3861		-0.1460	0.3764
NAWM pars opercularis	Intercept	0.0983	0.2678	0.3669	0.7139		-0.4288	0.6253
	Fatigue group: fatigued	0.0374	0.1201	0.3115	0.7556	0.9603	-0.1989	0.2737
	Scanner: EDI1	0.0324	0.2793	0.1158	0.9079		-0.5173	0.5820
	Scanner: EDI2	-0.5198	0.2794	-1.8607	0.0638		-1.0696	0.0299
	Scanner: DUN	-0.2573	0.3105	-0.8287	0.4079		-0.8684	0.3537
	Scanner: GLA	-0.2663	0.2771	-0.9611	0.3373		-0.8115	0.2790
	Age	-0.1221	0.0570	-2.1437	0.0329		-0.2342	-0.0100
	Sex: female	0.1683	0.1268	1.3277	0.1853		-0.0812	0.4177
	WML	-0.1978	0.0558	-3.5452	0.0005		-0.3076	-0.0880
	Depression score	-0.0377	0.0592	-0.6367	0.5248		-0.1542	0.0788
	DMT w1: yes	0.0032	0.1361	0.0234	0.9814		-0.2647	0.2711
NAWM pars orbitalis	Intercept	0.1771	0.2774	0.6385	0.5236		-0.3688	0.7230
	Fatigue group: fatigued	0.0121	0.1244	0.0974	0.9225	0.9700	-0.2326	0.2568
	Scanner: EDI1	0.2013	0.2893	0.6960	0.4870		-0.3679	0.7706
	Scanner: EDI2	-0.2409	0.2893	-0.8325	0.4058		-0.8102	0.3285

	Scanner: DUN	-0.2150	0.3216	-0.6684	0.5044		-0.8478	0.4179
	Scanner: GLA	-0.1140	0.2870	-0.3973	0.6914		-0.6787	0.4507
	Age	0.0046	0.0590	0.0776	0.9382		-0.1115	0.1207
	Sex: female	-0.1961	0.1313	-1.4938	0.1363		-0.4545	0.0622
	WML	-0.0661	0.0578	-1.1437	0.2536		-0.1798	0.0476
	Depression score	0.0307	0.0613	0.5005	0.6171		-0.0900	0.1514
	DMT w1: yes	0.0574	0.1410	0.4073	0.6841		-0.2200	0.3349
NAWM pars triangularis	Intercept	-0.0793	0.2753	-0.2879	0.7736		-0.6209	0.4624
	Fatigue group: fatigued	0.1223	0.1234	0.9913	0.3223	0.9603	-0.1205	0.3651
	Scanner: EDI1	0.3532	0.2871	1.2303	0.2195		-0.2117	0.9181
	Scanner: EDI2	-0.1314	0.2871	-0.4576	0.6476		-0.6963	0.4336
	Scanner: DUN	0.0878	0.3191	0.2752	0.7834		-0.5402	0.7158
	Scanner: GLA	0.0488	0.2848	0.1713	0.8641		-0.5116	0.6091
	Age	-0.0129	0.0585	-0.2201	0.8259		-0.1281	0.1023
	Sex: female	-0.0830	0.1303	-0.6372	0.5245		-0.3394	0.1733
	WML	-0.1452	0.0573	-2.5323	0.0118		-0.2580	-0.0324
	Depression score	-0.0456	0.0609	-0.7490	0.4544		-0.1654	0.0742
	DMT w1: yes	0.0114	0.1399	0.0814	0.9352		-0.2639	0.2867
NAWM pericalcarine	Intercept	-0.2213	0.2632	-0.8406	0.4012		-0.7393	0.2967
	Fatigue group: fatigued	0.0096	0.1180	0.0811	0.9354	0.9700	-0.2227	0.2418
	Scanner: EDI1	0.2605	0.2745	0.9490	0.3434		-0.2797	0.8008
	Scanner: EDI2	-0.1737	0.2746	-0.6325	0.5275		-0.7140	0.3666
	Scanner: DUN	-0.1336	0.3052	-0.4379	0.6618		-0.7343	0.4670
	Scanner: GLA	0.0433	0.2723	0.1591	0.8737		-0.4926	0.5792
	Age	-0.0240	0.0560	-0.4291	0.6681		-0.1342	0.0862
	Sex: female	0.1036	0.1246	0.8318	0.4062		-0.1415	0.3488
	WML	-0.3078	0.0548	-5.6142	0.0000		-0.4157	-0.1999
	Depression score	0.0801	0.0582	1.3764	0.1697		-0.0344	0.1947
	DMT w1: yes	0.1612	0.1338	1.2049	0.2292		-0.1021	0.4245
NAWM postcentral	Intercept	0.3796	0.2669	1.4221	0.1560		-0.1457	0.9048
	Fatigue group: fatigued	0.0429	0.1197	0.3586	0.7201	0.9603	-0.1925	0.2784
	Scanner: EDI1	-0.0513	0.2784	-0.1843	0.8539		-0.5991	0.4965
	Scanner: EDI2	-0.7768	0.2784	-2.7903	0.0056		-1.3246	-0.2290
	Scanner: DUN	-0.5762	0.3095	-1.8620	0.0636		-1.1852	0.0328
	Scanner: GLA	-0.5518	0.2761	-1.9985	0.0466		-1.0952	-0.0085
	Age	0.0456	0.0568	0.8030	0.4226		-0.0661	0.1573
	Sex: female	0.1342	0.1263	1.0626	0.2888		-0.1144	0.3828
	WML	-0.1542	0.0556	-2.7732	0.0059		-0.2636	-0.0448
	Depression score	-0.0573	0.0590	-0.9717	0.3320		-0.1735	0.0588
	DMT w1: yes	-0.0447	0.1357	-0.3294	0.7420		-0.3116	0.2223
NAWM posterior cingulate	Intercept	-0.1248	0.2215	-0.5634	0.5736		-0.5605	0.3110
	Fatigue group: fatigued	0.1246	0.0993	1.2555	0.2103	0.9603	-0.0707	0.3200
	Scanner: EDI1	0.3763	0.2309	1.6295	0.1042		-0.0781	0.8308
	Scanner: EDI2	-0.3950	0.2310	-1.7102	0.0883		-0.8495	0.0595
	Scanner: DUN	0.0546	0.2567	0.2125	0.8319		-0.4507	0.5598
	Scanner: GLA	-0.0618	0.2291	-0.2698	0.7875		-0.5126	0.3890

	Age	-0.0425	0.0471	-0.9018	0.3679		-0.1352	0.0502
	Sex: female	0.2536	0.1048	2.4200	0.0161		0.0474	0.4599
	WML	-0.5479	0.0461	-11.8782	0.0000		-0.6387	-0.4571
	Depression score	-0.0043	0.0490	-0.0881	0.9299		-0.1007	0.0920
	DMT w1: yes	-0.1329	0.1126	-1.1810	0.2385		-0.3544	0.0886
NAWM precentral	Intercept	0.0903	0.2606	0.3463	0.7293		-0.4226	0.6031
	Fatigue group: fatigued	0.1322	0.1168	1.1314	0.2588	0.9603	-0.0977	0.3621
	Scanner: EDI1	0.1450	0.2718	0.5335	0.5941		-0.3898	0.6799
	Scanner: EDI2	-0.4961	0.2718	-1.8251	0.0690		-1.0311	0.0388
	Scanner: DUN	0.0782	0.3022	0.2590	0.7958		-0.5164	0.6729
	Scanner: GLA	-0.2419	0.2696	-0.8973	0.3703		-0.7725	0.2886
	Age	0.0664	0.0554	1.1976	0.2320		-0.0427	0.1755
	Sex: female	0.2577	0.1233	2.0892	0.0375		0.0150	0.5004
	WML	-0.2463	0.0543	-4.5376	0.0000		-0.3532	-0.1395
	Depression score	-0.0870	0.0576	-1.5102	0.1320		-0.2004	0.0264
	DMT w1: yes	-0.2404	0.1325	-1.8148	0.0705		-0.5011	0.0203
NAWM precuneus	Intercept	0.3523	0.2567	1.3724	0.1710		-0.1529	0.8574
	Fatigue group: fatigued	-0.0227	0.1151	-0.1969	0.8441	0.9603	-0.2491	0.2038
	Scanner: EDI1	-0.1470	0.2677	-0.5490	0.5834		-0.6738	0.3798
	Scanner: EDI2	-0.3394	0.2677	-1.2675	0.2059		-0.8662	0.1875
	Scanner: DUN	-0.3920	0.2976	-1.3172	0.1888		-0.9777	0.1936
	Scanner: GLA	-0.3619	0.2656	-1.3627	0.1740		-0.8844	0.1607
	Age	0.0604	0.0546	1.1066	0.2693		-0.0470	0.1679
	Sex: female	-0.0528	0.1215	-0.4342	0.6644		-0.2918	0.1863
	WML	-0.4067	0.0535	-7.6065	0.0000		-0.5119	-0.3015
	Depression score	0.0075	0.0568	0.1316	0.8954		-0.1042	0.1192
	DMT w1: yes	-0.0117	0.1305	-0.0896	0.9287		-0.2684	0.2450
NAWM rostral anterior cingulate	Intercept	0.1797	0.2686	0.6689	0.5040		-0.3489	0.7083
	Fatigue group: fatigued	0.0586	0.1204	0.4867	0.6268	0.9603	-0.1783	0.2956
	Scanner: EDI1	-0.2209	0.2801	-0.7887	0.4309		-0.7722	0.3303
	Scanner: EDI2	-0.7003	0.2802	-2.4996	0.0130		-1.2516	-0.1490
	Scanner: DUN	-0.4950	0.3114	-1.5895	0.1130		-1.1078	0.1178
	Scanner: GLA	-0.4284	0.2779	-1.5415	0.1242		-0.9752	0.1185
	Age	-0.0585	0.0571	-1.0237	0.3068		-0.1709	0.0539
	Sex: female	0.1700	0.1271	1.3369	0.1822		-0.0802	0.4201
	WML	-0.2201	0.0559	-3.9336	0.0001		-0.3302	-0.1100
	Depression score	0.0201	0.0594	0.3390	0.7349		-0.0967	0.1370
	DMT w1: yes	0.1328	0.1365	0.9725	0.3316		-0.1359	0.4014
NAWM rostral middle frontal	Intercept	-0.1146	0.2656	-0.4317	0.6663		-0.6373	0.4080
	Fatigue group: fatigued	0.0025	0.1191	0.0211	0.9832	0.9832	-0.2318	0.2368
	Scanner: EDI1	0.4470	0.2770	1.6138	0.1076		-0.0981	0.9920
	Scanner: EDI2	-0.2498	0.2770	-0.9018	0.3679		-0.7949	0.2953
	Scanner: DUN	0.2571	0.3079	0.8350	0.4044		-0.3488	0.8630
	Scanner: GLA	0.1834	0.2747	0.6676	0.5049		-0.3572	0.7241
	Age	-0.0512	0.0565	-0.9069	0.3652		-0.1624	0.0599
	Sex: female	-0.1538	0.1257	-1.2240	0.2219		-0.4012	0.0935

	WML	-0.2233	0.0553	-4.0360	0.0001		-0.3321	-0.1144
	Depression score	-0.0420	0.0587	-0.7160	0.4745		-0.1576	0.0735
	DMT w1: yes	0.1193	0.1350	0.8836	0.3776		-0.1464	0.3849
NAWM superior frontal	Intercept	0.2639	0.2617	1.0081	0.3142		-0.2512	0.7789
	Fatigue group: fatigued	0.0945	0.1173	0.8050	0.4215	0.9603	-0.1364	0.3254
	Scanner: EDI1	-0.0941	0.2730	-0.3447	0.7306		-0.6312	0.4431
	Scanner: EDI2	-0.8991	0.2730	-3.2933	0.0011		-1.4363	-0.3618
	Scanner: DUN	-0.3524	0.3035	-1.1612	0.2465		-0.9496	0.2448
	Scanner: GLA	-0.4521	0.2708	-1.6696	0.0960		-0.9849	0.0808
	Age	0.0022	0.0557	0.0388	0.9691		-0.1074	0.1117
	Sex: female	0.0920	0.1239	0.7424	0.4584		-0.1518	0.3357
	WML	-0.2441	0.0545	-4.4766	0.0000		-0.3513	-0.1368
	Depression score	-0.0540	0.0579	-0.9337	0.3512		-0.1679	0.0599
	DMT w1: yes	0.0910	0.1330	0.6842	0.4944		-0.1708	0.3528
NAWM superior parietal	Intercept	-0.1879	0.2642	-0.7111	0.4776		-0.7078	0.3320
	Fatigue group: fatigued	0.0535	0.1184	0.4515	0.6520	0.9603	-0.1796	0.2865
	Scanner: EDI1	0.0781	0.2755	0.2833	0.7771		-0.4641	0.6203
	Scanner: EDI2	-0.2730	0.2756	-0.9907	0.3226		-0.8153	0.2693
	Scanner: DUN	-0.3305	0.3063	-1.0788	0.2815		-0.9333	0.2723
	Scanner: GLA	-0.0454	0.2733	-0.1662	0.8681		-0.5833	0.4924
	Age	0.0207	0.0562	0.3679	0.7132		-0.0899	0.1313
	Sex: female	0.2918	0.1250	2.3340	0.0203		0.0458	0.5379
	WML	-0.2902	0.0550	-5.2739	0.0000		-0.3985	-0.1819
	Depression score	-0.0551	0.0584	-0.9431	0.3464		-0.1701	0.0599
	DMT w1: yes	0.0596	0.1343	0.4437	0.6576		-0.2047	0.3238
NAWM superior temporal	Intercept	0.2877	0.2686	1.0712	0.2849		-0.2408	0.8163
	Fatigue group: fatigued	0.1326	0.1204	1.1017	0.2715	0.9603	-0.1043	0.3696
	Scanner: EDI1	-0.0406	0.2801	-0.1449	0.8849		-0.5918	0.5106
	Scanner: EDI2	-0.4254	0.2801	-1.5185	0.1299		-0.9767	0.1259
	Scanner: DUN	-0.2019	0.3114	-0.6484	0.5172		-0.8147	0.4109
	Scanner: GLA	-0.3561	0.2779	-1.2817	0.2009		-0.9029	0.1907
	Age	0.0452	0.0571	0.7904	0.4299		-0.0673	0.1576
	Sex: female	-0.1366	0.1271	-1.0744	0.2835		-0.3867	0.1136
	WML	-0.2642	0.0559	-4.7222	0.0000		-0.3743	-0.1541
	Depression score	-0.0541	0.0594	-0.9105	0.3633		-0.1709	0.0628
	DMT w1: yes	0.0293	0.1365	0.2148	0.8301		-0.2393	0.2980
NAWM supramarginal	Intercept	0.3378	0.2684	1.2583	0.2093		-0.1905	0.8660
	Fatigue group: fatigued	0.0857	0.1203	0.7125	0.4767	0.9603	-0.1511	0.3225
	Scanner: EDI1	-0.1300	0.2799	-0.4644	0.6427		-0.6809	0.4209
	Scanner: EDI2	-0.4442	0.2800	-1.5866	0.1136		-0.9952	0.1067
	Scanner: DUN	-0.4900	0.3112	-1.5744	0.1164		-1.1024	0.1224
	Scanner: GLA	-0.4845	0.2777	-1.7447	0.0820		-1.0309	0.0620
	Age	0.0344	0.0571	0.6031	0.5469		-0.0779	0.1468
	Sex: female	-0.1368	0.1270	-1.0770	0.2824		-0.3868	0.1132
	WML	-0.2678	0.0559	-4.7895	0.0000		-0.3778	-0.1578
	Depression score	0.0033	0.0594	0.0559	0.9555		-0.1135	0.1201

	DMT w1: yes	0.1335	0.1364	0.9788	0.3285		-0.1349	0.4020
NAWM temporal pole	Intercept	0.3093	0.2674	1.1569	0.2482		-0.2168	0.8355
	Fatigue group: fatigued	0.0281	0.1199	0.2341	0.8151	0.9603	-0.2078	0.2639
	Scanner: EDI1	0.2501	0.2788	0.8971	0.3704		-0.2986	0.7988
	Scanner: EDI2	-0.1816	0.2789	-0.6511	0.5155		-0.7304	0.3672
	Scanner: DUN	-0.4080	0.3100	-1.3162	0.1891		-1.0180	0.2020
	Scanner: GLA	0.0253	0.2766	0.0914	0.9272		-0.5190	0.5696
	Age	0.1091	0.0569	1.9180	0.0561		-0.0028	0.2210
	Sex: female	-0.1859	0.1265	-1.4687	0.1429		-0.4349	0.0632
	WML	-0.1955	0.0557	-3.5109	0.0005		-0.3051	-0.0859
	Depression score	0.0035	0.0591	0.0600	0.9522		-0.1128	0.1199
	DMT w1: yes	-0.2208	0.1359	-1.6251	0.1052		-0.4883	0.0466
NAWM transverse temporal	Intercept	-0.3769	0.2751	-1.3700	0.1717		-0.9182	0.1645
	Fatigue group: fatigued	0.1003	0.1233	0.8135	0.4166	0.9603	-0.1423	0.3430
	Scanner: EDI1	0.1218	0.2869	0.4246	0.6714		-0.4427	0.6863
	Scanner: EDI2	0.0698	0.2869	0.2433	0.8080		-0.4948	0.6344
	Scanner: DUN	0.0768	0.3189	0.2408	0.8098		-0.5508	0.7044
	Scanner: GLA	0.1046	0.2846	0.3674	0.7136		-0.4554	0.6646
	Age	0.0921	0.0585	1.5743	0.1165		-0.0230	0.2073
	Sex: female	0.3532	0.1302	2.7126	0.0071		0.0970	0.6094
	WML	-0.1290	0.0573	-2.2518	0.0251		-0.2418	-0.0163
	Depression score	0.0330	0.0608	0.5422	0.5881		-0.0867	0.1527
	DMT w1: yes	-0.0356	0.1398	-0.2543	0.7994		-0.3107	0.2396
WML	Intercept	-0.1691	0.2756	-0.6135	0.5400		-0.7115	0.3733
	Fatigue group: fatigued	-0.0865	0.1235	-0.7002	0.4844	0.9603	-0.3296	0.1566
	Scanner: EDI1	0.2579	0.2873	0.8976	0.3701		-0.3074	0.8231
	Scanner: EDI2	0.1054	0.2876	0.3665	0.7143		-0.4606	0.6714
	Scanner: DUN	0.4158	0.3189	1.3039	0.1932		-0.2117	1.0433
	Scanner: GLA	0.1240	0.2852	0.4349	0.6639		-0.4373	0.6853
	Age	0.1720	0.0578	2.9738	0.0032		0.0582	0.2858
	Sex: female	-0.1065	0.1304	-0.8171	0.4145		-0.3631	0.1500
	Depression score	0.0307	0.0610	0.5037	0.6148		-0.0893	0.1507
	DMT w1: yes	0.1531	0.1399	1.0946	0.2745		-0.1222	0.4285
Whole-brain	Intercept	-0.4496	0.2364	-1.9016	0.0582		-0.9149	0.0156
	Fatigue group: fatigued	-0.0188	0.1060	-0.1774	0.8593	0.9603	-0.2273	0.1897
	Scanner: EDI1	0.7366	0.2464	2.9893	0.0030		0.2517	1.2215
	Scanner: EDI2	-0.2897	0.2467	-1.1743	0.2412		-0.7752	0.1958
	Scanner: DUN	0.1157	0.2735	0.4229	0.6727		-0.4226	0.6539
	Scanner: GLA	0.1125	0.2447	0.4596	0.6461		-0.3690	0.5939
	Age	-0.3620	0.0496	-7.2966	0.0000		-0.4596	-0.2643
	Sex: female	0.4018	0.1118	3.5928	0.0004		0.1817	0.6219
	Depression score	-0.0863	0.0523	-1.6498	0.1000		-0.1892	0.0166
	DMT w1: yes	0.0017	0.1200	0.0144	0.9886		-0.2344	0.2379
SCCSA-C2-3	Intercept	0.5017	0.2695	1.8614	0.0636		-0.0286	1.0320
	Fatigue group: fatigued	-0.0382	0.1191	-0.3206	0.7487	0.7487	-0.2725	0.1962
	Scanner: EDI1	-0.7352	0.2744	-2.6797	0.0078		-1.2750	-0.1954

	Scanner: EDI2	-0.8608	0.2744	-3.1374	0.0019		-1.4007	-0.3210
	Scanner: DUN	-0.4810	0.3033	-1.5859	0.1138		-1.0777	0.1157
	Scanner: GLA	-0.6190	0.2706	-2.2877	0.0228		-1.1514	-0.0866
	Age	-0.0635	0.0562	-1.1295	0.2596		-0.1742	0.0471
	Sex: female	-0.0660	0.1271	-0.5192	0.6040		-0.3161	0.1841
	Depression score	-0.0728	0.0591	-1.2315	0.2191		-0.1891	0.0435
	DMT w1: yes	0.2905	0.1387	2.0946	0.0370		0.0176	0.5633

RRMS=relapsing-remitting multiple sclerosis, FDR=false discovery rate, $B_{standardised}$=standardised beta value, SE=standard error, CI=confidence interval for beta value, w0=baseline, w1=1-year follow-up, GM=grey matter, NAWM=normal-appearing white matter, sts=superior temporal sulcus, WML=white matter lesion, ED1=Edinburgh scanner 1, EDI2=Edinburgh scanner 2, DUN=Dundee, GLA=Glasgow, SCCSA-C2-3=spinal cord cross-sectional area cervical levels 2 and 3, PHQ-9=patient health questionnaire, FSS=fatigue severity scale, DMT=disease-modifying treatment

Supplementary Table 5. Results for longitudinal differences of brain tissue volumes, WML volumes and SCCSA-C2-3 between RRMS participants with and without fatigue (based on FSS score). This was assessed using multiple linear regression models, with the interaction of time point and fatigue group as regressor of interest, and MRI scanner, age_{w0}, sex, DMT intake at w1, WML_{w0} (not for WML, whole-brain and SCCSA) and depression score (based on PHQ-9) as control variables. P-values for the regressor of interest were corrected for multiple comparisons using the FDR (q<.05).

		$B_{standardised}$	SD	t-value	$p_{uncorrected}$	p_{FDR}	CI 2.5%	CI 97.5%
**Brainstem**	Intercept	-0.0927	0.2594	-0.3575	0.7210		-0.5977	0.4123
	Time: w1	-0.0175	0.0168	-1.0389	0.2996		-0.0503	0.0153
	Fatigue group: fatigued	0.0225	0.1114	0.2020	0.8401		-0.1944	0.2394
	Age	-0.1316	0.0548	-2.3990	0.0170		-0.2384	-0.0248
	Sex: female	0.2252	0.1235	1.8242	0.0691		-0.0152	0.4657
	Scanner: EDI1	0.3784	0.2723	1.3897	0.1657		-0.1518	0.9086
	Scanner: EDI2	-0.3949	0.2726	-1.4487	0.1485		-0.9258	0.1359
	Scanner: DUN	-0.1400	0.3017	-0.4640	0.6430		-0.7274	0.4475
	Scanner: GLA	-0.2692	0.2701	-0.9969	0.3196		-0.7952	0.2567
	DMT w1: yes	0.0877	0.1326	0.6611	0.5090		-0.1706	0.3459
	WML	-0.0315	0.0236	-1.3371	0.1822		-0.0774	0.0144
	Depression score	-0.0229	0.0152	-1.5095	0.1322		-0.0524	0.0066
	Time*Fatigue group	-0.0775	0.0231	-3.3557	0.0009	0.0748	-0.1224	-0.0325
**Accumbens**	Intercept	-0.3707	0.2482	-1.4931	0.1364		-0.8540	0.1127
	Time: w1	-0.0518	0.0263	-1.9691	0.0498		-0.1031	-0.0006
	Fatigue group: fatigued	0.1989	0.1075	1.8497	0.0653		-0.0105	0.4083
	Age	-0.2555	0.0524	-4.8721	0.0000		-0.3576	-0.1534
	Sex: female	0.4233	0.1181	3.5842	0.0004		0.1933	0.6533
	Scanner: EDI1	-0.0416	0.2604	-0.1596	0.8733		-0.5486	0.4654
	Scanner: EDI2	0.1707	0.2606	0.6551	0.5129		-0.3367	0.6781
	Scanner: DUN	0.0459	0.2888	0.1588	0.8739		-0.5165	0.6082
	Scanner: GLA	0.1234	0.2583	0.4777	0.6332		-0.3795	0.6263
	DMT w1: yes	-0.1291	0.1268	-1.0183	0.3094		-0.3759	0.1178
	WML	-0.1010	0.0326	-3.1008	0.0021		-0.1645	-0.0376
	Depression score	-0.0124	0.0230	-0.5392	0.5902		-0.0572	0.0324
	Time*Fatigue group	0.0031	0.0368	0.0833	0.9336	0.9997	-0.0686	0.0747
**Amygdala**	Intercept	0.2448	0.2576	0.9506	0.3426		-0.2567	0.7463
	Time: w1	-0.1270	0.0250	-5.0803	0.0000		-0.1757	-0.0783
	Fatigue group: fatigued	0.0157	0.1124	0.1397	0.8890		-0.2031	0.2345
	Age	-0.2766	0.0544	-5.0795	0.0000		-0.3826	-0.1705
	Sex: female	0.2279	0.1224	1.8611	0.0637		-0.0105	0.4663
	Scanner: EDI1	-0.5722	0.2700	-2.1193	0.0349		-1.0979	-0.0464
	Scanner: EDI2	-0.1852	0.2703	-0.6851	0.4938		-0.7114	0.3411
	Scanner: DUN	-0.4145	0.2993	-1.3848	0.1671		-0.9973	0.1683
	Scanner: GLA	-0.2425	0.2678	-0.9055	0.3659		-0.7640	0.2790
	DMT w1: yes	-0.0829	0.1315	-0.6302	0.5290		-0.3389	0.1732
	WML	-0.0417	0.0321	-1.3023	0.1938		-0.1042	0.0207
	Depression score	0.0073	0.0221	0.3298	0.7418		-0.0358	0.0503

	Time*Fatigue group	0.0544	0.0348	1.5639	0.1189	0.9389	-0.0133	0.1221
**Caudate**	Intercept	-0.2003	0.2181	-0.9183	0.3592		-0.6250	0.2244
	Time: w1	-0.0745	0.0381	-1.9546	0.0515		-0.1488	-0.0003
	Fatigue group: fatigued	0.2181	0.1091	1.9993	0.0465		0.0057	0.4305
	Age	-0.0686	0.0457	-1.5001	0.1346		-0.1576	0.0204
	Sex: female	0.4196	0.1022	4.1057	0.0001		0.2206	0.6186
	Scanner: EDI1	0.4872	0.2254	2.1617	0.0314		0.0483	0.9260
	Scanner: EDI2	-0.4671	0.2255	-2.0711	0.0392		-0.9062	-0.0279
	Scanner: DUN	-0.6120	0.2500	-2.4482	0.0149		-1.0989	-0.1252
	Scanner: GLA	-0.4281	0.2235	-1.9153	0.0564		-0.8633	0.0071
	DMT w1: yes	0.1107	0.1098	1.0083	0.3141		-0.1031	0.3246
	WML	-0.3258	0.0369	-8.8381	0.0000		-0.3975	-0.2540
	Depression score	-0.0477	0.0298	-1.6042	0.1097		-0.1057	0.0102
	Time*Fatigue group	-0.1268	0.0547	-2.3179	0.0211	0.5910	-0.2333	-0.0203
**GM cerebellar**	Intercept	-0.3110	0.2463	-1.2627	0.2076		-0.7907	0.1686
	Time: w1	-0.0391	0.0157	-2.4906	0.0133		-0.0697	-0.0085
	Fatigue group: fatigued	-0.1993	0.1056	-1.8866	0.0602		-0.4050	0.0064
	Age	-0.3241	0.0521	-6.2218	0.0000		-0.4255	-0.2227
	Sex: female	0.3581	0.1173	3.0535	0.0025		0.1297	0.5865
	Scanner: EDI1	0.3991	0.2587	1.5431	0.1239		-0.1045	0.9028
	Scanner: EDI2	-0.2973	0.2590	-1.1479	0.2519		-0.8015	0.2070
	Scanner: DUN	0.2228	0.2866	0.7774	0.4375		-0.3352	0.7808
	Scanner: GLA	0.1832	0.2566	0.7142	0.4756		-0.3163	0.6828
	DMT w1: yes	0.0579	0.1260	0.4593	0.6463		-0.1875	0.3032
	WML	-0.0095	0.0220	-0.4331	0.6653		-0.0525	0.0334
	Depression score	-0.0144	0.0141	-1.0146	0.3111		-0.0419	0.0132
	Time*Fatigue group	-0.0266	0.0215	-1.2344	0.2180	0.9389	-0.0685	0.0153
**GM cerebral**	Intercept	-0.5314	0.2275	-2.3353	0.0202		-0.9745	-0.0883
	Time: w1	-0.0359	0.0181	-1.9881	0.0477		-0.0711	-0.0007
	Fatigue group: fatigued	0.0442	0.0985	0.4491	0.6537		-0.1476	0.2361
	Age	-0.4295	0.0481	-8.9266	0.0000		-0.5232	-0.3358
	Sex: female	0.4203	0.1083	3.8823	0.0001		0.2095	0.6311
	Scanner: EDI1	0.7774	0.2387	3.2565	0.0013		0.3125	1.2422
	Scanner: EDI2	-0.0903	0.2390	-0.3777	0.7059		-0.5556	0.3751
	Scanner: DUN	0.2647	0.2645	1.0008	0.3177		-0.2504	0.7799
	Scanner: GLA	0.4391	0.2368	1.8545	0.0646		-0.0220	0.9002
	DMT w1: yes	-0.1799	0.1163	-1.5475	0.1228		-0.4064	0.0465
	WML	-0.0348	0.0244	-1.4274	0.1545		-0.0823	0.0127
	Depression score	-0.0063	0.0161	-0.3904	0.6965		-0.0377	0.0251
	Time*Fatigue group	-0.0283	0.0249	-1.1366	0.2566	0.9389	-0.0768	0.0202
**Hippocampus**	Intercept	0.1357	0.2664	0.5092	0.6110		-0.3831	0.6544
	Time: w1	-0.0520	0.0156	-3.3282	0.0010		-0.0825	-0.0216
	Fatigue group: fatigued	-0.0666	0.1144	-0.5820	0.5610		-0.2893	0.1562
	Age	-0.1248	0.0562	-2.2193	0.0272		-0.2344	-0.0153

	Sex: female	0.1752	0.1268	1.3815	0.1681		-0.0718	0.4222
	Scanner: EDI1	-0.2971	0.2797	-1.0621	0.2890		-0.8417	0.2476
	Scanner: EDI2	-0.3654	0.2800	-1.3048	0.1930		-0.9106	0.1799
	Scanner: DUN	-0.5540	0.3100	-1.7874	0.0749		-1.1576	0.0496
	Scanner: GLA	-0.3832	0.2775	-1.3811	0.1682		-0.9235	0.1571
	DMT w1: yes	0.2065	0.1362	1.5161	0.1305		-0.0587	0.4717
	WML	-0.0154	0.0218	-0.7037	0.4821		-0.0578	0.0271
	Depression score	0.0131	0.0139	0.9413	0.3473		-0.0140	0.0401
	Time*Fatigue group	-0.0134	0.0214	-0.6232	0.5336	0.9389	-0.0551	0.0284
**NAWM cerebral**	Intercept	0.1087	0.2515	0.4325	0.6657		-0.3809	0.5984
	Time: w1	-0.0777	0.0141	-5.5058	0.0000		-0.1052	-0.0502
	Fatigue group: fatigued	0.1172	0.1076	1.0898	0.2767		-0.0922	0.3267
	Age	-0.0447	0.0531	-0.8407	0.4012		-0.1481	0.0588
	Sex: female	0.1824	0.1198	1.5230	0.1288		-0.0508	0.4156
	Scanner: EDI1	0.1343	0.2641	0.5087	0.6114		-0.3799	0.6486
	Scanner: EDI2	-0.6425	0.2644	-2.4301	0.0157		-1.1574	-0.1277
	Scanner: DUN	-0.2721	0.2926	-0.9298	0.3532		-0.8419	0.2977
	Scanner: GLA	-0.2003	0.2620	-0.7647	0.4450		-0.7104	0.3098
	DMT w1: yes	-0.0470	0.1286	-0.3657	0.7148		-0.2975	0.2034
	WML	-0.1183	0.0199	-5.9396	0.0000		-0.1571	-0.0795
	Depression score	0.0049	0.0126	0.3921	0.6952		-0.0196	0.0295
	Time*Fatigue group	0.0013	0.0193	0.0678	0.9460	0.9997	-0.0363	0.0389
**NAWM cerebellar**	Intercept	0.0893	0.2635	0.3390	0.7348		-0.4238	0.6025
	Time: w1	-0.0063	0.0097	-0.6571	0.5116		-0.0251	0.0125
	Fatigue group: fatigued	-0.1295	0.1177	-1.1008	0.2719		-0.3586	0.0996
	Age	0.0234	0.0556	0.4208	0.6742		-0.0849	0.1317
	Sex: female	0.0111	0.1250	0.0888	0.9293		-0.2323	0.2545
	Scanner: EDI1	-0.2064	0.2758	-0.7483	0.4549		-0.7434	0.3306
	Scanner: EDI2	0.0455	0.2762	0.1647	0.8693		-0.4923	0.5832
	Scanner: DUN	-0.0707	0.3053	-0.2316	0.8170		-0.6651	0.5237
	Scanner: GLA	-0.3170	0.2735	-1.1590	0.2474		-0.8496	0.2156
	DMT w1: yes	0.1787	0.1343	1.3302	0.1844		-0.0829	0.4403
	WML	0.0192	0.0139	1.3801	0.1685		-0.0079	0.0462
	Depression score	-0.0050	0.0085	-0.5943	0.5527		-0.0215	0.0115
	Time*Fatigue group	-0.0073	0.0132	-0.5511	0.5819	0.9389	-0.0329	0.0184
**Pallidum**	Intercept	-0.0750	0.2592	-0.2895	0.7724		-0.5798	0.4297
	Time: w1	0.0092	0.0071	1.2980	0.1952		-0.0046	0.0229
	Fatigue group: fatigued	-0.1246	0.1148	-1.0856	0.2785		-0.3482	0.0989
	Age	0.0504	0.0546	0.9222	0.3571		-0.0560	0.1567
	Sex: female	-0.1707	0.1231	-1.3870	0.1665		-0.4104	0.0690
	Scanner: EDI1	0.0875	0.2715	0.3223	0.7474		-0.4411	0.6161
	Scanner: EDI2	0.2953	0.2718	1.0866	0.2781		-0.2339	0.8246
	Scanner: DUN	0.1468	0.3007	0.4883	0.6257		-0.4387	0.7324
	Scanner: GLA	0.0098	0.2693	0.0363	0.9710		-0.5146	0.5341
	DMT w1: yes	0.1998	0.1322	1.5114	0.1317		-0.0576	0.4571

	WML	-0.0120	0.0100	-1.1959	0.2327		-0.0316	0.0075
	Depression score	-0.0036	0.0061	-0.5832	0.5602		-0.0154	0.0083
	Time*Fatigue group	-0.0001	0.0096	-0.0150	0.9881	0.9997	-0.0189	0.0186
**Putamen**	Intercept	-0.4593	0.2471	-1.8591	0.0640		-0.9404	0.0218
	Time: w1	-0.0720	0.0137	-5.2597	0.0000		-0.0987	-0.0453
	Fatigue group: fatigued	0.1367	0.1056	1.2947	0.1964		-0.0689	0.3423
	Age	-0.3663	0.0522	-7.0170	0.0000		-0.4680	-0.2647
	Sex: female	0.4197	0.1177	3.5662	0.0004		0.1905	0.6488
	Scanner: EDI1	0.3004	0.2595	1.1574	0.2480		-0.2050	0.8057
	Scanner: EDI2	0.0466	0.2598	0.1793	0.8578		-0.4593	0.5525
	Scanner: DUN	0.1531	0.2875	0.5325	0.5948		-0.4068	0.7130
	Scanner: GLA	0.1251	0.2574	0.4860	0.6273		-0.3761	0.6264
	DMT w1: yes	-0.0399	0.1264	-0.3159	0.7523		-0.2860	0.2062
	WML	-0.0353	0.0194	-1.8182	0.0700		-0.0730	0.0025
	Depression score	-0.0006	0.0123	-0.0475	0.9621		-0.0245	0.0233
	Time*Fatigue group	0.0174	0.0187	0.9301	0.3530	0.9389	-0.0190	0.0538
**Thalamus**	Intercept	-0.1307	0.2161	-0.6047	0.5458		-0.5515	0.2901
	Time: w1	-0.0152	0.0026	-5.9374	0.0000		-0.0202	-0.0102
	Fatigue group: fatigued	-0.1376	0.1173	-1.1729	0.2418		-0.3660	0.0908
	Age	0.0263	0.0446	0.5896	0.5559		-0.0606	0.1133
	Sex: female	0.0951	0.1001	0.9502	0.3428		-0.0998	0.2901
	Scanner: EDI1	0.0224	0.2209	0.1013	0.9194		-0.4078	0.4525
	Scanner: EDI2	0.1777	0.2212	0.8035	0.4223		-0.2529	0.6083
	Scanner: DUN	0.0845	0.2445	0.3456	0.7299		-0.3917	0.5607
	Scanner: GLA	0.0172	0.2190	0.0787	0.9373		-0.4093	0.4438
	DMT w1: yes	0.0910	0.1075	0.8466	0.3979		-0.1184	0.3004
	WML	-0.0017	0.0031	-0.5572	0.5778		-0.0078	0.0043
	Depression score	-0.0001	0.0018	-0.0695	0.9446		-0.0037	0.0035
	Time*Fatigue group	0.0003	0.0036	0.0863	0.9313	0.9997	-0.0067	0.0073
**Ventral Diencephalon**	Intercept	0.0808	0.2614	0.3091	0.7574		-0.4283	0.5899
	Time: w1	-0.0856	0.0301	-2.8421	0.0048		-0.1443	-0.0270
	Fatigue group: fatigued	0.1368	0.1138	1.2017	0.2304		-0.0849	0.3584
	Age	-0.1696	0.0552	-3.0709	0.0023		-0.2771	-0.0621
	Sex: female	0.3039	0.1243	2.4445	0.0151		0.0618	0.5460
	Scanner: EDI1	-0.1774	0.2741	-0.6471	0.5180		-0.7111	0.3563
	Scanner: EDI2	-0.2178	0.2743	-0.7942	0.4277		-0.7519	0.3163
	Scanner: DUN	-0.3312	0.3041	-1.0894	0.2769		-0.9233	0.2608
	Scanner: GLA	-0.3719	0.2719	-1.3680	0.1723		-0.9013	0.1575
	DMT w1: yes	-0.0793	0.1334	-0.5941	0.5529		-0.3391	0.1806
	WML	-0.0262	0.0362	-0.7241	0.4695		-0.0968	0.0443
	Depression score	-0.0038	0.0262	-0.1452	0.8847		-0.0547	0.0471
	Time*Fatigue group	-0.0016	0.0423	-0.0380	0.9697	0.9997	-0.0839	0.0807
**GM**	Intercept	-0.6288	0.2472	-2.5444	0.0114		-1.1101	-0.1476
	Time: w1	-0.0185	0.0150	-1.2395	0.2161		-0.0477	0.0106

	Fatigue group: fatigued	-0.1335	0.1056	-1.2642	0.2071		-0.3391	0.0721
	Age	-0.3446	0.0523	-6.5950	0.0000		-0.4463	-0.2428
	Sex: female	0.3427	0.1177	2.9108	0.0039		0.1134	0.5719
	Scanner: EDI1	0.5705	0.2596	2.1977	0.0287		0.0650	1.0760
	Scanner: EDI2	0.6113	0.2599	2.3519	0.0193		0.1052	1.1174
	Scanner: DUN	0.4492	0.2876	1.5618	0.1194		-0.1109	1.0093
	Scanner: GLA	0.2834	0.2575	1.1004	0.2720		-0.2181	0.7848
	DMT w1: yes	0.0240	0.1264	0.1902	0.8493		-0.2221	0.2702
	WML	-0.0411	0.0211	-1.9488	0.0522		-0.0821	0.0000
	Depression score	0.0029	0.0135	0.2127	0.8317		-0.0233	0.0291
	Time*Fatigue group	-0.0250	0.0205	-1.2221	0.2226	0.9389	-0.0649	0.0148
**GM caudal anterior cingulate**	Intercept	-0.6089	0.2546	-2.3916	0.0174		-1.1047	-0.1132
	Time: w1	-0.0213	0.0128	-1.6669	0.0966		-0.0461	0.0036
	Fatigue group: fatigued	0.0819	0.1095	0.7479	0.4551		-0.1313	0.2951
	Age	-0.2190	0.0538	-4.0663	0.0001		-0.3238	-0.1141
	Sex: female	0.3728	0.1212	3.0763	0.0023		0.1368	0.6088
	Scanner: EDI1	0.6044	0.2673	2.2608	0.0245		0.0838	1.1249
	Scanner: EDI2	0.0244	0.2677	0.0910	0.9276		-0.4969	0.5456
	Scanner: DUN	0.8816	0.2960	2.9782	0.0031		0.3052	1.4581
	Scanner: GLA	0.3577	0.2652	1.3489	0.1784		-0.1586	0.8740
	DMT w1: yes	-0.0898	0.1302	-0.6893	0.4912		-0.3433	0.1638
	WML	-0.0369	0.0184	-2.0072	0.0456		-0.0728	-0.0011
	Depression score	-0.0029	0.0115	-0.2538	0.7998		-0.0253	0.0195
	Time*Fatigue group	-0.0319	0.0174	-1.8353	0.0674	0.9022	-0.0657	0.0019
**GM caudal middle frontal**	Intercept	-0.2350	0.2413	-0.9742	0.3307		-0.7048	0.2347
	Time: w1	-0.0121	0.0196	-0.6186	0.5366		-0.0502	0.0260
	Fatigue group: fatigued	0.0186	0.1044	0.1780	0.8589		-0.1847	0.2219
	Age	-0.2582	0.0510	-5.0616	0.0000		-0.3576	-0.1589
	Sex: female	0.3952	0.1148	3.4431	0.0007		0.1717	0.6187
	Scanner: EDI1	0.5408	0.2531	2.1365	0.0334		0.0479	1.0337
	Scanner: EDI2	-0.5318	0.2534	-2.0987	0.0367		-1.0252	-0.0384
	Scanner: DUN	0.0414	0.2805	0.1474	0.8829		-0.5049	0.5876
	Scanner: GLA	0.2227	0.2511	0.8870	0.3758		-0.2662	0.7116
	DMT w1: yes	-0.1927	0.1233	-1.5634	0.1190		-0.4328	0.0473
	WML	-0.0140	0.0263	-0.5331	0.5943		-0.0652	0.0372
	Depression score	-0.0079	0.0175	-0.4531	0.6508		-0.0420	0.0261
	Time*Fatigue group	-0.0482	0.0270	-1.7856	0.0751	0.9022	-0.1008	0.0044
**GM cuneus**	Intercept	-0.2286	0.2702	-0.8460	0.3982		-0.7547	0.2975
	Time: w1	-0.0387	0.0148	-2.6167	0.0093		-0.0675	-0.0099
	Fatigue group: fatigued	0.0198	0.1165	0.1697	0.8654		-0.2072	0.2467
	Age	-0.1455	0.0571	-2.5463	0.0114		-0.2568	-0.0342
	Sex: female	-0.0530	0.1286	-0.4125	0.6803		-0.3034	0.1973
	Scanner: EDI1	0.3123	0.2836	1.1013	0.2717		-0.2399	0.8646
	Scanner: EDI2	0.2257	0.2840	0.7949	0.4273		-0.3272	0.7787

	Scanner: DUN	0.0319	0.3141	0.1015	0.9192		-0.5797	0.6435
	Scanner: GLA	0.3787	0.2813	1.3463	0.1792		-0.1690	0.9265
	DMT w1: yes	0.0046	0.1381	0.0330	0.9737		-0.2644	0.2736
	WML	-0.0253	0.0212	-1.1938	0.2335		-0.0664	0.0159
	Depression score	0.0032	0.0133	0.2397	0.8107		-0.0227	0.0291
	Time*Fatigue group	0.0145	0.0202	0.7189	0.4727	0.9389	-0.0248	0.0538
**GM entorhinal**	Intercept	-0.5440	0.2644	-2.0575	0.0405		-1.0589	-0.0292
	Time: w1	-0.0007	0.0226	-0.0303	0.9758		-0.0446	0.0433
	Fatigue group: fatigued	-0.0750	0.1143	-0.6562	0.5122		-0.2975	0.1475
	Age	-0.0918	0.0559	-1.6421	0.1016		-0.2007	0.0171
	Sex: female	-0.0763	0.1258	-0.6065	0.5446		-0.3213	0.1687
	Scanner: EDI1	0.8919	0.2774	3.2150	0.0014		0.3517	1.4322
	Scanner: EDI2	0.3274	0.2777	1.1789	0.2393		-0.2134	0.8682
	Scanner: DUN	0.6943	0.3075	2.2580	0.0247		0.0955	1.2931
	Scanner: GLA	0.7257	0.2752	2.6371	0.0088		0.1898	1.2615
	DMT w1: yes	0.0120	0.1351	0.0886	0.9295		-0.2511	0.2751
	WML	-0.0428	0.0299	-1.4302	0.1537		-0.1011	0.0155
	Depression score	-0.0048	0.0201	-0.2366	0.8131		-0.0439	0.0344
	Time*Fatigue group	-0.0239	0.0312	-0.7642	0.4453	0.9389	-0.0846	0.0369
**GM frontal pole**	Intercept	-0.0457	0.2356	-0.1939	0.8464		-0.5044	0.4130
	Time: w1	-0.0231	0.0296	-0.7778	0.4373		-0.0808	0.0347
	Fatigue group: fatigued	-0.0475	0.1060	-0.4479	0.6546		-0.2540	0.1590
	Age	-0.4788	0.0497	-9.6251	0.0000		-0.5757	-0.3819
	Sex: female	0.2250	0.1117	2.0154	0.0447		0.0076	0.4425
	Scanner: EDI1	0.2110	0.2462	0.8569	0.3922		-0.2685	0.6904
	Scanner: EDI2	-0.0055	0.2464	-0.0225	0.9821		-0.4854	0.4743
	Scanner: DUN	-0.1688	0.2730	-0.6181	0.5369		-0.7005	0.3629
	Scanner: GLA	0.2021	0.2442	0.8277	0.4085		-0.2734	0.6776
	DMT w1: yes	-0.2602	0.1199	-2.1696	0.0308		-0.4937	-0.0267
	WML	-0.0151	0.0346	-0.4366	0.6627		-0.0824	0.0522
	Depression score	0.0123	0.0255	0.4849	0.6281		-0.0372	0.0619
	Time*Fatigue group	-0.0209	0.0418	-0.4998	0.6175	0.9389	-0.1022	0.0604
**GM fusiform**	Intercept	-0.6970	0.2488	-2.8014	0.0054		-1.1815	-0.2126
	Time: w1	-0.0197	0.0175	-1.1256	0.2612		-0.0537	0.0144
	Fatigue group: fatigued	-0.0347	0.1077	-0.3218	0.7478		-0.2444	0.1751
	Age	-0.2824	0.0526	-5.3658	0.0000		-0.3848	-0.1799
	Sex: female	0.1236	0.1184	1.0438	0.2974		-0.1069	0.3540
	Scanner: EDI1	1.1240	0.2611	4.3055	0.0000		0.6156	1.6323
	Scanner: EDI2	0.5159	0.2614	1.9739	0.0493		0.0070	1.0249
	Scanner: DUN	0.6615	0.2892	2.2874	0.0229		0.0984	1.2247
	Scanner: GLA	0.8659	0.2589	3.3443	0.0009		0.3617	1.3701
	DMT w1: yes	-0.1950	0.1272	-1.5332	0.1263		-0.4426	0.0527
	WML	-0.0560	0.0242	-2.3151	0.0213		-0.1030	-0.0089
	Depression score	-0.0183	0.0157	-1.1640	0.2453		-0.0488	0.0123
	Time*Fatigue group	-0.0266	0.0240	-1.1070	0.2692	0.9389	-0.0734	0.0202

GM inferior parietal	Intercept	-0.7629	0.2493	-3.0603	0.0024		-1.2482	-0.2775
	Time: w1	-0.0324	0.0150	-2.1627	0.0313		-0.0616	-0.0032
	Fatigue group: fatigued	-0.1869	0.1068	-1.7496	0.0812		-0.3949	0.0211
	Age	-0.2610	0.0527	-4.9507	0.0000		-0.3637	-0.1583
	Sex: female	0.4123	0.1187	3.4735	0.0006		0.1812	0.6434
	Scanner: EDI1	0.9321	0.2618	3.5608	0.0004		0.4224	1.4419
	Scanner: EDI2	0.4405	0.2621	1.6807	0.0938		-0.0699	0.9509
	Scanner: DUN	0.5025	0.2900	1.7327	0.0842		-0.0622	1.0672
	Scanner: GLA	0.3816	0.2597	1.4697	0.1427		-0.1240	0.8873
	DMT w1: yes	0.0596	0.1275	0.4674	0.6405		-0.1887	0.3079
	WML	-0.0276	0.0212	-1.3035	0.1934		-0.0689	0.0137
	Depression score	-0.0061	0.0135	-0.4496	0.6533		-0.0324	0.0202
	Time*Fatigue group	-0.0201	0.0205	-0.9818	0.3270	0.9389	-0.0601	0.0198
GM inferior temporal	Intercept	-0.4361	0.2494	-1.7488	0.0813		-0.9217	0.0495
	Time: w1	-0.0276	0.0177	-1.5612	0.1195		-0.0620	0.0068
	Fatigue group: fatigued	-0.1277	0.1070	-1.1944	0.2333		-0.3360	0.0805
	Age	-0.3229	0.0527	-6.1234	0.0000		-0.4256	-0.2202
	Sex: female	0.2088	0.1187	1.7580	0.0797		-0.0225	0.4400
	Scanner: EDI1	0.6944	0.2619	2.6517	0.0084		0.1845	1.2043
	Scanner: EDI2	0.0208	0.2622	0.0795	0.9367		-0.4896	0.5313
	Scanner: DUN	0.5037	0.2902	1.7358	0.0836		-0.0614	1.0687
	Scanner: GLA	0.5291	0.2597	2.0372	0.0425		0.0234	1.0349
	DMT w1: yes	-0.0880	0.1275	-0.6897	0.4909		-0.3363	0.1604
	WML	-0.0171	0.0243	-0.7018	0.4833		-0.0645	0.0303
	Depression score	-0.0063	0.0159	-0.3952	0.6929		-0.0371	0.0246
	Time*Fatigue group	-0.0183	0.0243	-0.7532	0.4519	0.9389	-0.0656	0.0290
GM insula	Intercept	0.1330	0.2495	0.5329	0.5945		-0.3529	0.6189
	Time: w1	-0.0566	0.0230	-2.4589	0.0145		-0.1015	-0.0118
	Fatigue group: fatigued	-0.0481	0.1083	-0.4440	0.6574		-0.2589	0.1628
	Age	-0.2969	0.0528	-5.6282	0.0000		-0.3997	-0.1942
	Sex: female	0.0997	0.1187	0.8399	0.4016		-0.1314	0.3308
	Scanner: EDI1	0.3060	0.2617	1.1692	0.2432		-0.2036	0.8156
	Scanner: EDI2	-0.4189	0.2620	-1.5990	0.1109		-0.9290	0.0912
	Scanner: DUN	-0.5485	0.2901	-1.8906	0.0596		-1.1135	0.0164
	Scanner: GLA	-0.2948	0.2596	-1.1357	0.2570		-0.8003	0.2107
	DMT w1: yes	0.0565	0.1275	0.4432	0.6579		-0.1917	0.3047
	WML	0.0153	0.0299	0.5119	0.6091		-0.0430	0.0736
	Depression score	-0.0015	0.0204	-0.0721	0.9425		-0.0413	0.0383
	Time*Fatigue group	0.0075	0.0320	0.2346	0.8147	0.9997	-0.0547	0.0697
GM isthmus [illegible]	Intercept	-0.1141	0.2616	-0.4361	0.6630		-0.6234	0.3952
	Time: w1	-0.0281	0.0165	-1.7006	0.0900		-0.0603	0.0041
	Fatigue group: fatigued	0.1755	0.1123	1.5631	0.1191		-0.0431	0.3941
	Age	-0.2216	0.0553	-4.0055	0.0001		-0.3293	-0.1139
	Sex: female	-0.0134	0.1245	-0.1079	0.9142		-0.2559	0.2291

	Scanner: EDI1	0.3321	0.2746	1.2091	0.2276		-0.2027	0.8668
	Scanner: EDI2	-0.2907	0.2750	-1.0574	0.2912		-0.8262	0.2447
	Scanner: DUN	-0.0859	0.3042	-0.2825	0.7778		-0.6784	0.5065
	Scanner: GLA	0.1676	0.2724	0.6154	0.5388		-0.3628	0.6981
	DMT w1: yes	-0.0007	0.1338	-0.0054	0.9957		-0.2612	0.2598
	WML	-0.0131	0.0232	-0.5648	0.5726		-0.0584	0.0321
	Depression score	-0.0060	0.0149	-0.4045	0.6862		-0.0350	0.0230
	Time*Fatigue group	-0.0146	0.0227	-0.6435	0.5204	0.9389	-0.0587	0.0295
**GM lateral occipital**	Intercept	-0.6269	0.2557	-2.4518	0.0148		-1.1248	-0.1290
	Time: w1	-0.0323	0.0189	-1.7071	0.0888		-0.0692	0.0045
	Fatigue group: fatigued	0.1066	0.1112	0.9584	0.3386		-0.1099	0.3230
	Age	-0.2741	0.0541	-5.0682	0.0000		-0.3793	-0.1688
	Sex: female	-0.0828	0.1216	-0.6808	0.4965		-0.3196	0.1540
	Scanner: EDI1	1.0611	0.2682	3.9569	0.0001		0.5389	1.5833
	Scanner: EDI2	0.5474	0.2685	2.0387	0.0423		0.0246	1.0702
	Scanner: DUN	0.8848	0.2971	2.9782	0.0031		0.3063	1.4633
	Scanner: GLA	0.9668	0.2660	3.6348	0.0003		0.4489	1.4847
	DMT w1: yes	-0.2287	0.1306	-1.7507	0.0810		-0.4831	0.0257
	WML	-0.0087	0.0259	-0.3357	0.7373		-0.0592	0.0418
	Depression score	-0.0302	0.0169	-1.7842	0.0754		-0.0632	0.0028
	Time*Fatigue group	-0.0449	0.0261	-1.7228	0.0859	0.9022	-0.0957	0.0059
**GM lateral orbitofrontal**	Intercept	-0.5767	0.2414	-2.3884	0.0175		-1.0468	-0.1065
	Time: w1	-0.0319	0.0246	-1.2986	0.1950		-0.0797	0.0159
	Fatigue group: fatigued	-0.0106	0.1047	-0.1015	0.9192		-0.2146	0.1933
	Age	-0.3853	0.0510	-7.5509	0.0000		-0.4847	-0.2859
	Sex: female	0.2116	0.1148	1.8424	0.0664		-0.0120	0.4352
	Scanner: EDI1	0.6977	0.2532	2.7554	0.0062		0.2046	1.1908
	Scanner: EDI2	0.1533	0.2534	0.6049	0.5457		-0.3402	0.6468
	Scanner: DUN	0.2438	0.2808	0.8683	0.3859		-0.3030	0.7906
	Scanner: GLA	0.4302	0.2512	1.7128	0.0878		-0.0589	0.9193
	DMT w1: yes	0.0784	0.1233	0.6358	0.5254		-0.1617	0.3185
	WML	-0.0418	0.0310	-1.3507	0.1778		-0.1021	0.0185
	Depression score	0.0003	0.0216	0.0150	0.9881		-0.0418	0.0424
	Time*Fatigue group	-0.0198	0.0342	-0.5775	0.5640	0.9389	-0.0865	0.0469
**GM lingual**	Intercept	-0.2095	0.2666	-0.7861	0.4324		-0.7286	0.3095
	Time: w1	-0.0329	0.0153	-2.1490	0.0324		-0.0627	-0.0031
	Fatigue group: fatigued	0.0819	0.1157	0.7077	0.4797		-0.1434	0.3071
	Age	-0.2035	0.0564	-3.6102	0.0004		-0.3133	-0.0937
	Sex: female	0.0916	0.1268	0.7227	0.4704		-0.1553	0.3385
	Scanner: EDI1	0.2521	0.2796	0.9014	0.3681		-0.2924	0.7966
	Scanner: EDI2	0.2038	0.2800	0.7279	0.4672		-0.3414	0.7491
	Scanner: DUN	-0.1070	0.3097	-0.3456	0.7299		-0.7100	0.4960
	Scanner: GLA	0.0883	0.2774	0.3183	0.7505		-0.4518	0.6284
	DMT w1: yes	-0.0060	0.1362	-0.0441	0.9648		-0.2713	0.2592
	WML	-0.0314	0.0217	-1.4435	0.1499		-0.0737	0.0109

	Depression score	-0.0161	0.0137	-1.1707	0.2426		-0.0428	0.0107
	Time*Fatigue group	-0.0108	0.0209	-0.5175	0.6052	0.9389	-0.0516	0.0299
GM medial orbitofrontal	Intercept	-0.1432	0.2398	-0.5973	0.5508		-0.6101	0.3237
	Time: w1	-0.0335	0.0299	-1.1229	0.2624		-0.0916	0.0246
	Fatigue group: fatigued	0.0535	0.1057	0.5057	0.6135		-0.1524	0.2593
	Age	-0.4065	0.0507	-8.0243	0.0000		-0.5051	-0.3078
	Sex: female	0.1226	0.1139	1.0769	0.2824		-0.0991	0.3444
	Scanner: EDI1	0.2507	0.2511	0.9985	0.3188		-0.2382	0.7396
	Scanner: EDI2	0.0218	0.2513	0.0867	0.9309		-0.4675	0.5111
	Scanner: DUN	-0.4564	0.2785	-1.6387	0.1023		-0.9988	0.0859
	Scanner: GLA	0.2104	0.2490	0.8448	0.3989		-0.2746	0.6953
	DMT w1: yes	-0.0669	0.1223	-0.5475	0.5844		-0.3050	0.1711
	WML	-0.0358	0.0349	-1.0233	0.3070		-0.1038	0.0323
	Depression score	0.0022	0.0258	0.0865	0.9312		-0.0479	0.0524
	Time*Fatigue group	-0.0009	0.0420	-0.0217	0.9827	0.9997	-0.0827	0.0809
GM middle temporal	Intercept	-0.4169	0.2355	-1.7699	0.0777		-0.8755	0.0417
	Time: w1	-0.0525	0.0157	-3.3321	0.0010		-0.0831	-0.0218
	Fatigue group: fatigued	-0.2414	0.1009	-2.3931	0.0173		-0.4379	-0.0450
	Age	-0.4309	0.0498	-8.6519	0.0000		-0.5279	-0.3339
	Sex: female	0.2298	0.1122	2.0492	0.0413		0.0114	0.4483
	Scanner: EDI1	0.6451	0.2473	2.6081	0.0096		0.1635	1.1267
	Scanner: EDI2	0.2040	0.2476	0.8237	0.4107		-0.2782	0.6862
	Scanner: DUN	0.1092	0.2741	0.3983	0.6907		-0.4245	0.6428
	Scanner: GLA	0.2146	0.2453	0.8748	0.3824		-0.2631	0.6924
	DMT w1: yes	0.1324	0.1205	1.0993	0.2725		-0.1022	0.3670
	WML	0.0187	0.0219	0.8531	0.3943		-0.0240	0.0613
	Depression score	-0.0072	0.0141	-0.5107	0.6099		-0.0348	0.0203
	Time*Fatigue group	-0.0233	0.0216	-1.0766	0.2825	0.9389	-0.0653	0.0188
GM paracentral	Intercept	-0.6237	0.2416	-2.5813	0.0103		-1.0942	-0.1532
	Time: w1	-0.0036	0.0242	-0.1487	0.8819		-0.0508	0.0436
	Fatigue group: fatigued	0.0337	0.1052	0.3202	0.7490		-0.1712	0.2387
	Age	-0.2557	0.0511	-5.0056	0.0000		-0.3551	-0.1562
	Sex: female	0.5810	0.1149	5.0574	0.0000		0.3573	0.8047
	Scanner: EDI1	0.6644	0.2533	2.6227	0.0092		0.1711	1.1576
	Scanner: EDI2	-0.1999	0.2535	-0.7884	0.4311		-0.6936	0.2938
	Scanner: DUN	0.3770	0.2809	1.3421	0.1806		-0.1700	0.9239
	Scanner: GLA	0.3600	0.2513	1.4328	0.1529		-0.1293	0.8493
	DMT w1: yes	-0.1401	0.1234	-1.1359	0.2569		-0.3804	0.1001
	WML	-0.0499	0.0307	-1.6237	0.1055		-0.1097	0.0099
	Depression score	-0.0080	0.0214	-0.3761	0.7071		-0.0496	0.0336
	Time*Fatigue group	-0.0270	0.0337	-0.7994	0.4247	0.9389	-0.0926	0.0387
GM [illegible]	Intercept	-0.6301	0.2535	-2.4853	0.0135		-1.1237	-0.1364
	Time: w1	-0.0515	0.0170	-3.0327	0.0026		-0.0845	-0.0184
	Fatigue group: fatigued	-0.0636	0.1085	-0.5857	0.5585		-0.2749	0.1478

	Age	-0.2135	0.0536	-3.9840	0.0001		-0.3179	-0.1092
	Sex: female	0.5870	0.1207	4.8617	0.0000		0.3519	0.8220
	Scanner: EDI1	0.3220	0.2662	1.2095	0.2274		-0.1964	0.8404
	Scanner: EDI2	0.1693	0.2665	0.6353	0.5257		-0.3497	0.6883
	Scanner: DUN	-0.1002	0.2950	-0.3398	0.7342		-0.6747	0.4742
	Scanner: GLA	0.3030	0.2641	1.1474	0.2521		-0.2112	0.8172
	DMT w1: yes	0.0323	0.1297	0.2490	0.8035		-0.2202	0.2848
	WML	-0.0504	0.0236	-2.1376	0.0333		-0.0963	-0.0045
	Depression score	-0.0011	0.0152	-0.0748	0.9405		-0.0308	0.0285
	Time*Fatigue group	0.0106	0.0233	0.4566	0.6483	0.9389	-0.0347	0.0560
**GM pars opercularis**	Intercept	-0.0232	0.2434	-0.0952	0.9242		-0.4970	0.4507
	Time: w1	-0.0348	0.0160	-2.1774	0.0302		-0.0659	-0.0037
	Fatigue group: fatigued	-0.0836	0.1043	-0.8016	0.4234		-0.2866	0.1195
	Age	-0.3889	0.0515	-7.5570	0.0000		-0.4891	-0.2887
	Sex: female	0.3700	0.1159	3.1926	0.0016		0.1443	0.5956
	Scanner: EDI1	0.0820	0.2555	0.3209	0.7485		-0.4156	0.5796
	Scanner: EDI2	-0.4181	0.2558	-1.6341	0.1033		-0.9163	0.0801
	Scanner: DUN	-0.3406	0.2831	-1.2030	0.2299		-0.8920	0.2107
	Scanner: GLA	-0.2036	0.2535	-0.8034	0.4224		-0.6972	0.2899
	DMT w1: yes	-0.0057	0.1245	-0.0461	0.9633		-0.2481	0.2366
	WML	-0.0409	0.0223	-1.8356	0.0674		-0.0844	0.0025
	Depression score	-0.0024	0.0144	-0.1676	0.8670		-0.0304	0.0256
	Time*Fatigue group	-0.0048	0.0219	-0.2176	0.8279	0.9997	-0.0474	0.0379
**GM pars orbitalis**	Intercept	-0.5793	0.2436	-2.3781	0.0180		-1.0536	-0.1050
	Time: w1	-0.0627	0.0220	-2.8526	0.0046		-0.1055	-0.0199
	Fatigue group: fatigued	-0.0018	0.1059	-0.0171	0.9864		-0.2080	0.2044
	Age	-0.4256	0.0515	-8.2635	0.0000		-0.5259	-0.3253
	Sex: female	0.1858	0.1158	1.6042	0.1097		-0.0397	0.4114
	Scanner: EDI1	0.7153	0.2554	2.8003	0.0054		0.2179	1.2127
	Scanner: EDI2	0.4588	0.2557	1.7941	0.0738		-0.0392	0.9567
	Scanner: DUN	0.4442	0.2831	1.5688	0.1177		-0.1072	0.9955
	Scanner: GLA	0.4543	0.2534	1.7929	0.0740		-0.0391	0.9476
	DMT w1: yes	-0.0269	0.1244	-0.2158	0.8293		-0.2691	0.2154
	WML	0.0179	0.0288	0.6213	0.5349		-0.0382	0.0739
	Depression score	-0.0164	0.0195	-0.8417	0.4006		-0.0545	0.0216
	Time*Fatigue group	0.0000	0.0305	0.0016	0.9987	0.9997	-0.0593	0.0594
**GM pars triangularis**	Intercept	-0.1691	0.2518	-0.6718	0.5022		-0.6594	0.3211
	Time: w1	-0.0394	0.0174	-2.2717	0.0238		-0.0732	-0.0056
	Fatigue group: fatigued	0.0651	0.1089	0.5979	0.5504		-0.1469	0.2771
	Age	-0.3693	0.0533	-6.9356	0.0000		-0.4730	-0.2656
	Sex: female	0.1088	0.1198	0.9079	0.3647		-0.1245	0.3421
	Scanner: EDI1	0.4479	0.2642	1.6952	0.0911		-0.0666	0.9624
	Scanner: EDI2	-0.0540	0.2645	-0.2040	0.8385		-0.5691	0.4611
	Scanner: DUN	-0.0137	0.2927	-0.0467	0.9628		-0.5836	0.5563
	Scanner: GLA	0.1430	0.2621	0.5457	0.5857		-0.3673	0.6533

	DMT w1: yes	-0.0831	0.1287	-0.6453	0.5192		-0.3337	0.1676
	WML	0.0065	0.0241	0.2705	0.7870		-0.0404	0.0534
	Depression score	0.0038	0.0156	0.2450	0.8066		-0.0265	0.0341
	Time*Fatigue group	-0.0161	0.0238	-0.6761	0.4995	0.9389	-0.0625	0.0303
**GM pericalcarine**	Intercept	-0.5651	0.2649	-2.1335	0.0337		-1.0809	-0.0494
	Time: w1	-0.0179	0.0186	-0.9624	0.3366		-0.0541	0.0183
	Fatigue group: fatigued	-0.0380	0.1153	-0.3291	0.7423		-0.2626	0.1867
	Age	-0.1273	0.0560	-2.2735	0.0237		-0.2364	-0.0183
	Sex: female	0.2218	0.1259	1.7610	0.0792		-0.0235	0.4670
	Scanner: EDI1	0.3737	0.2778	1.3452	0.1796		-0.1672	0.9146
	Scanner: EDI2	0.5831	0.2781	2.0966	0.0369		0.0415	1.1246
	Scanner: DUN	0.0313	0.3077	0.1016	0.9191		-0.5679	0.6304
	Scanner: GLA	0.2312	0.2755	0.8392	0.4020		-0.3053	0.7677
	DMT w1: yes	0.1540	0.1353	1.1381	0.2560		-0.1095	0.4175
	WML	-0.0775	0.0257	-3.0202	0.0027		-0.1275	-0.0275
	Depression score	-0.0123	0.0166	-0.7400	0.4598		-0.0447	0.0201
	Time*Fatigue group	-0.0052	0.0256	-0.2025	0.8397	0.9997	-0.0549	0.0446
**GM postcentral**	Intercept	-0.5734	0.2475	-2.3170	0.0212		-1.0553	-0.0915
	Time: w1	-0.0008	0.0258	-0.0302	0.9759		-0.0511	0.0495
	Fatigue group: fatigued	-0.0825	0.1085	-0.7606	0.4475		-0.2937	0.1287
	Age	-0.2344	0.0523	-4.4800	0.0000		-0.3362	-0.1325
	Sex: female	0.2545	0.1176	2.1641	0.0312		0.0255	0.4835
	Scanner: EDI1	1.0073	0.2593	3.8843	0.0001		0.5023	1.5122
	Scanner: EDI2	0.1862	0.2595	0.7175	0.4736		-0.3192	0.6916
	Scanner: DUN	0.4111	0.2875	1.4299	0.1538		-0.1487	0.9710
	Scanner: GLA	0.4250	0.2572	1.6524	0.0995		-0.0758	0.9258
	DMT w1: yes	-0.0783	0.1263	-0.6200	0.5357		-0.3242	0.1676
	WML	-0.0879	0.0324	-2.7143	0.0070		-0.1509	-0.0248
	Depression score	0.0034	0.0227	0.1489	0.8817		-0.0409	0.0476
	Time*Fatigue group	-0.0080	0.0361	-0.2217	0.8247	0.9997	-0.0782	0.0622
**GM posterior cingulate**	Intercept	-0.1720	0.2434	-0.7065	0.4804		-0.6460	0.3020
	Time: w1	-0.0221	0.0164	-1.3494	0.1782		-0.0540	0.0098
	Fatigue group: fatigued	0.1600	0.1044	1.5324	0.1265		-0.0433	0.3634
	Age	-0.3881	0.0515	-7.5396	0.0000		-0.4884	-0.2879
	Sex: female	0.1539	0.1159	1.3278	0.1852		-0.0718	0.3796
	Scanner: EDI1	0.5224	0.2556	2.0437	0.0418		0.0247	1.0201
	Scanner: EDI2	-0.2061	0.2559	-0.8054	0.4212		-0.7044	0.2922
	Scanner: DUN	0.1985	0.2832	0.7007	0.4840		-0.3530	0.7499
	Scanner: GLA	0.0871	0.2535	0.3434	0.7315		-0.4066	0.5808
	DMT w1: yes	-0.1707	0.1245	-1.3710	0.1714		-0.4131	0.0717
	WML	-0.0596	0.0228	-2.6183	0.0093		-0.1040	-0.0153
	Depression score	-0.0048	0.0147	-0.3227	0.7471		-0.0334	0.0239
	Time*Fatigue group	-0.0223	0.0225	-0.9928	0.3216	0.9389	-0.0661	0.0214
**GM**	Intercept	-0.5091	0.2364	-2.1536	0.0320		-0.9694	-0.0488
	Time: w1	0.0048	0.0257	0.1879	0.8511		-0.0452	0.0548

	Fatigue group: fatigued	0.0169	0.1043	0.1619	0.8715		-0.1863	0.2201
	Age	-0.2739	0.0500	-5.4825	0.0000		-0.3712	-0.1766
	Sex: female	0.3212	0.1123	2.8608	0.0045		0.1026	0.5398
	Scanner: EDI1	1.0323	0.2476	4.1700	0.0000		0.5503	1.5144
	Scanner: EDI2	-0.0307	0.2478	-0.1238	0.9016		-0.5132	0.4518
	Scanner: DUN	0.5930	0.2745	2.1604	0.0315		0.0585	1.1275
	Scanner: GLA	0.6272	0.2455	2.5544	0.0111		0.1491	1.1053
	DMT w1: yes	-0.3685	0.1206	-3.0565	0.0024		-0.6033	-0.1338
	WML	-0.0494	0.0317	-1.5554	0.1209		-0.1111	0.0124
	Depression score	-0.0131	0.0225	-0.5814	0.5614		-0.0568	0.0307
	Time*Fatigue group	-0.0139	0.0359	-0.3886	0.6978	0.9935	-0.0838	0.0559
**GM precuneus**	Intercept	-0.1805	0.2562	-0.7044	0.4817		-0.6793	0.3184
	Time: w1	-0.0196	0.0206	-0.9514	0.3422		-0.0598	0.0205
	Fatigue group: fatigued	-0.0343	0.1114	-0.3077	0.7585		-0.2512	0.1827
	Age	-0.2634	0.0542	-4.8630	0.0000		-0.3689	-0.1580
	Sex: female	0.2579	0.1218	2.1172	0.0351		0.0207	0.4951
	Scanner: EDI1	0.3920	0.2687	1.4593	0.1455		-0.1311	0.9152
	Scanner: EDI2	-0.0907	0.2690	-0.3373	0.7361		-0.6145	0.4330
	Scanner: DUN	-0.2285	0.2977	-0.7676	0.4433		-0.8082	0.3512
	Scanner: GLA	0.1099	0.2665	0.4123	0.6804		-0.4090	0.6288
	DMT w1: yes	-0.0841	0.1309	-0.6424	0.5211		-0.3389	0.1708
	WML	-0.0392	0.0277	-1.4128	0.1587		-0.0932	0.0148
	Depression score	-0.0101	0.0184	-0.5494	0.5831		-0.0459	0.0257
	Time*Fatigue group	-0.0491	0.0285	-1.7242	0.0857	0.9022	-0.1045	0.0063
**GM rostral anterior cingulate**	Intercept	-0.3912	0.2525	-1.5493	0.1223		-0.8829	0.1005
	Time: w1	-0.0280	0.0168	-1.6715	0.0956		-0.0606	0.0046
	Fatigue group: fatigued	-0.0894	0.1082	-0.8259	0.4095		-0.3000	0.1213
	Age	-0.3102	0.0534	-5.8103	0.0000		-0.4142	-0.2063
	Sex: female	-0.0052	0.1202	-0.0430	0.9658		-0.2393	0.2290
	Scanner: EDI1	0.6573	0.2652	2.4787	0.0137		0.1409	1.1737
	Scanner: EDI2	0.1550	0.2655	0.5838	0.5598		-0.3620	0.6720
	Scanner: DUN	0.7291	0.2938	2.4814	0.0136		0.1569	1.3012
	Scanner: GLA	0.4783	0.2630	1.8184	0.0700		-0.0339	0.9905
	DMT w1: yes	0.0089	0.1291	0.0691	0.9449		-0.2426	0.2604
	WML	-0.0378	0.0233	-1.6194	0.1064		-0.0833	0.0077
	Depression score	-0.0219	0.0151	-1.4547	0.1468		-0.0512	0.0074
	Time*Fatigue group	-0.0209	0.0230	-0.9077	0.3647	0.9389	-0.0656	0.0239
**GM rostral middle frontal**	Intercept	-0.3354	0.2420	-1.3859	0.1668		-0.8066	0.1358
	Time: w1	-0.0217	0.0209	-1.0378	0.3002		-0.0623	0.0190
	Fatigue group: fatigued	-0.0470	0.1050	-0.4476	0.6548		-0.2515	0.1575
	Age	-0.3832	0.0512	-7.4886	0.0000		-0.4828	-0.2836
	Sex: female	-0.0304	0.1151	-0.2645	0.7916		-0.2546	0.1937
	Scanner: EDI1	0.6937	0.2538	2.7329	0.0066		0.1994	1.1879
	Scanner: EDI2	0.0204	0.2541	0.0802	0.9361		-0.4744	0.5152

	Scanner: DUN	0.3061	0.2813	1.0880	0.2775		-0.2417	0.8539
	Scanner: GLA	0.5057	0.2518	2.0086	0.0455		0.0155	0.9959
	DMT w1: yes	0.0114	0.1236	0.0922	0.9266		-0.2293	0.2521
	WML	-0.0348	0.0276	-1.2605	0.2084		-0.0887	0.0190
	Depression score	0.0038	0.0186	0.2045	0.8381		-0.0324	0.0400
	Time*Fatigue group	-0.0317	0.0289	-1.0974	0.2733	0.9389	-0.0880	0.0246
**GM superior frontal**	Intercept	-0.0827	0.2187	-0.3783	0.7055		-0.5086	0.3431
	Time: w1	-0.0300	0.0283	-1.0602	0.2899		-0.0850	0.0251
	Fatigue group: fatigued	0.0321	0.0968	0.3319	0.7402		-0.1564	0.2207
	Age	-0.4071	0.0462	-8.8111	0.0000		-0.4970	-0.3171
	Sex: female	0.2066	0.1038	1.9891	0.0476		0.0043	0.4088
	Scanner: EDI1	0.5440	0.2289	2.3763	0.0181		0.0982	0.9898
	Scanner: EDI2	-0.6780	0.2291	-2.9594	0.0033		-1.1241	-0.2319
	Scanner: DUN	-0.2160	0.2540	-0.8503	0.3958		-0.7105	0.2786
	Scanner: GLA	0.0446	0.2271	0.1966	0.8443		-0.3975	0.4868
	DMT w1: yes	-0.0279	0.1115	-0.2506	0.8023		-0.2450	0.1891
	WML	-0.0382	0.0326	-1.1720	0.2421		-0.1016	0.0252
	Depression score	-0.0139	0.0243	-0.5736	0.5666		-0.0612	0.0333
	Time*Fatigue group	-0.0456	0.0399	-1.1428	0.2540	0.9389	-0.1232	0.0321
**GM superior parietal**	Intercept	-0.7621	0.2515	-3.0303	0.0026		-1.2518	-0.2724
	Time: w1	-0.0222	0.0239	-0.9319	0.3521		-0.0687	0.0242
	Fatigue group: fatigued	-0.0685	0.1098	-0.6239	0.5331		-0.2822	0.1453
	Age	-0.2055	0.0532	-3.8646	0.0001		-0.3090	-0.1019
	Sex: female	0.4751	0.1196	3.9742	0.0001		0.2423	0.7079
	Scanner: EDI1	0.7963	0.2636	3.0207	0.0027		0.2830	1.3097
	Scanner: EDI2	0.2308	0.2639	0.8747	0.3825		-0.2830	0.7447
	Scanner: DUN	0.2290	0.2922	0.7835	0.4339		-0.3401	0.7980
	Scanner: GLA	0.5905	0.2615	2.2583	0.0246		0.0813	1.0996
	DMT w1: yes	-0.0462	0.1284	-0.3598	0.7193		-0.2962	0.2038
	WML	-0.0493	0.0308	-1.5999	0.1106		-0.1093	0.0107
	Depression score	0.0071	0.0211	0.3364	0.7368		-0.0340	0.0483
	Time*Fatigue group	-0.0187	0.0332	-0.5626	0.5741	0.9389	-0.0832	0.0459
**GM superior temporal**	Intercept	-0.3592	0.2391	-1.5022	0.1341		-0.8249	0.1064
	Time: w1	-0.0577	0.0195	-2.9659	0.0033		-0.0956	-0.0198
	Fatigue group: fatigued	-0.0476	0.1046	-0.4554	0.6491		-0.2512	0.1560
	Age	-0.4473	0.0506	-8.8450	0.0000		-0.5457	-0.3488
	Sex: female	0.3627	0.1137	3.1909	0.0016		0.1414	0.5840
	Scanner: EDI1	0.4412	0.2507	1.7602	0.0794		-0.0469	0.9294
	Scanner: EDI2	0.2814	0.2510	1.1212	0.2631		-0.2073	0.7701
	Scanner: DUN	0.2198	0.2778	0.7915	0.4293		-0.3210	0.7607
	Scanner: GLA	0.2029	0.2486	0.8162	0.4150		-0.2812	0.6871
	DMT w1: yes	-0.1768	0.1221	-1.4476	0.1488		-0.4146	0.0610
	WML	-0.0248	0.0261	-0.9491	0.3433		-0.0756	0.0260
	Depression score	-0.0049	0.0173	-0.2806	0.7792		-0.0386	0.0289
	Time*Fatigue group	-0.0005	0.0269	-0.0194	0.9845	0.9997	-0.0528	0.0518

**GM supramarginal**	Intercept	-0.1779	0.2513	-0.7080	0.4795		-0.6671	0.3113
	Time: w1	-0.0264	0.0179	-1.4758	0.1410		-0.0613	0.0084
	Fatigue group: fatigued	0.0094	0.1087	0.0861	0.9314		-0.2024	0.2211
	Age	-0.3580	0.0531	-6.7379	0.0000		-0.4615	-0.2546
	Sex: female	0.1120	0.1195	0.9374	0.3493		-0.1207	0.3448
	Scanner: EDI1	0.4036	0.2636	1.5309	0.1268		-0.1098	0.9169
	Scanner: EDI2	-0.0159	0.2639	-0.0602	0.9520		-0.5298	0.4981
	Scanner: DUN	-0.0805	0.2921	-0.2755	0.7831		-0.6492	0.4882
	Scanner: GLA	0.0282	0.2615	0.1078	0.9142		-0.4810	0.5374
	DMT w1: yes	0.0270	0.1284	0.2100	0.8338		-0.2231	0.2770
	WML	-0.0362	0.0247	-1.4659	0.1437		-0.0843	0.0119
	Depression score	-0.0042	0.0161	-0.2630	0.7928		-0.0355	0.0270
	Time*Fatigue group	-0.0308	0.0246	-1.2532	0.2111	0.9389	-0.0788	0.0171
**GM temporal pole**	Intercept	-0.0353	0.2643	-0.1335	0.8939		-0.5498	0.4792
	Time: w1	-0.0157	0.0280	-0.5602	0.5758		-0.0701	0.0388
	Fatigue group: fatigued	-0.0535	0.1155	-0.4630	0.6437		-0.2783	0.1714
	Age	-0.0686	0.0559	-1.2275	0.2206		-0.1773	0.0402
	Sex: female	0.4243	0.1256	3.3778	0.0008		0.1797	0.6689
	Scanner: EDI1	-0.0279	0.2770	-0.1007	0.9199		-0.5672	0.5114
	Scanner: EDI2	-0.1703	0.2772	-0.6142	0.5395		-0.7100	0.3695
	Scanner: DUN	-0.3757	0.3071	-1.2234	0.2221		-0.9737	0.2223
	Scanner: GLA	-0.1280	0.2747	-0.4659	0.6416		-0.6629	0.4069
	DMT w1: yes	-0.1539	0.1349	-1.1411	0.2547		-0.4166	0.1087
	WML	-0.0881	0.0349	-2.5286	0.0120		-0.1560	-0.0203
	Depression score	0.0171	0.0246	0.6969	0.4864		-0.0307	0.0650
	Time*Fatigue group	-0.0096	0.0391	-0.2445	0.8070	0.9997	-0.0856	0.0665
**GM transverse temporal**	Intercept	-0.2287	0.2579	-0.8868	0.3759		-0.7308	0.2734
	Time: w1	-0.0732	0.0193	-3.8003	0.0002		-0.1108	-0.0357
	Fatigue group: fatigued	0.2180	0.1125	1.9379	0.0536		-0.0011	0.4371
	Age	-0.2765	0.0545	-5.0701	0.0000		-0.3827	-0.1703
	Sex: female	0.3907	0.1226	3.1873	0.0016		0.1520	0.6294
	Scanner: EDI1	0.1021	0.2704	0.3777	0.7059		-0.4244	0.6286
	Scanner: EDI2	0.0272	0.2707	0.1004	0.9201		-0.4999	0.5543
	Scanner: DUN	0.2068	0.2995	0.6905	0.4904		-0.3764	0.7901
	Scanner: GLA	-0.1220	0.2682	-0.4549	0.6495		-0.6442	0.4002
	DMT w1: yes	-0.1838	0.1317	-1.3959	0.1638		-0.4403	0.0726
	WML	-0.0333	0.0263	-1.2675	0.2059		-0.0846	0.0179
	Depression score	-0.0226	0.0172	-1.3163	0.1891		-0.0562	0.0109
	Time*Fatigue group	0.0130	0.0265	0.4886	0.6255	0.9389	-0.0387	0.0646
**NAWM banks sts**	Intercept	-0.5551	0.2631	-2.1094	0.0357		-1.0675	-0.0427
	Time: w1	-0.0211	0.0103	-2.0583	0.0404		-0.0412	-0.0011
	Fatigue group: fatigued	-0.1789	0.1120	-1.5974	0.1112		-0.3970	0.0392
	Age	-0.0718	0.0556	-1.2906	0.1978		-0.1802	0.0365
	Sex: female	0.4390	0.1254	3.5012	0.0005		0.1948	0.6831

	Scanner: EDI1	0.2839	0.2765	1.0266	0.3054		-0.2546	0.8223
	Scanner: EDI2	0.3675	0.2769	1.3274	0.1854		-0.1717	0.9067
	Scanner: DUN	0.3693	0.3062	1.2061	0.2287		-0.2270	0.9656
	Scanner: GLA	0.0983	0.2743	0.3585	0.7202		-0.4358	0.6325
	DMT w1: yes	0.1186	0.1347	0.8804	0.3793		-0.1437	0.3809
	WML	-0.0740	0.0151	-4.8993	0.0000		-0.1034	-0.0446
	Depression score	0.0075	0.0093	0.8070	0.4203		-0.0106	0.0256
	Time*Fatigue group	0.0095	0.0139	0.6817	0.4959	0.9389	-0.0176	0.0367
**NAWM caudal anterior cingulate**	Intercept	-0.3938	0.2447	-1.6091	0.1086		-0.8704	0.0827
	Time: w1	-0.0690	0.0173	-3.9777	0.0001		-0.1027	-0.0352
	Fatigue group: fatigued	0.0297	0.1048	0.2831	0.7773		-0.1744	0.2338
	Age	-0.0819	0.0517	-1.5825	0.1146		-0.1826	0.0189
	Sex: female	0.3753	0.1165	3.2198	0.0014		0.1483	0.6022
	Scanner: EDI1	0.5709	0.2570	2.2215	0.0271		0.0705	1.0714
	Scanner: EDI2	-0.1984	0.2573	-0.7713	0.4411		-0.6994	0.3025
	Scanner: DUN	0.5835	0.2848	2.0486	0.0414		0.0289	1.1381
	Scanner: GLA	-0.0061	0.2549	-0.0238	0.9811		-0.5025	0.4903
	DMT w1: yes	-0.0188	0.1252	-0.1501	0.8808		-0.2625	0.2249
	WML	-0.1269	0.0238	-5.3326	0.0000		-0.1732	-0.0806
	Depression score	-0.0093	0.0155	-0.5971	0.5509		-0.0394	0.0209
	Time*Fatigue group	0.0116	0.0238	0.4848	0.6282	0.9389	-0.0349	0.0580
**NAWM caudal middle frontal**	Intercept	0.0325	0.2548	0.1274	0.8987		-0.4637	0.5287
	Time: w1	-0.0039	0.0133	-0.2965	0.7670		-0.0298	0.0219
	Fatigue group: fatigued	-0.0449	0.1087	-0.4135	0.6795		-0.2566	0.1667
	Age	-0.0129	0.0539	-0.2394	0.8110		-0.1178	0.0920
	Sex: female	0.4929	0.1214	4.0604	0.0001		0.2565	0.7293
	Scanner: EDI1	-0.0038	0.2677	-0.0140	0.9888		-0.5251	0.5176
	Scanner: EDI2	-0.8246	0.2680	-3.0762	0.0023		-1.3465	-0.3026
	Scanner: DUN	-0.3446	0.2966	-1.1618	0.2462		-0.9221	0.2330
	Scanner: GLA	-0.3190	0.2656	-1.2011	0.2306		-0.8361	0.1981
	DMT w1: yes	-0.0335	0.1304	-0.2566	0.7976		-0.2873	0.2204
	WML	-0.0683	0.0190	-3.5953	0.0004		-0.1053	-0.0313
	Depression score	-0.0058	0.0119	-0.4884	0.6256		-0.0291	0.0174
	Time*Fatigue group	-0.0120	0.0181	-0.6633	0.5076	0.9389	-0.0473	0.0233
**NAWM cuneus**	Intercept	0.4404	0.2681	1.6423	0.1015		-0.0817	0.9625
	Time: w1	0.0351	0.0197	1.7828	0.0756		-0.0032	0.0734
	Fatigue group: fatigued	0.0934	0.1149	0.8124	0.4172		-0.1304	0.3171
	Age	0.0511	0.0567	0.9015	0.3680		-0.0593	0.1614
	Sex: female	-0.1745	0.1277	-1.3670	0.1726		-0.4232	0.0741
	Scanner: EDI1	-0.0940	0.2816	-0.3337	0.7388		-0.6422	0.4543
	Scanner: EDI2	-0.4588	0.2819	-1.6278	0.1046		-1.0076	0.0900
	Scanner: DUN	-0.2753	0.3121	-0.8821	0.3784		-0.8829	0.3324
	Scanner: GLA	-0.1489	0.2793	-0.5331	0.5943		-0.6927	0.3949
	DMT w1: yes	-0.2026	0.1371	-1.4778	0.1405		-0.4696	0.0644
	WML	-0.1203	0.0268	-4.4847	0.0000		-0.1725	-0.0680

	Depression score	0.0222	0.0176	1.2653	0.2067		-0.0120	0.0564
	Time*Fatigue group	-0.0222	0.0271	-0.8180	0.4140	0.9389	-0.0749	0.0306
**NAWM entorhinal**	Intercept	-0.3562	0.2710	-1.3144	0.1897		-0.8839	0.1715
	Time: w1	-0.0470	0.0206	-2.2799	0.0233		-0.0872	-0.0069
	Fatigue group: fatigued	-0.1169	0.1184	-0.9876	0.3242		-0.3475	0.1136
	Age	0.0128	0.0573	0.2237	0.8231		-0.0988	0.1244
	Sex: female	0.0181	0.1288	0.1404	0.8884		-0.2328	0.2689
	Scanner: EDI1	0.5525	0.2841	1.9446	0.0527		-0.0007	1.1057
	Scanner: EDI2	0.2284	0.2844	0.8029	0.4227		-0.3255	0.7823
	Scanner: DUN	0.5167	0.3147	1.6418	0.1017		-0.0961	1.1296
	Scanner: GLA	0.2855	0.2818	1.0132	0.3118		-0.2632	0.8342
	DMT w1: yes	0.1021	0.1384	0.7378	0.4612		-0.1674	0.3716
	WML	-0.0116	0.0281	-0.4150	0.6784		-0.0663	0.0430
	Depression score	-0.0043	0.0184	-0.2325	0.8163		-0.0401	0.0316
	Time*Fatigue group	0.0000	0.0284	-0.0004	0.9997	0.9997	-0.0554	0.0553
**NAWM frontal pole**	Intercept	0.1732	0.2639	0.6563	0.5121		-0.3406	0.6870
	Time: w1	0.0344	0.0312	1.1028	0.2710		-0.0263	0.0951
	Fatigue group: fatigued	0.0651	0.1153	0.5647	0.5727		-0.1594	0.2897
	Age	0.0200	0.0557	0.3587	0.7201		-0.0886	0.1286
	Sex: female	0.2033	0.1254	1.6211	0.1060		-0.0409	0.4476
	Scanner: EDI1	-0.2526	0.2765	-0.9133	0.3618		-0.7911	0.2859
	Scanner: EDI2	-0.6022	0.2767	-2.1762	0.0303		-1.1411	-0.0634
	Scanner: DUN	-0.7479	0.3068	-2.4379	0.0153		-1.3453	-0.1505
	Scanner: GLA	-0.1592	0.2743	-0.5806	0.5620		-0.6934	0.3749
	DMT w1: yes	-0.0240	0.1347	-0.1782	0.8587		-0.2862	0.2382
	WML	-0.0470	0.0372	-1.2630	0.2075		-0.1195	0.0255
	Depression score	-0.0148	0.0271	-0.5459	0.5856		-0.0674	0.0379
	Time*Fatigue group	-0.0659	0.0438	-1.5035	0.1337	0.9389	-0.1512	0.0194
**NAWM fusiform**	Intercept	-0.3745	0.2606	-1.4370	0.1517		-0.8820	0.1329
	Time: w1	-0.0755	0.0147	-5.1355	0.0000		-0.1041	-0.0469
	Fatigue group: fatigued	0.0204	0.1116	0.1832	0.8547		-0.1968	0.2377
	Age	-0.0957	0.0550	-1.7388	0.0831		-0.2029	0.0115
	Sex: female	0.2989	0.1241	2.4082	0.0166		0.0572	0.5406
	Scanner: EDI1	0.3526	0.2737	1.2882	0.1987		-0.1804	0.8855
	Scanner: EDI2	0.0339	0.2740	0.1237	0.9016		-0.4997	0.5675
	Scanner: DUN	-0.0227	0.3033	-0.0749	0.9403		-0.6133	0.5678
	Scanner: GLA	0.2039	0.2715	0.7512	0.4531		-0.3247	0.7326
	DMT w1: yes	0.0209	0.1333	0.1566	0.8756		-0.2386	0.2804
	WML	-0.0655	0.0207	-3.1647	0.0017		-0.1058	-0.0252
	Depression score	-0.0011	0.0131	-0.0866	0.9311		-0.0267	0.0244
	Time*Fatigue group	-0.0017	0.0201	-0.0831	0.9338	0.9997	-0.0408	0.0375
**NAWM [illegible]**	Intercept	-0.2589	0.2633	-0.9834	0.3262		-0.7715	0.2537
	Time: w1	-0.0365	0.0121	-3.0188	0.0027		-0.0600	-0.0130
	Fatigue group: fatigued	-0.1869	0.1124	-1.6632	0.0973		-0.4058	0.0319

	Age	0.0300	0.0556	0.5397	0.5898		-0.0783	0.1383
	Sex: female	0.0967	0.1254	0.7711	0.4412		-0.1475	0.3409
	Scanner: EDI1	0.4060	0.2766	1.4680	0.1431		-0.1325	0.9445
	Scanner: EDI2	0.0536	0.2769	0.1937	0.8465		-0.4856	0.5928
	Scanner: DUN	0.0353	0.3064	0.1153	0.9083		-0.5612	0.6319
	Scanner: GLA	-0.1174	0.2743	-0.4279	0.6690		-0.6516	0.4168
	DMT w1: yes	0.3043	0.1347	2.2597	0.0245		0.0421	0.5666
	WML	-0.0663	0.0174	-3.8025	0.0002		-0.1002	-0.0323
	Depression score	0.0114	0.0109	1.0506	0.2942		-0.0097	0.0325
	Time*Fatigue group	0.0099	0.0165	0.6035	0.5466	0.9389	-0.0221	0.0420
**NAWM inferior temporal**	Intercept	0.0105	0.2653	0.0397	0.9684		-0.5060	0.5270
	Time: w1	-0.0572	0.0134	-4.2672	0.0000		-0.0832	-0.0311
	Fatigue group: fatigued	-0.0611	0.1132	-0.5397	0.5898		-0.2816	0.1594
	Age	-0.0808	0.0561	-1.4407	0.1507		-0.1899	0.0284
	Sex: female	0.0148	0.1264	0.1168	0.9071		-0.2313	0.2608
	Scanner: EDI1	0.4372	0.2786	1.5689	0.1177		-0.1054	0.9798
	Scanner: EDI2	-0.2973	0.2790	-1.0655	0.2875		-0.8405	0.2460
	Scanner: DUN	-0.1522	0.3087	-0.4929	0.6224		-0.7533	0.4490
	Scanner: GLA	-0.0696	0.2764	-0.2516	0.8015		-0.6078	0.4687
	DMT w1: yes	0.0658	0.1357	0.4848	0.6281		-0.1985	0.3300
	WML	-0.0067	0.0192	-0.3500	0.7266		-0.0440	0.0306
	Depression score	-0.0073	0.0120	-0.6048	0.5458		-0.0307	0.0161
	Time*Fatigue group	-0.0013	0.0183	-0.0695	0.9446	0.9997	-0.0369	0.0343
**NAWM insula**	Intercept	0.1841	0.2476	0.7436	0.4577		-0.2980	0.6661
	Time: w1	0.0296	0.0185	1.6052	0.1095		-0.0063	0.0656
	Fatigue group: fatigued	0.0036	0.1079	0.0338	0.9731		-0.2064	0.2137
	Age	0.0344	0.0524	0.6570	0.5117		-0.0675	0.1363
	Sex: female	0.2792	0.1177	2.3719	0.0183		0.0500	0.5084
	Scanner: EDI1	0.2213	0.2596	0.8526	0.3946		-0.2842	0.7268
	Scanner: EDI2	-0.6230	0.2599	-2.3973	0.0171		-1.1291	-0.1170
	Scanner: DUN	-0.5448	0.2876	-1.8944	0.0591		-1.1048	0.0152
	Scanner: GLA	-0.5206	0.2575	-2.0221	0.0440		-1.0220	-0.0193
	DMT w1: yes	-0.0792	0.1265	-0.6266	0.5314		-0.3255	0.1670
	WML	-0.2010	0.0252	-7.9744	0.0000		-0.2501	-0.1519
	Depression score	0.0277	0.0165	1.6824	0.0935		-0.0044	0.0599
	Time*Fatigue group	0.0087	0.0254	0.3408	0.7335	0.9997	-0.0408	0.0581
**NAWM isthmus cingulate**	Intercept	0.2013	0.2531	0.7953	0.4270		-0.2915	0.6941
	Time: w1	-0.0555	0.0154	-3.6099	0.0004		-0.0855	-0.0256
	Fatigue group: fatigued	0.2346	0.1095	2.1425	0.0329		0.0214	0.4479
	Age	0.0512	0.0534	0.9592	0.3382		-0.0527	0.1552
	Sex: female	0.2758	0.1204	2.2897	0.0227		0.0412	0.5103
	Scanner: EDI1	-0.3479	0.2656	-1.3099	0.1912		-0.8650	0.1692
	Scanner: EDI2	-0.6416	0.2658	-2.4133	0.0164		-1.1592	-0.1239
	Scanner: DUN	-0.5826	0.2943	-1.9793	0.0487		-1.1557	-0.0094
	Scanner: GLA	-0.4566	0.2634	-1.7332	0.0841		-0.9695	0.0564

	DMT w1: yes	-0.0221	0.1293	-0.1707	0.8646		-0.2738	0.2297
	WML	-0.0807	0.0211	-3.8194	0.0002		-0.1219	-0.0396
	Depression score	0.0127	0.0135	0.9376	0.3492		-0.0136	0.0389
	Time*Fatigue group	-0.0174	0.0211	-0.8221	0.4116	0.9389	-0.0586	0.0238
NAWM lateral occipital	Intercept	-0.0795	0.2662	-0.2986	0.7655		-0.5979	0.4389
	Time: w1	-0.0476	0.0191	-2.4926	0.0132		-0.0849	-0.0104
	Fatigue group: fatigued	0.0748	0.1145	0.6533	0.5140		-0.1482	0.2979
	Age	-0.1184	0.0563	-2.1032	0.0363		-0.2280	-0.0088
	Sex: female	-0.2039	0.1267	-1.6092	0.1086		-0.4507	0.0428
	Scanner: EDI1	0.5762	0.2795	2.0617	0.0401		0.0320	1.1203
	Scanner: EDI2	0.0749	0.2798	0.2679	0.7890		-0.4699	0.6197
	Scanner: DUN	0.6003	0.3097	1.9386	0.0535		-0.0027	1.2033
	Scanner: GLA	0.4711	0.2772	1.6997	0.0902		-0.0686	1.0109
	DMT w1: yes	-0.2397	0.1361	-1.7608	0.0793		-0.5047	0.0254
	WML	-0.0610	0.0263	-2.3181	0.0211		-0.1122	-0.0098
	Depression score	0.0119	0.0171	0.6951	0.4875		-0.0215	0.0453
	Time*Fatigue group	0.0202	0.0263	0.7673	0.4435	0.9389	-0.0310	0.0713
NAWM lateral orbitofrontal	Intercept	-0.3939	0.2577	-1.5287	0.1274		-0.8956	0.1078
	Time: w1	0.0218	0.0216	1.0065	0.3149		-0.0204	0.0639
	Fatigue group: fatigued	0.2385	0.1108	2.1534	0.0321		0.0228	0.4542
	Age	0.0470	0.0544	0.8624	0.3891		-0.0591	0.1530
	Sex: female	0.2730	0.1227	2.2252	0.0268		0.0341	0.5118
	Scanner: EDI1	0.3430	0.2705	1.2683	0.2057		-0.1836	0.8697
	Scanner: EDI2	-0.1945	0.2707	-0.7183	0.4731		-0.7216	0.3327
	Scanner: DUN	-0.0153	0.2998	-0.0511	0.9593		-0.5992	0.5685
	Scanner: GLA	-0.1789	0.2683	-0.6668	0.5054		-0.7013	0.3435
	DMT w1: yes	0.1722	0.1317	1.3074	0.1921		-0.0843	0.4286
	WML	-0.1033	0.0286	-3.6052	0.0004		-0.1591	-0.0475
	Depression score	0.0019	0.0192	0.1014	0.9193		-0.0354	0.0393
	Time*Fatigue group	-0.0832	0.0299	-2.7787	0.0058	0.2432	-0.1415	-0.0249
NAWM lingual	Intercept	-0.3515	0.2639	-1.3315	0.1840		-0.8654	0.1625
	Time: w1	-0.0675	0.0197	-3.4217	0.0007		-0.1059	-0.0291
	Fatigue group: fatigued	0.1054	0.1133	0.9302	0.3530		-0.1152	0.3260
	Age	-0.0348	0.0558	-0.6230	0.5338		-0.1434	0.0739
	Sex: female	0.2376	0.1257	1.8909	0.0596		-0.0071	0.4823
	Scanner: EDI1	0.3065	0.2771	1.1061	0.2695		-0.2331	0.8461
	Scanner: EDI2	-0.0905	0.2774	-0.3264	0.7444		-0.6307	0.4497
	Scanner: DUN	-0.0549	0.3071	-0.1788	0.8582		-0.6529	0.5431
	Scanner: GLA	0.0654	0.2749	0.2380	0.8121		-0.4698	0.6007
	DMT w1: yes	0.1292	0.1350	0.9570	0.3393		-0.1336	0.3919
	WML	-0.1011	0.0269	-3.7604	0.0002		-0.1534	-0.0487
	Depression score	-0.0080	0.0176	-0.4551	0.6494		-0.0424	0.0263
	Time*Fatigue group	-0.0153	0.0271	-0.5627	0.5740	0.9389	-0.0681	0.0376
NA [illegible]	Intercept	0.4458	0.2608	1.7093	0.0884		-0.0620	0.9537
	Time: w1	0.0225	0.0268	0.8394	0.4019		-0.0297	0.0746

	Fatigue group: fatigued	0.1711	0.1135	1.5076	0.1327		-0.0499	0.3922
	Age	0.0453	0.0551	0.8225	0.4114		-0.0620	0.1527
	Sex: female	-0.1136	0.1240	-0.9159	0.3605		-0.3551	0.1279
	Scanner: EDI1	-0.0837	0.2735	-0.3061	0.7597		-0.6162	0.4488
	Scanner: EDI2	-0.5018	0.2737	-1.8335	0.0677		-1.0348	0.0311
	Scanner: DUN	-0.6230	0.3032	-2.0547	0.0408		-1.2135	-0.0326
	Scanner: GLA	-0.3049	0.2712	-1.1241	0.2619		-0.8330	0.2233
	DMT w1: yes	-0.1643	0.1332	-1.2339	0.2182		-0.4236	0.0950
	WML	-0.1334	0.0337	-3.9603	0.0001		-0.1990	-0.0678
	Depression score	0.0011	0.0236	0.0482	0.9616		-0.0448	0.0470
	Time*Fatigue group	-0.0356	0.0373	-0.9534	0.3411	0.9389	-0.1083	0.0371
**NAWM middle temporal**	Intercept	0.0456	0.2707	0.1685	0.8663		-0.4814	0.5726
	Time: w1	-0.0569	0.0154	-3.6941	0.0003		-0.0869	-0.0269
	Fatigue group: fatigued	-0.1547	0.1156	-1.3382	0.1818		-0.3797	0.0704
	Age	-0.0816	0.0572	-1.4256	0.1550		-0.1930	0.0299
	Sex: female	0.2447	0.1289	1.8981	0.0586		-0.0063	0.4958
	Scanner: EDI1	0.0056	0.2843	0.0196	0.9844		-0.5481	0.5592
	Scanner: EDI2	-0.3864	0.2847	-1.3573	0.1757		-0.9407	0.1679
	Scanner: DUN	-0.3287	0.3150	-1.0436	0.2975		-0.9420	0.2846
	Scanner: GLA	-0.2829	0.2820	-1.0032	0.3166		-0.8321	0.2662
	DMT w1: yes	0.1348	0.1385	0.9736	0.3310		-0.1348	0.4045
	WML	0.0027	0.0219	0.1233	0.9020		-0.0400	0.0454
	Depression score	0.0091	0.0139	0.6587	0.5106		-0.0179	0.0362
	Time*Fatigue group	0.0144	0.0211	0.6822	0.4956	0.9389	-0.0266	0.0554
**NAWM paracentral**	Intercept	0.1244	0.2565	0.4851	0.6280		-0.3750	0.6238
	Time: w1	0.0031	0.0195	0.1608	0.8724		-0.0348	0.0411
	Fatigue group: fatigued	0.0326	0.1107	0.2946	0.7685		-0.1830	0.2482
	Age	-0.0710	0.0542	-1.3098	0.1913		-0.1767	0.0346
	Sex: female	0.3251	0.1221	2.6634	0.0081		0.0874	0.5628
	Scanner: EDI1	-0.2616	0.2692	-0.9719	0.3319		-0.7857	0.2625
	Scanner: EDI2	-0.7151	0.2695	-2.6539	0.0084		-1.2398	-0.1904
	Scanner: DUN	-0.3838	0.2983	-1.2868	0.1991		-0.9646	0.1970
	Scanner: GLA	-0.3776	0.2670	-1.4144	0.1583		-0.8975	0.1423
	DMT w1: yes	0.0330	0.1311	0.2518	0.8014		-0.2223	0.2883
	WML	-0.1824	0.0265	-6.8730	0.0000		-0.2340	-0.1307
	Depression score	0.0233	0.0174	1.3375	0.1821		-0.0106	0.0573
	Time*Fatigue group	0.0154	0.0268	0.5730	0.5670	0.9389	-0.0369	0.0676
**NAWM parahippocampal**	Intercept	-0.2131	0.2615	-0.8150	0.4157		-0.7224	0.2961
	Time: w1	-0.0204	0.0218	-0.9343	0.3509		-0.0629	0.0221
	Fatigue group: fatigued	0.0400	0.1125	0.3556	0.7224		-0.1790	0.2590
	Age	0.0987	0.0553	1.7849	0.0753		-0.0090	0.2063
	Sex: female	0.3196	0.1245	2.5672	0.0107		0.0772	0.5620
	Scanner: EDI1	0.0726	0.2745	0.2645	0.7916		-0.4619	0.6071
	Scanner: EDI2	-0.1412	0.2748	-0.5138	0.6078		-0.6762	0.3939

	Scanner: DUN	-0.3355	0.3043	-1.1026	0.2711		-0.9280	0.2570
	Scanner: GLA	-0.0286	0.2723	-0.1052	0.9163		-0.5588	0.5016
	DMT w1: yes	0.0476	0.1337	0.3564	0.7218		-0.2127	0.3079
	WML	-0.0952	0.0290	-3.2802	0.0012		-0.1517	-0.0387
	Depression score	0.0182	0.0194	0.9369	0.3495		-0.0196	0.0560
	Time*Fatigue group	-0.0260	0.0302	-0.8596	0.3907	0.9389	-0.0848	0.0328
**NAWM pars opercularis**	Intercept	0.1212	0.2633	0.4603	0.6456		-0.3915	0.6340
	Time: w1	-0.0127	0.0127	-1.0062	0.3151		-0.0374	0.0119
	Fatigue group: fatigued	0.0142	0.1123	0.1265	0.8994		-0.2044	0.2328
	Age	-0.1205	0.0557	-2.1652	0.0312		-0.2289	-0.0121
	Sex: female	0.1471	0.1255	1.1726	0.2419		-0.0972	0.3914
	Scanner: EDI1	0.0834	0.2767	0.3014	0.7633		-0.4553	0.6221
	Scanner: EDI2	-0.5227	0.2770	-1.8870	0.0601		-1.0622	0.0167
	Scanner: DUN	-0.3141	0.3065	-1.0248	0.3063		-0.9109	0.2827
	Scanner: GLA	-0.2157	0.2744	-0.7859	0.4325		-0.7501	0.3187
	DMT w1: yes	-0.0180	0.1347	-0.1333	0.8941		-0.2803	0.2444
	WML	-0.0853	0.0182	-4.6756	0.0000		-0.1208	-0.0498
	Depression score	-0.0030	0.0114	-0.2626	0.7930		-0.0252	0.0192
	Time*Fatigue group	0.0056	0.0172	0.3229	0.7470	0.9997	-0.0280	0.0391
**NAWM pars orbitalis**	Intercept	0.2327	0.2698	0.8624	0.3891		-0.2927	0.7581
	Time: w1	-0.0402	0.0249	-1.6113	0.1081		-0.0887	0.0084
	Fatigue group: fatigued	0.0269	0.1172	0.2292	0.8189		-0.2014	0.2551
	Age	0.0117	0.0570	0.2060	0.8370		-0.0993	0.1228
	Sex: female	-0.2196	0.1283	-1.7114	0.0880		-0.4695	0.0303
	Scanner: EDI1	0.1828	0.2830	0.6460	0.5188		-0.3682	0.7338
	Scanner: EDI2	-0.3394	0.2832	-1.1983	0.2318		-0.8910	0.2122
	Scanner: DUN	-0.2487	0.3137	-0.7928	0.4285		-0.8595	0.3621
	Scanner: GLA	-0.1218	0.2807	-0.4340	0.6646		-0.6683	0.4247
	DMT w1: yes	0.0690	0.1378	0.5010	0.6167		-0.1993	0.3374
	WML	0.0028	0.0324	0.0853	0.9320		-0.0603	0.0659
	Depression score	0.0021	0.0221	0.0939	0.9253		-0.0410	0.0452
	Time*Fatigue group	0.0033	0.0346	0.0966	0.9231	0.9997	-0.0640	0.0707
**NAWM pars triangularis**	Intercept	-0.0185	0.2714	-0.0683	0.9456		-0.5471	0.5100
	Time: w1	-0.0168	0.0158	-1.0658	0.2874		-0.0475	0.0139
	Fatigue group: fatigued	0.1083	0.1159	0.9341	0.3510		-0.1174	0.3340
	Age	-0.0135	0.0574	-0.2351	0.8143		-0.1252	0.0983
	Sex: female	-0.0936	0.1293	-0.7241	0.4696		-0.3454	0.1582
	Scanner: EDI1	0.3387	0.2851	1.1878	0.2359		-0.2166	0.8939
	Scanner: EDI2	-0.2026	0.2855	-0.7096	0.4785		-0.7585	0.3533
	Scanner: DUN	0.0133	0.3159	0.0422	0.9664		-0.6018	0.6285
	Scanner: GLA	0.0272	0.2828	0.0960	0.9235		-0.5236	0.5779
	DMT w1: yes	0.0105	0.1389	0.0756	0.9398		-0.2599	0.2809
	WML	-0.0056	0.0224	-0.2509	0.8021		-0.0492	0.0379
	Depression score	-0.0169	0.0142	-1.1927	0.2339		-0.0446	0.0107
	Time*Fatigue group	-0.0046	0.0216	-0.2119	0.8323	0.9997	-0.0466	0.0374

NAWM pericalcarine	Intercept	-0.1160	0.2614	-0.4437	0.6575		-0.6249	0.3929
	Time: w1	-0.1022	0.0161	-6.3311	0.0000		-0.1336	-0.0708
	Fatigue group: fatigued	0.1063	0.1117	0.9515	0.3421		-0.1113	0.3239
	Age	-0.0632	0.0553	-1.1441	0.2535		-0.1708	0.0444
	Sex: female	0.1241	0.1245	0.9972	0.3195		-0.1183	0.3665
	Scanner: EDI1	0.1302	0.2745	0.4744	0.6355		-0.4043	0.6648
	Scanner: EDI2	-0.2451	0.2748	-0.8919	0.3731		-0.7803	0.2900
	Scanner: DUN	-0.2582	0.3041	-0.8490	0.3966		-0.8504	0.3340
	Scanner: GLA	-0.0210	0.2723	-0.0772	0.9385		-0.5512	0.5092
	DMT w1: yes	0.1282	0.1337	0.9593	0.3382		-0.1321	0.3886
	WML	-0.1626	0.0227	-7.1572	0.0000		-0.2068	-0.1183
	Depression score	-0.0005	0.0145	-0.0348	0.9722		-0.0288	0.0278
	Time*Fatigue group	-0.0214	0.0221	-0.9694	0.3331	0.9389	-0.0645	0.0216
NAWM postcentral	Intercept	0.4879	0.2559	1.9064	0.0575		-0.0104	0.9862
	Time: w1	-0.0448	0.0281	-1.5960	0.1115		-0.0995	0.0099
	Fatigue group: fatigued	-0.0507	0.1112	-0.4559	0.6488		-0.2673	0.1659
	Age	0.0500	0.0541	0.9247	0.3559		-0.0553	0.1553
	Sex: female	0.0732	0.1217	0.6017	0.5478		-0.1638	0.3102
	Scanner: EDI1	-0.0593	0.2683	-0.2211	0.8251		-0.5819	0.4632
	Scanner: EDI2	-0.8873	0.2685	-3.3041	0.0011		-1.4102	-0.3644
	Scanner: DUN	-0.7452	0.2976	-2.5038	0.0128		-1.3248	-0.1656
	Scanner: GLA	-0.5444	0.2662	-2.0452	0.0417		-1.0626	-0.0261
	DMT w1: yes	0.0036	0.1307	0.0274	0.9781		-0.2508	0.2580
	WML	-0.1285	0.0344	-3.7320	0.0002		-0.1955	-0.0614
	Depression score	0.0183	0.0245	0.7466	0.4559		-0.0294	0.0660
	Time*Fatigue group	0.0439	0.0393	1.1170	0.2649	0.9389	-0.0326	0.1204
NAWM posterior cingulate	Intercept	-0.1362	0.2255	-0.6038	0.5464		-0.5753	0.3030
	Time: w1	-0.0401	0.0159	-2.5196	0.0123		-0.0710	-0.0091
	Fatigue group: fatigued	0.1440	0.0971	1.4829	0.1391		-0.0451	0.3330
	Age	-0.1108	0.0476	-2.3282	0.0206		-0.2035	-0.0181
	Sex: female	0.2869	0.1074	2.6722	0.0079		0.0778	0.4960
	Scanner: EDI1	0.3834	0.2367	1.6195	0.1064		-0.0776	0.8443
	Scanner: EDI2	-0.3223	0.2370	-1.3603	0.1747		-0.7838	0.1391
	Scanner: DUN	-0.0662	0.2624	-0.2523	0.8010		-0.5772	0.4448
	Scanner: GLA	0.0087	0.2348	0.0369	0.9706		-0.4486	0.4659
	DMT w1: yes	-0.1814	0.1153	-1.5737	0.1166		-0.4058	0.0431
	WML	-0.2661	0.0215	-12.3600	0.0000		-0.3080	-0.2242
	Depression score	0.0075	0.0140	0.5325	0.5948		-0.0198	0.0348
	Time*Fatigue group	0.0038	0.0219	0.1730	0.8628	0.9997	-0.0389	0.0464
NAWM precentral	Intercept	0.2072	0.2556	0.8108	0.4181		-0.2904	0.7048
	Time: w1	-0.0018	0.0197	-0.0894	0.9288		-0.0402	0.0367
	Fatigue group: fatigued	0.0515	0.1097	0.4694	0.6391		-0.1621	0.2650
	Age	0.0686	0.0540	1.2706	0.2048		-0.0365	0.1738
	Sex: female	0.2147	0.1217	1.7648	0.0786		-0.0222	0.4517

	Scanner: EDI1	0.1375	0.2683	0.5125	0.6087		-0.3850	0.6600
	Scanner: EDI2	-0.5564	0.2686	-2.0714	0.0392		-1.0794	-0.0333
	Scanner: DUN	-0.1072	0.2974	-0.3605	0.7187		-0.6863	0.4719
	Scanner: GLA	-0.2678	0.2661	-1.0063	0.3151		-0.7861	0.2504
	DMT w1: yes	-0.2503	0.1307	-1.9156	0.0564		-0.5047	0.0041
	WML	-0.1505	0.0267	-5.6464	0.0000		-0.2024	-0.0986
	Depression score	0.0068	0.0176	0.3873	0.6988		-0.0275	0.0411
	Time*Fatigue group	0.0260	0.0272	0.9544	0.3406	0.9389	-0.0270	0.0790
**NAWM precuneus**	Intercept	0.5044	0.2597	1.9421	0.0530		-0.0013	1.0101
	Time: w1	-0.0715	0.0153	-4.6636	0.0000		-0.1013	-0.0416
	Fatigue group: fatigued	-0.0368	0.1110	-0.3312	0.7408		-0.2529	0.1794
	Age	0.0259	0.0549	0.4712	0.6378		-0.0811	0.1328
	Sex: female	-0.0535	0.1237	-0.4325	0.6657		-0.2944	0.1874
	Scanner: EDI1	-0.2441	0.2728	-0.8947	0.3716		-0.7753	0.2871
	Scanner: EDI2	-0.4093	0.2731	-1.4986	0.1350		-0.9412	0.1225
	Scanner: DUN	-0.5463	0.3022	-1.8075	0.0717		-1.1348	0.0422
	Scanner: GLA	-0.4131	0.2706	-1.5268	0.1279		-0.9400	0.1138
	DMT w1: yes	-0.0670	0.1329	-0.5046	0.6142		-0.3258	0.1917
	WML	-0.1726	0.0217	-7.9538	0.0000		-0.2149	-0.1304
	Depression score	0.0179	0.0138	1.2969	0.1956		-0.0090	0.0448
	Time*Fatigue group	0.0265	0.0210	1.2624	0.2078	0.9389	-0.0143	0.0673
**NAWM rostral anterior cingulate**	Intercept	0.2859	0.2657	1.0759	0.2828		-0.2315	0.8032
	Time: w1	-0.0254	0.0214	-1.1860	0.2365		-0.0670	0.0163
	Fatigue group: fatigued	0.0559	0.1150	0.4862	0.6272		-0.1680	0.2798
	Age	-0.0753	0.0562	-1.3403	0.1811		-0.1847	0.0341
	Sex: female	0.1868	0.1264	1.4775	0.1406		-0.0594	0.4329
	Scanner: EDI1	-0.3338	0.2788	-1.1973	0.2321		-0.8766	0.2090
	Scanner: EDI2	-0.7976	0.2791	-2.8582	0.0046		-1.3410	-0.2542
	Scanner: DUN	-0.6296	0.3089	-2.0383	0.0424		-1.2312	-0.0281
	Scanner: GLA	-0.5263	0.2765	-1.9035	0.0579		-1.0647	0.0121
	DMT w1: yes	0.1221	0.1358	0.8994	0.3691		-0.1423	0.3865
	WML	-0.0755	0.0288	-2.6238	0.0091		-0.1316	-0.0195
	Depression score	0.0142	0.0191	0.7434	0.4578		-0.0230	0.0514
	Time*Fatigue group	0.0150	0.0295	0.5072	0.6124	0.9389	-0.0425	0.0725
**NAWM rostral middle frontal**	Intercept	-0.0514	0.2625	-0.1960	0.8447		-0.5625	0.4596
	Time: w1	-0.0065	0.0143	-0.4582	0.6471		-0.0343	0.0212
	Fatigue group: fatigued	0.0056	0.1120	0.0499	0.9602		-0.2125	0.2237
	Age	-0.0572	0.0555	-1.0312	0.3033		-0.1652	0.0508
	Sex: female	-0.1753	0.1250	-1.4022	0.1619		-0.4188	0.0681
	Scanner: EDI1	0.4519	0.2757	1.6391	0.1022		-0.0850	0.9888
	Scanner: EDI2	-0.3214	0.2760	-1.1644	0.2452		-0.8590	0.2161
	Scanner: DUN	0.1478	0.3055	0.4840	0.6288		-0.4470	0.7426
	Scanner: GLA	0.1864	0.2735	0.6815	0.4961		-0.3462	0.7189
	DMT w1: yes	0.0999	0.1343	0.7438	0.4576		-0.1616	0.3613
	WML	-0.0524	0.0203	-2.5801	0.0103		-0.0919	-0.0129

	Depression score	-0.0056	0.0128	-0.4396	0.6606		-0.0306	0.0193
	Time*Fatigue group	-0.0205	0.0195	-1.0523	0.2935	0.9389	-0.0584	0.0174
**NAWM superior frontal**	Intercept	0.3505	0.2569	1.3641	0.1735		-0.1498	0.8508
	Time: w1	-0.0102	0.0204	-0.4991	0.6180		-0.0499	0.0295
	Fatigue group: fatigued	0.0721	0.1103	0.6534	0.5140		-0.1427	0.2868
	Age	0.0105	0.0543	0.1932	0.8469		-0.0952	0.1162
	Sex: female	0.0474	0.1223	0.3876	0.6986		-0.1908	0.2856
	Scanner: EDI1	-0.0663	0.2697	-0.2459	0.8059		-0.5916	0.4589
	Scanner: EDI2	-0.9848	0.2700	-3.6470	0.0003		-1.5105	-0.4590
	Scanner: DUN	-0.5183	0.2990	-1.7333	0.0841		-1.1005	0.0640
	Scanner: GLA	-0.4451	0.2676	-1.6634	0.0973		-0.9661	0.0759
	DMT w1: yes	0.0819	0.1314	0.6235	0.5334		-0.1739	0.3377
	WML	-0.0778	0.0273	-2.8445	0.0047		-0.1310	-0.0245
	Depression score	0.0051	0.0181	0.2792	0.7803		-0.0303	0.0404
	Time*Fatigue group	-0.0098	0.0282	-0.3492	0.7272	0.9997	-0.0647	0.0450
**NAWM superior parietal**	Intercept	-0.0826	0.2604	-0.3173	0.7512		-0.5897	0.4245
	Time: w1	-0.0029	0.0140	-0.2107	0.8332		-0.0301	0.0242
	Fatigue group: fatigued	0.0235	0.1113	0.2108	0.8332		-0.1932	0.2401
	Age	0.0134	0.0550	0.2431	0.8081		-0.0938	0.1205
	Sex: female	0.2739	0.1241	2.2079	0.0280		0.0323	0.5155
	Scanner: EDI1	0.0400	0.2736	0.1464	0.8837		-0.4926	0.5727
	Scanner: EDI2	-0.2768	0.2739	-1.0107	0.3129		-0.8102	0.2565
	Scanner: DUN	-0.5429	0.3031	-1.7911	0.0743		-1.1331	0.0473
	Scanner: GLA	-0.0749	0.2714	-0.2758	0.7829		-0.6033	0.4536
	DMT w1: yes	0.0142	0.1332	0.1063	0.9154		-0.2452	0.2736
	WML	-0.1403	0.0198	-7.0759	0.0000		-0.1790	-0.1017
	Depression score	0.0125	0.0125	0.9986	0.3188		-0.0119	0.0368
	Time*Fatigue group	0.0025	0.0191	0.1298	0.8968	0.9997	-0.0346	0.0396
**NAWM superior temporal**	Intercept	0.3820	0.2686	1.4221	0.1560		-0.1410	0.9049
	Time: w1	-0.0506	0.0171	-2.9582	0.0033		-0.0840	-0.0173
	Fatigue group: fatigued	0.0903	0.1148	0.7864	0.4323		-0.1333	0.3139
	Age	-0.0088	0.0568	-0.1550	0.8769		-0.1193	0.1017
	Sex: female	-0.1294	0.1279	-1.0114	0.3126		-0.3785	0.1197
	Scanner: EDI1	-0.0766	0.2821	-0.2715	0.7862		-0.6259	0.4727
	Scanner: EDI2	-0.4939	0.2824	-1.7490	0.0813		-1.0439	0.0560
	Scanner: DUN	-0.3078	0.3126	-0.9847	0.3256		-0.9165	0.3009
	Scanner: GLA	-0.4250	0.2798	-1.5188	0.1299		-0.9698	0.1199
	DMT w1: yes	0.0353	0.1374	0.2572	0.7972		-0.2322	0.3028
	WML	-0.0386	0.0239	-1.6163	0.1071		-0.0851	0.0079
	Depression score	0.0045	0.0153	0.2935	0.7693		-0.0254	0.0344
	Time*Fatigue group	0.0110	0.0235	0.4685	0.6398	0.9389	-0.0347	0.0567
**NAWM**	Intercept	0.4420	0.2632	1.6793	0.0941		-0.0705	0.9545
	Time: w1	-0.0058	0.0126	-0.4574	0.6477		-0.0302	0.0187
	Fatigue group: fatigued	0.0902	0.1129	0.7985	0.4252		-0.1297	0.3100

	Age	0.0238	0.0556	0.4283	0.6687		-0.0844	0.1320
	Sex: female	-0.1704	0.1253	-1.3599	0.1749		-0.4145	0.0736
	Scanner: EDI1	-0.1611	0.2764	-0.5828	0.5605		-0.6993	0.3771
	Scanner: EDI2	-0.5254	0.2767	-1.8985	0.0586		-1.0642	0.0135
	Scanner: DUN	-0.6718	0.3062	-2.1939	0.0290		-1.2681	-0.0755
	Scanner: GLA	-0.5128	0.2742	-1.8705	0.0624		-1.0467	0.0210
	DMT w1: yes	0.1026	0.1346	0.7627	0.4463		-0.1594	0.3647
	WML	-0.0775	0.0179	-4.3178	0.0000		-0.1124	-0.0425
	Depression score	0.0147	0.0112	1.3119	0.1905		-0.0071	0.0365
	Time*Fatigue group	0.0051	0.0172	0.2955	0.7678	0.9997	-0.0284	0.0385
**NAWM temporal pole**	Intercept	0.3985	0.2485	1.6033	0.1099		-0.0854	0.8824
	Time: w1	-0.0708	0.0405	-1.7482	0.0814		-0.1497	0.0081
	Fatigue group: fatigued	-0.0270	0.1101	-0.2452	0.8065		-0.2415	0.1875
	Age	0.0638	0.0524	1.2171	0.2245		-0.0383	0.1659
	Sex: female	-0.1036	0.1180	-0.8781	0.3806		-0.3334	0.1262
	Scanner: EDI1	0.2600	0.2601	0.9996	0.3183		-0.2465	0.7664
	Scanner: EDI2	-0.3797	0.2601	-1.4596	0.1454		-0.8863	0.1269
	Scanner: DUN	-0.5475	0.2888	-1.8958	0.0589		-1.1098	0.0149
	Scanner: GLA	-0.1069	0.2580	-0.4144	0.6789		-0.6092	0.3954
	DMT w1: yes	-0.2067	0.1266	-1.6332	0.1035		-0.4532	0.0398
	WML	-0.1405	0.0410	-3.4220	0.0007		-0.2204	-0.0605
	Depression score	0.0365	0.0330	1.1063	0.2695		-0.0277	0.1007
	Time*Fatigue group	0.0394	0.0579	0.6805	0.4967	0.9389	-0.0733	0.1520
**NAWM transverse temporal**	Intercept	-0.3463	0.2683	-1.2908	0.1977		-0.8686	0.1761
	Time: w1	-0.0455	0.0276	-1.6489	0.1002		-0.0993	0.0082
	Fatigue group: fatigued	0.1191	0.1167	1.0209	0.3081		-0.1081	0.3464
	Age	0.0488	0.0567	0.8609	0.3900		-0.0616	0.1592
	Sex: female	0.3827	0.1276	2.9999	0.0029		0.1343	0.6311
	Scanner: EDI1	0.0628	0.2813	0.2233	0.8235		-0.4849	0.6105
	Scanner: EDI2	0.0319	0.2815	0.1134	0.9098		-0.5163	0.5801
	Scanner: DUN	0.0258	0.3119	0.0826	0.9342		-0.5816	0.6331
	Scanner: GLA	0.0933	0.2790	0.3343	0.7384		-0.4500	0.6365
	DMT w1: yes	-0.0414	0.1370	-0.3021	0.7628		-0.3081	0.2253
	WML	-0.0504	0.0347	-1.4506	0.1479		-0.1180	0.0172
	Depression score	0.0376	0.0243	1.5481	0.1226		-0.0097	0.0850
	Time*Fatigue group	-0.0096	0.0385	-0.2485	0.8039	0.9997	-0.0846	0.0654
**WML**	Intercept	-0.3315	0.2639	-1.2558	0.2101		-0.8458	0.1829
	Time: w1	0.2911	0.0338	8.6200	0.0000		0.2253	0.3569
	Fatigue group: fatigued	-0.0381	0.1189	-0.3205	0.7488		-0.2699	0.1937
	Age	0.1403	0.0555	2.5273	0.0120		0.0321	0.2485
	Sex: female	-0.1341	0.1252	-1.0714	0.2848		-0.3780	0.1098
	Scanner: EDI1	0.2573	0.2760	0.9321	0.3520		-0.2806	0.7952
	Scanner: EDI2	0.1077	0.2764	0.3898	0.6970		-0.4309	0.6463
	Scanner: DUN	0.5037	0.3057	1.6474	0.1005		-0.0921	1.0995
	Scanner: GLA	0.1782	0.2739	0.6506	0.5158		-0.3555	0.7119

	DMT w1: yes	0.1450	0.1344	1.0792	0.2813		-0.1169	0.4070
	Depression score	0.0233	0.0304	0.7650	0.4448		-0.0360	0.0826
	Time*Fatigue group	-0.0406	0.0505	-0.8029	0.4227		-0.1390	0.0579
**Whole-brain**	Intercept	-0.3371	0.2282	-1.4770	0.1407	0.9389	-0.7818	0.1076
	Time: w1	-0.0878	0.0197	-4.4555	0.0000		-0.1262	-0.0494
	Fatigue group: fatigued	-0.0396	0.0986	-0.4014	0.6884		-0.2317	0.1525
	Age	-0.3648	0.0482	-7.5723	0.0000		-0.4587	-0.2709
	Sex: female	0.3364	0.1086	3.0978	0.0021		0.1248	0.5481
	Scanner: EDI1	0.7530	0.2396	3.1431	0.0018		0.2861	1.2198
	Scanner: EDI2	-0.3729	0.2399	-1.5545	0.1211		-0.8403	0.0946
	Scanner: DUN	0.0282	0.2652	0.1065	0.9153		-0.4887	0.5451
	Scanner: GLA	0.1141	0.2376	0.4801	0.6315		-0.3490	0.5772
	DMT w1: yes	0.0317	0.1166	0.2716	0.7862		-0.1956	0.2590
	Depression score	-0.0095	0.0188	-0.5035	0.6149		-0.0461	0.0272
	Time*Fatigue group	-0.0334	0.0295	-1.1315	0.2587	0.9389	-0.0909	0.0241
**SCCSA-C2-3**	Intercept	0.6361	0.2428	2.6199	0.0092		0.1629	1.1093
	Time: w1	-0.3018	0.0587	-5.1429	0.0000		-0.4161	-0.1874
	Fatigue group: fatigued	-0.1190	0.1165	-1.0212	0.3079		-0.3460	0.1081
	Age	-0.1438	0.0503	-2.8592	0.0045		-0.2418	-0.0458
	Sex: female	-0.1647	0.1138	-1.4473	0.1488		-0.3864	0.0571
	Scanner: EDI1	-0.5918	0.2458	-2.4073	0.0166		-1.0709	-0.1126
	Scanner: EDI2	-0.6502	0.2456	-2.6470	0.0085		-1.1290	-0.1714
	Scanner: DUN	-0.2086	0.2715	-0.7684	0.4428		-0.7378	0.3206
	Scanner: GLA	-0.4533	0.2424	-1.8698	0.0624		-0.9258	0.0193
	DMT w1: yes	0.2071	0.1239	1.6721	0.0955		-0.0343	0.4486
	Depression score	-0.0430	0.0426	-1.0103	0.3131		-0.1260	0.0400
	Time*Fatigue group	0.0697	0.0876	0.7951	0.4271	0.4271	-0.1011	0.2404

RRMS=relapsing-remitting multiple sclerosis, FDR=false discovery rate, $B_{standardised}$=standardised beta value, SE=standard error, CI=confidence interval for beta value, w0=baseline, w1=1-year follow-up, GM=grey matter, NAWM=normal-appearing white matter, sts=superior temporal sulcus, WML=white matter lesion, ED1=Edinburgh scanner 1, EDI2=Edinburgh scanner 2, DUN=Dundee, GLA=Glasgow, SCCSA-C2-3=spinal cord cross-sectional area cervical levels 2 and 3, PHQ-9=patient health questionnaire, FSS=fatigue severity scale, DMT=disease-modifying treatment

Supplementary Table 6. Results for differences in new/enlarging WMLs (yes/no) at follow-up between RRMS participants with and without fatigue (based on FSS score). This was assessed using logistic regression, with fatigue group as regressor of interest and MRI scanner, age_{w0}, sex, DMT intake at w1, and depression score (based on PHQ-9) as control variables.

		$B_{standardised}$	SD	t-value	p	CI 2.5%	CI 97.5%	OR	OR CI 2.5%	OR CI 97.5%
New/enlarging WML (yes/no)	Intercept	-1.0203	0.7102	-1.4367	0.1508	-2.5988	0.2671	0.3605	0.0744	1.3062
	Fatigue group: fatigued	-0.3245	0.2842	-1.1418	0.2535	-0.8860	0.2305	0.7229	0.4123	1.2592
	Age	-0.5762	0.1304	-4.4188	**<0.001**	-0.8385	-0.3262	0.5621	0.4324	0.7217
	Sex: female	0.2384	0.2802	0.8507	0.3949	-0.3102	0.7913	1.2692	0.7333	2.2062
	Scanner: EDI1	1.4199	0.7224	1.9657	**0.0493**	0.1035	3.0155	4.1638	1.1150	20.5254
	Scanner: EDI2	1.4264	0.7226	1.9742	**0.0484**	0.1088	3.0217	4.1368	1.1091	20.3992
	Scanner: DUN	1.0864	0.7838	1.3860	0.1657	-0.3693	2.7765	2.9636	0.6912	16.0635
	Scanner: GLA	1.1802	0.7209	1.6372	0.1016	-0.1313	2.7745	3.2552	0.8769	16.0312
	DMT w1: yes	-0.3443	0.3003	-1.1466	0.2515	-0.9393	0.2411	0.7087	0.3909	1.2727
	Depression score	0.3702	0.1446	2.5594	**0.0105**	0.0915	0.6602	1.4480	1.0958	1.9352

RRMS=relapsing-remitting multiple sclerosis, $B_{standardised}$=standardised beta value, SE=standard error, CI=confidence interval for beta value, OR=odds ratio, w0=baseline, w1=1-year follow-up, WML=white matter lesion, ED1=Edinburgh scanner 1, EDI2=Edinburgh scanner 2, DUN=Dundee, GLA=Glasgow, PHQ-9=patient health questionnaire, FSS=fatigue severity scale, DMT=disease-modifying treatment
Significant p-values (p<.05) are highlighted in bold.

Supplementary Table 7. The effect of time point (w0, w1) on [A] WB and [B] WML volume, and [C] SCCSA-C2-3, for fatigue groups separately. This was assessed with post-hoc linear regression models, with age at w0, sex, MRI scanner, DMT status at w1 and depression score (based on PHQ-9) as control variables.

	$B_{standardised}$	SE	t	p	P_{FDR}	CI 2.5.	CI 97.5.	$B_{standardised}$	SE	t	P	P_{FDR}	CI 2.5.	CI 97.5.
**A**	*WB volume change: non-fatigued*							*WB volume change: fatigued*						
(Intercept)	-0.4003	0.2675	-1.4962	0.1365		-0.9150	0.1144	-0.3301	0.4189	-0.7882	0.4320		-1.1326	0.4726
Time point	-0.0849	0.0187	-4.5423	**<0.0001**	**0.0001**	-0.1216	-0.0483	-0.1251	0.0256	-4.8918	**<0.0001**	**<0.0001**	-0.1755	-0.0753
Scanner: EDI1	1.0516	0.2959	3.5534	**0.0005**		0.4823	1.6210	0.3501	0.4159	0.8417	0.4014		-0.4468	1.1471
Scanner: EDI2	0.0614	0.2994	0.2049	0.8379		-0.5147	0.6374	-0.8046	0.4146	-1.9407	0.0544		-1.5991	-0.0103
Scanner: DUN	0.2827	0.3577	0.7902	0.4306		-0.4056	0.9709	-0.3314	0.4322	-0.7667	0.4446		-1.1595	0.4968
Scanner: GLA	0.5534	0.2958	1.8707	0.0632		-0.0157	1.1225	-0.3887	0.4120	-0.9434	0.3472		-1.1780	0.4007
Sex: female	0.2287	0.1460	1.5662	0.1192		-0.0522	0.5096	0.4053	0.1670	2.4274	**0.0165**		0.0854	0.7252
DMT w1: yes	-0.2817	0.1469	-1.9181	0.0568		-0.5643	0.0009	0.4493	0.1937	2.3193	**0.0219**		0.0780	0.8204
Age	-0.2864	0.0660	-4.3420	**<0.0001**		-0.4133	-0.1595	-0.4261	0.0716	-5.9535	**<0.0001**		-0.5632	-0.2890
Depression score	0.0099	0.0290	0.3404	0.7339		-0.0470	0.0665	-0.0181	0.0254	-0.7139	0.4763		-0.0685	0.0309
**B**	*SCCSA-C2-3 change: non-fatigued*							*SCCSA-C2-3 change: fatigued*						
(Intercept)	0.6870	0.2897	2.3719	0.0188		0.1298	1.2441	0.6900	0.4503	1.5321	0.1278		-0.1707	1.5546
Time point	-0.2910	0.0566	-5.1407	**<0.0001**	**<0.0001**	-0.4021	-0.1800	-0.2615	0.0711	-3.6758	**0.0003**	**0.0024**	-0.4045	-0.1245
Scanner: EDI1	-0.6360	0.2996	-2.1225	**0.0353**		-1.2121	-0.0598	-0.6643	0.4413	-1.5053	0.1345		-1.5086	0.1812
Scanner: EDI2	-0.6146	0.2972	-2.0683	**0.0401**		-1.1860	-0.0432	-0.9148	0.4469	-2.0471	**0.0426**		-1.7711	-0.0599
Scanner: DUN	-0.5429	0.3648	-1.4882	0.1386		-1.2444	0.1586	-0.1639	0.4567	-0.3589	0.7202		-1.0378	0.7110
Scanner: GLA	-0.4762	0.2955	-1.6113	0.1090		-1.0445	0.0921	-0.5763	0.4374	-1.3176	0.1899		-1.4135	0.2612
Sex: female	-0.0588	0.1519	-0.3874	0.6989		-0.3509	0.2332	-0.3380	0.1779	-1.9002	0.0595		-0.6790	0.0022
DMT w1: yes	0.1197	0.1603	0.7466	0.4563		-0.1885	0.4279	0.3407	0.2046	1.6651	0.0982		-0.0515	0.7320
Age	-0.1398	0.0689	-2.0273	**0.0442**		-0.2723	-0.0072	-0.1098	0.0765	-1.4351	0.1536		-0.2562	0.0367
Depression score	0.0535	0.0706	0.7575	0.4493		-0.0837	0.1902	-0.1051	0.0555	-1.8929	0.0594		-0.2200	0.0014
**C**	*WML volume change: non-fatigued*							*WML change volume: fatigued*						
(Intercept)	-0.4813	0.3013	-1.5974	0.1121		-1.0609	0.0985	-0.0782	0.5016	-0.1558	0.8764		-1.0388	0.8825
Time point	0.2885	0.0249	11.5719	**<0.0001**	**<0.0001**	0.2397	0.3374	0.2508	0.0503	4.9851	**<0.0001**	**<0.0001**	0.1523	0.3493
Scanner: EDI1	0.1808	0.3327	0.5435	0.5875		-0.4593	0.8209	0.2957	0.4968	0.5952	0.5527		-0.6556	1.2470

Scanner: EDI2	-0.1749	0.3367	-0.5196	0.6041		-0.8227	0.4727	0.2909	0.4952	0.5875	0.5579		-0.6574	1.2393
Scanner: DUN	0.0831	0.4023	0.2065	0.8367		-0.6908	0.8568	0.7923	0.5162	1.5348	0.1272		-0.1963	1.7809
Scanner: GLA	-0.0784	0.3326	-0.2357	0.8140		-0.7184	0.5615	0.3533	0.4920	0.7180	0.4740		-0.5889	1.2954
Sex: female	0.0746	0.1642	0.4545	0.6501		-0.2413	0.3905	-0.3042	0.1994	-1.5256	0.1295		-0.6860	0.0776
DMT w1: yes	0.4517	0.1652	2.7345	**0.0069**		0.1339	0.7694	-0.2512	0.2316	-1.0845	0.2801		-0.6948	0.1924
Age	0.1389	0.0742	1.8727	0.0629		-0.0038	0.2816	0.1374	0.0856	1.6061	0.1106		-0.0264	0.3012
Depression score	0.0058	0.0381	0.1517	0.8796		-0.0682	0.0808	0.0239	0.0463	0.5159	0.6064		-0.0661	0.1137

RRMS=relapsing-remitting multiple sclerosis, $B_{standardised}$=standardised beta value, SE=standard error, CI=confidence interval for beta value, w0=baseline, w1=1-year follow-up, EDI1=Edinburgh scanner 1, EDII2=Edinburgh scanner 2, DUN=Dundee, GLA=Glasgow, WB=whole-brain , WML=white matter lesion, SCCSA-C2-3=spinal cord cross-sectional area cervical levels 2 and 3, PHQ-9=patient health questionnaire, FSS=fatigue severity scale, DMT=disease-modifying treatment, FDR = false discovery rate
Significant p-values (p<.05) are highlighted in bold.
